# Central role for RNase YbeY in Hfq-dependent and Hfq-independent small-RNA regulation in bacteria

**DOI:** 10.1186/1471-2164-15-121

**Published:** 2014-02-11

**Authors:** Shree P Pandey, Jonathan A Winkler, Hu Li, Diogo M Camacho, James J Collins, Graham C Walker

**Affiliations:** 1 Department of Biological Sciences, Indian Institute of Science Education and Research Kolkata, Mohanpur Campus, Mohanpur, Nadia, West Bengal 741252, India; 2 Department of Biomedical Engineering, Center for BioDynamics and Center for Advanced Biotechnology, Howard Hughes Medical Institute, Boston University, Boston, MA, USA; 3 Department of Biology, Massachusetts Institute of Technology, Cambridge, MA 02315, USA; 4 Current address: Center for Individualized Medicine, Department of Molecular Pharmacology & Experimental Therapeutics, Mayo Clinic, Rochester, MN 55905, USA

**Keywords:** YbeY, Small-RNA, Hfq, Hydroxyurea, Stress adaptation, *E. coli*

## Abstract

**Background:**

Conceptual parallels exist between bacterial and eukaryotic small-RNA (sRNA) pathways, yet relatively little is known about which protein may recognize and recruit bacterial sRNAs to interact with targets. In eukaryotes, Argonaute (AGO) proteins discharge such functions. The highly conserved bacterial YbeY RNase has structural similarities to the MID domain of AGOs. A limited study had indicated that in *Sinorhizobium meliloti* the YbeY ortholog regulates the accumulation of sRNAs as well as the target mRNAs, raising the possibility that YbeY may play a previously unrecognized role in bacterial sRNA regulation.

**Results:**

We have applied a multipronged approach of loss-of-function studies, genome-wide mRNA and sRNA expression profiling, pathway analysis, target prediction, literature mining and network analysis to unravel YbeY-dependent molecular responses of *E. coli* exposed to hydroxyurea (HU). Loss of *ybeY* function, which results in a marked resistance to HU, had global affects on sRNA-mediated gene expression. Of 54 detectable *E. coli* sRNAs in our microarray analysis, 30 sRNAs showed a differential expression upon HU stress, of which 28 sRNAs displayed a YbeY-dependent change in expression. These included 12 Hfq-dependent and 16 Hfq-independent sRNAs. We successfully identified at least 57 experimentally inferred sRNA-mRNA relationships. Further applying a ‘context likelihood of relatedness’ algorithm, we reverse engineered the YbeY-dependent Hfq-dependent sRNA-mRNA network as well as YbeY-dependent Hfq-independent sRNA-mRNA network.

**Conclusion:**

YbeY extensively modulates Hfq-dependent and independent sRNA-mRNA interactions. YbeY-dependent sRNAs have central roles in modulating cellular response to HU stress.

## Background

Small regulatory RNAs (sRNAs) play key roles in modulating gene expression in both prokaryotes and eukaryotes. In bacteria, numerous sRNAs that range in size from ~50-300 nucleotides in length act on independently transcribed mRNA targets. In *E. coli*, approximately 80 such sRNAs have been validated [1]. The most extensively characterized class of bacterial sRNAs are the *trans*-encoded sRNAs that are encoded distant from the genes for their mRNA targets and that typically have only limited complementarity (10–30 nt) [2]. *Trans-*encoded sRNAs form imperfect base-pairing interactions with complementary sequences in their mRNA targets, which are often located at or near ribosome binding sites (RBS), but can also be located upstream of the translation start site as well as deep in the coding regions (CDS) [2]. Such interactions generally result in a decrease in protein synthesis, either by stimulating mRNA degradation or by inhibiting translation. Other bacterial sRNAs, referred to as *cis*-encoded sRNAs, are present in close proximity to their targets, such as upstream, opposite of the 5′ UTR of the target, or between two genes in an operon [2]. Such sRNAs have extensive (>75 nt) complementarity to their targets [2].

Gaining a deeper insight into how sRNAs recognize and interact with their targets is crucial to understanding the mechanism of sRNA action and function in bacteria at a molecular level. For certain sRNAs (e.g. RNAIII, RsaE, and SprD in *Staphylococcus aureus* as well as CyaR sRNAs), target recognition is structure-driven using C-rich stretches that are located within accessible loop regions [3]. For many other *trans*-acting sRNAs, however, the interacting region is not located in a structured region, rather in a single-stranded region that is often located at the 5′ end of the sRNA. Like microRNAs (miRNAs) in eukaryotes, *trans*-acting bacterial sRNAs appear to recognize their targets by a seed-pairing mechanism using seeds as small as 6–7 nucleotides. Fusion studies have revealed that seed regions of RybB or MicC sRNAs are sufficient to guide the recognition of targets [3]. Moreover, as in miRNAs, 3′ adenosine (A) residues have been reported recently to occur adjacent to the pairing region [3,4].

In numerous bacteria, mainly Gram negatives, the RNA-binding protein Hfq is required for the action and stability of many *trans*-encoded sRNAs [5,6]. The Hfq chaperone binds to A/U rich regions of sRNAs that are often located near the stem-loop structures as well as to the poly (U) regions at the 3′ end of the sRNAs [7]. Structural and binding studies have revealed several RNA binding sites on the proximal as well as distal faces of Hfq hexameric ring, which may facilitate the interaction of sRNA and their target mRNAs [7]. Hfq may assist duplex formation by enhancing local RNA concentrations, changing RNA structures and accelerating strand exchange and annealing. Although most of the *E. coli* trans-acting sRNAs that have been characterized require Hfq for base pairing, some in *Vibrio cholerae* do not require Hfq for pairing with target [8]. Loss-of-function studies suggest that Hfq is essential for virulence of several pathogens as well as symbiosis of *Sinorhizobium meliloti* with plants [5,9].

Hfq interacts with RNase E to serve to recruit the RNA degradation machinery once the sRNAs have base-paired with targets [10]. Moreover, recent work has shown that a 5′-monophosphorylated sRNA seed both guides RNase E to its mRNA target and also stimulates the degradation. Hfq is needed for optimal RNase E activity in this sRNA-guided mRNA cleavage [11]. RNase III also participates in sRNA-mediated modulation of mRNAs [3]. Further, RNases also play an important role in generation of mature sRNAs. For example, 6S RNA maturation involves multi-layered pathway involving endonucleolytic digestion by RNase E or G and exonucleolytic trimming at 5′ and 3′ ends [3].

Although Hfq is known to bind to the C-terminus of RNase E and recruit it to sRNA-mediated interactions, much remains to be learned concerning the molecular mechanism and function of various RNases and other RNA binding proteins during bacterial RNA-interference. Recent progress has offered insights into bacterial sRNAs that are recognized and loaded on to the Hfq protein scaffold during their interaction with the target mRNAs [12-14] but the identity of the proteins that facilitate sRNA-mRNA interactions in bacteria lacking Hfq or facilitate the interactions of the sRNAs that are Hfq-independent remains largely unknown.

Striking conceptual parallels exist between the bacterial and the eukaryotic sRNA-pathways. In eukaryotes, numerous structural studies have revealed the molecular details of how the miRNAs and siRNAs are recognized by, and loaded onto, the Argonaute proteins and then guided to the targets by the RNA-induced silencing complex (RISC) [15]. In contrast, considerably less is known about the molecular details of how bacterial sRNAs recognize their target mRNAs or about the roles of Hfq and other proteins in this process.

We have recently reported evidence that the highly conserved bacterial protein YbeY (SMc01113 in *Sinorhizobium meliloti*) may play a major, previously unrecognized role in bacterial sRNA regulation [16]. *ybeY*, which is one of the 206 genes that comprise the postulated minimal bacterial genome set [17], is essential in some bacteria [18,19]. In contrast, in certain other bacteria such as *Escherichia coli* and *Sinorhizobium meliloti*, *ybeY* is not essential but its loss sensitizes cells to a wide variety of physiologically diverse stresses and causes striking defects that affect ribosome activity, translational fidelity and ribosome assembly [20-22]. Several observations had suggested that YbeY might interact with RNA. Our mapping of 16S, 23S and 5S rRNA termini in an *E. coli ΔybeY* mutant showed that YbeY influences the maturation of all three rRNAs, with a particularly strong effect on maturation at both the 5′- and 3′-end of 16S rRNA as well as maturation of the 5′-termini of 23S and 5S rRNAs [23]. Furthermore, we demonstrated that there are strong genetic interactions between *ybeY* and *rnc* (encoding RNase III), *ybeY* and *rnr* (encoding RNase R), and *ybeY* and *pnp* (encoding PNPase) [23].

We have recently shown that YbeY is a previously undiscovered single-strand RNase with a combination of biochemical properties that distinguish it from all previously reported RNases [24]. Additionally, we have shown that YbeY plays in a key role in a previously unrecognized system of 70S ribosome quality control, in which YbeY and RNase R act together to degrade defective 70S ribosomes but not properly matured 70S ribosomes or individual subunits. In addition, we discovered that there is essentially no fully matured 16S rRNA in a ∆*ybeY* mutant at 45°C, making YbeY the first endoribonuclease to be implicated in the critically important processing of the 16S rRNA 3′ terminus.

Two key observations stimulated us to investigate the possibility of involvement of YbeY in sRNA regulation [16]. First, we had observed that YbeY displays high structural similarities to the MID domains of Argonaute proteins. *In silico* modeling of substrate and protein binding suggested that YbeY has the potential to bind to sRNA seeds and we identified a possible phosphate-binding center in YbeY cavity. Second, we noted striking parallels between the phenotypes of *S. meliloti hfq* and *smc01113* (*ybeY*) mutants, an observation that suggested there might be an underlying mechanistic connection. To test this hypothesis, we carried out a limited study in *S. meliloti* in which we evaluated the accumulation of 13 target genes and 9 sRNAs in *S. meliloti* compromised for their expression of Hfq or SMc01113 (YbeY). We showed that both mutants exhibited similar deregulation of sRNAs and targets [16]. This study raised the possibility that YbeY might play a role in sRNA regulation in bacteria whose importance is comparable to that of Hfq.

To test our hypothesis that YbeY plays an important role in sRNA regulation in addition to its key roles in 70S ribosome quality control and rRNA processing, we have used an integrative biology approach to evaluate the YbeY-dependent molecular response of *E. coli* cells exposed to hydroxyurea (HU), a widely used inhibitor of *E. coli*’s class I ribonucleotide reductase. Our results indicate that YbeY plays an extremely important role in bacteria, modulating the levels of both Hfq-dependent and Hfq-independent sRNAs as well as their targets.

## Results

### Design of experiment and overview of microarray analysis

**Figure 1:**
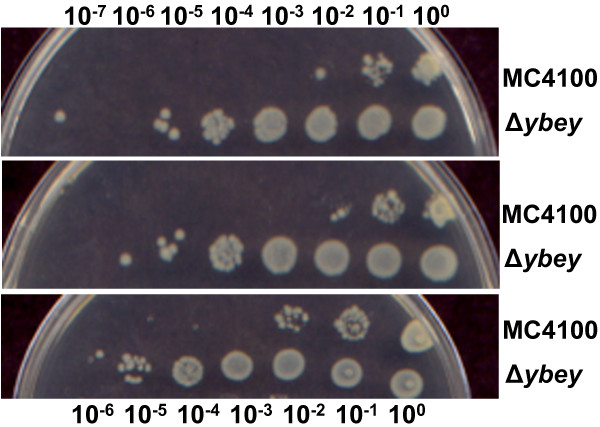
**Δ*ybeY *cells survive HU stress better than WT (MC4100).** Cell viability was assayed by 10-fold serial dilutions of cells onto plates containing 10 mM hydroxyurea. Δ*ybeY* cells displayed resistance to killing by HU as compared to WT cells.

There were two reasons for why we chose HU as the stress in our studies evaluating the role of YbeY is sRNA regulation in *E. coli.* First, we already knew that HU exposure elicits a very complex physiological response that leads to cell death and lysis and has been attributed to the production of reactive oxygen species [25]. Second, we had noted that, even though an Δ*ybeY* mutant exhibits an increased sensitivity to a wide variety of other types of stresses [16,23], it is strikingly resistant to killing by HU (Figure 1). Thus we hoped our analysis might also offer insights into the basis of this HU resistance.

**Figure 2:**
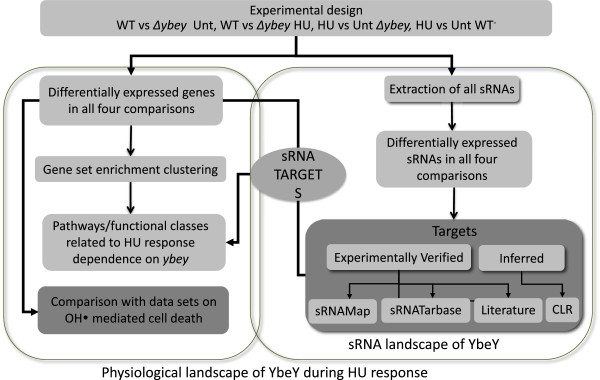
**Schematic representation of a combinatorial approach, integrating microarray analysis, gene-set enrichment analysis, database mining, literature mining and CLR, to identify YbeY-dependent sRNAs and their targets during HU-stress.** sRNAs and mRNAs differentially expressed during the conditions of presence and absence of YbeY and upon the exposure of HU was determined. sRNA-target relations were mapped to uncover the YbeY-dependent sRNA landscape in *E. coli*. Unt: untreated condition.

To study the detailed changes in molecular profiles of *E. coli* mutated for *ybeY* expression (*ΔybeY*) during exposure to HU, and to evaluate global effects of *ybeY* mutation on sRNAs and their targets, we adapted an integrative biology approach using microarray analysis, gene set enrichment clustering, database mining, literature mining and ‘context likelihood of relatedness’ (CLR; [26,27]) based network analysis (Figure 2). We generated and compared gene expression profiles of wild type (WT; MC4100) *E. coli* and its corresponding *ΔybeY* derivative in two states, untreated and HU-treated (Figure 2). As in our previous study, we examined gene expression profiles of exponentially growing WT cultures following 1 hr of treatment with or without freshly prepared 100 mM HU [25]. At this 1 hr time point, HU-treated cultures have not yet shown decreased survival but do show growth inhibition. Our hope was that we could gain insights into the early cellular events that lead to cell death and lysis by examining the expression profiles at this time during HU treatment.

**Figure 3:**
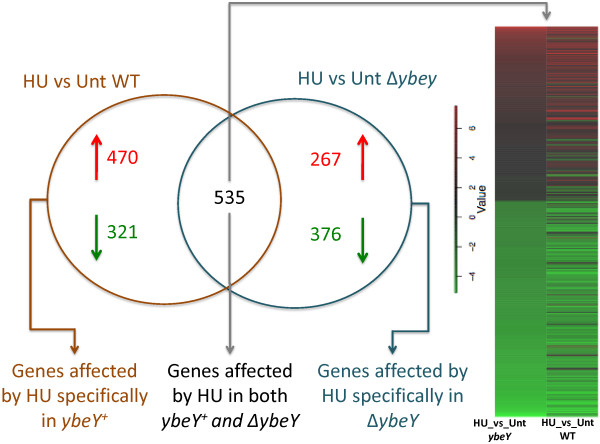
**HU-mediated differential expression of genes in WT and Δ*ybeY *genotypes.** Venn diagram shows genes that are differentially regulated when WT and Δ*ybeY* cells are exposed to HU-stress. Heat map shows the relative level of expression of genes that show a change in expression in both the genotypes upon HU treatment as compared to their respective untreated controls. Numbers in red represent genes upregulated whereas in green represent number of genes downregulated in respective conditions.

When we performed the unsupervised hierarchical clustering, we were able to distinguish WT and *ΔybeY* mutant samples at both the states (Figures 2 and 3). Equally, we were able to differentiate expression changes between untreated and HU-treated cells in both the genotypes with the help of cluster analysis (Figures 2 and 3). We have summarized the number of differentially expressed genes for all of the comparisons in Table 1. Genes showing a ΔZ-score > 1 or < -1 in expression between comparisons (see methods for details; [25]), were regarded as differentially expressed.

**Table 1:** **Summary of microarray analysis: number of differentially regulated genes between MC4100 and Δ** ***ybeY *** **with or without (Unt) HU treatment**

	**Δ** ***ybeY *** **Unt vs MC4100 Unt**	**MC4100 HU vs MC4100 Unt**	**Δ** ***ybeY *** **HU vs Δ** ***ybeY *** **Unt**	**Δ** ***ybeY *** **HU vs MC4100 HU**
Up-regulated	673	718	506	455
Down-regulated	478	608	672	625
Total	1151	1326	1178	1080

Loss of *ybeY* function results in changes in gene expression even in the absence of any stress, with 1151 genes significantly differentially expressed; 673 genes were up-regulated in the *ΔybeY* mutant relative to WT and 478 were down-regulated (Table 1, Additional file 1: Table S1). As we have previously reported [25], treatment of *E. coli* with HU causes major changes in gene expression with 1326 genes significantly differentially expressed upon HU treatment; 718 genes were significantly up-regulated upon HU treatment and 608 were significantly down-regulated (Table 1, Additional file 1: Table S1). As we have noted previously, up-regulated genes include those associated with three classes of survival responses: ribonucleotide reductases (e.g. *nrdA, nrdB, nrdD, nrdE, nrdF*), primosome components for replication restart (*priA* and *priB*), and the SOS response (e.g. *recA, recN, sulA, umuC*). We had also noted that numerous genes associated with iron import were strongly up-regulated upon treatment of WT with HU [25] (e.g. *fepC, fepD, fepG, fhuA, fhuB, fhuC, fhuD, fhuE, fhuF, tonB, exbB, exbD*)*.* Treatment of the *ΔybeY* mutant with HU also resulted in major changes in gene expression with 1178 genes significantly differentially expressed: 506 genes were up-regulated upon HU treatment and 672 were down-regulated.

A comparison of the genes whose expression was altered when the WT strain was treated with HU with those whose expression was altered when the *ΔybeY* mutant was treated with HU revealed that 535 genes displayed a significant change in expression independent of the *ybeY* status of the cells (Figure 3; Additional file 2: Table S2). Genes in this category included many of those mentioned above that are potentially associated with survival when WT is treated with HU: ribonucleotide reductases (e.g. *nrdA, nrdB, nrdD, nrdE, nrdF*) and the SOS response (response (e.g. *recA, recN, sulA, umuC*). In addition, many of the genes associated with iron import were induced by HU regardless of the *ybeY* status of the cells (e.g. *fepC, fepD, fepG, fhuE, fhuF, exbB, exbD*). Thus, the presence or absence of YbeY had only subtle affects on expression of these genes. However, many of the genes whose expression levels are altered by HU treatment are strongly influenced by the *ybeY* status of the cells. In the case of the 1326 genes whose expression was affected when the WT strain was treated with HU, the remaining 470 up-regulated genes and 321 down-regulated genes only displayed a significant change when the cells were proficient for YbeY function. Interestingly, the primosome/replication restart genes (*priA* and *priB*) were in this category, as were certain of the iron import genes (*fhuA, fhuB, fhuC, fhuD, tonB*). Reciprocally, in the case of the 1178 genes whose expression was affected when the *ΔybeY* strain was treated with HU, the remaining 267 up-regulated genes and 376 down-regulated genes only displayed a significant change when the cells lacked YbeY function. Thus the presence or absence of YbeY function is a huge factor in determining how cells respond to HU.

### YbeY dependent reprogramming of sRNA and target expression

**Figure 4:**
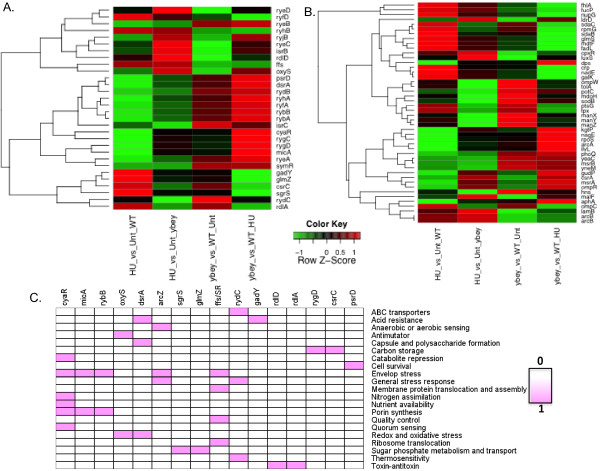
**Differential expression of sRNAs and their known targets in Δ*ybeY *and control (WT) bacteria.** Complete linkage algorithm and a euclidean distance metric were used to cluster rows and columns simultaneously to generate heatmaps. Values were transformed to zero (row-wise) mean and unit (row-wise) variance prior to clustering. The sRNA clustering tree is shown on the left **(A)** and the previously target clustering tree is shown on the right **(B)**. The color scale shown between A and B illustrates the relative expression level of the indicated sRNA and targets across all samples: red denotes expression >0 and green denotes an expression <0. sRNAs shown here are those detected on microarrays. **(C)** represents the pathways/functions of known targets of sRNAs (represented in (A) and (B).

Our pilot study of the role of the YbeY ortholog, SMc01113, in *S. meliloti*[16] suggested that YbeY plays a previously unrecognized role in sRNA-mediated regulation whose importance is comparable to that of extensively characterized Hfq. To gain insights into a possible role of *E.coli* YbeY in regulating expression of sRNAs and their targets on whole genome scale in response to HU treatment, we followed the strategy illustrated in Figure 2. Information on all the sRNAs was extracted for four comparisons: the three comparisons discussed above and also the HU-treated *ΔybeY* mutant versus HU-treated WT (Table 1). In this fourth comparison, a total of 1080 genes showed significant differences in levels of expression, with 455 genes expressed at a higher level in the HU-treated *ΔybeY* mutant than in the HU-treated WT and 625 gene expressed at a lower level. A total of 54 sRNAs were detected in our microarray analysis, of which 30 unique sRNAs showed a differential expression in at least one of the four comparisons; 28 sRNAs (>93% of differentially expressed sRNAs) showed a YbeY-dependent change in expression (Figures 2 and 4; Table 2; Additional file 3: Table S3). Cluster analysis was able to discriminate successfully the changes in expression of individual sRNAs among 4 comparisons (Figure 4). Our analysis of sRNAs suggested a complex pattern of change in expression of sRNAs when MC4100 or *ΔybeY* cells were exposed to HU. Comparison of unstressed states of *ΔybeY* and WT MC4100 cells showed up-regulation of 4 sRNAs (CyaR, RyfA, Ffs, IsrC) and down-regulation of only 1 sRNA (RdlD) in *ΔybeY*. Exposure of WT to HU changed the expression of 17 sRNAs (Table 2) of which nearly half were up-regulated (Ffs, RyhB, SgrS, GadY, CsrC, OxyS, RyfD, GlmZ) and the other half were down-regulated (RyhA, RybB, RybA, RyfA, RygC, MicA, SymR, RyeA, RygD). Exposure of *ΔybeY* to HU changed expression of 13 sRNAs (as compared to untreated *ΔybeY*), where 9 were up-regulated (IsrB, CyaR, RyeC, RyeD, RyjB, RdlD, RyhB, Ffs, OxyS) and 4 (SymR, RydC, RdlA, RyeA) were down-regulated (Table 2). On the other hand, when we compared the sRNA expression profiles of HU-treated *ΔybeY* and WT cells, 15 sRNAs (RygC, OxyS, CyaR, RyhA, RyfA, RygD, RybB, RybA, PsrD, IsrC, Ffs, RyjB, DsrA, RydB, ryeB) expressed at a higher level in *ΔybeY* whereas expression levels of only 2 (RdlA, SgrS) were lower in *ΔybeY* as compared to WT (Table 2; Figure 4). Thus, our analysis of sRNAs suggested that presence or absence of YbeY during stress accounts for major changes in sRNA expression.

**Table 2:** Number of differentially regulated sRNAs between MC4100 and ΔybeY with or without (Unt) HU treatment

	**Δ** ***ybeY *** **Unt vs MC4100 Unt**	**MC4100 HU vs MC4100 Unt**	**Δ** ***ybeY *** **HU vs Δ** ***ybeY *** **Unt**	**Δ** ***ybeY *** **HU vs MC4100 HU**
Up-regulated	4	8	9	15
Down-regulated	1	9	4	2
Total	5	17	13	17

Of the 28 differentially regulated sRNAs (detailed in above comparisons), 12 sRNAs were Hfq-dependent and 16 sRNAs were Hfq-independent. Hfq-dependent sRNAs that also showed a YbeY dependence included OxyS, DsrA, CyaR, ArcZ, RybB, MicA, GlmZ, RyeA, RyeB, CydC, RyjB and SgrS; whereas Hfq-independent YbeY dependent sRNAs are Ffs, RygD, CsrC, RdlD, RdlA, GadY, PsrD, RygC, RyfD, RyfA, RybA, RyrC, RyeD, RydB, IsrB, and IsrC.

Insights into the biological pathways regulated by YbeY-dependent sRNAs during HU treatment in *ΔybeY* and WT genotypes were obtained by identifying *bona-fide*, experimentally inferred targets and genes that are associated with differentially regulated sRNAs using two databases, sRNATarBase and sRNAMap, and by primary literature mining ([1,28]; Figures 2 and 4). 45 experimentally inferred genes associated with 11 Hfq-dependent (OxyS, DsrA, CyaR, ArcZ, RybB, MicA, GlmZ, RyeA, RyeB, CydC, and SgrS) and 6 Hfq-independent (Ffs, RygD, CsrC, RdlD, RdlA, and GadY) sRNAs were obtained. Again, cluster analysis revealed the differences in expression of individual genes between the four comparisons (Figure 4).

In *E. coli*, sRNAs can both up-regulate or down-regulate expression of their targets [3]. Therefore, for negatively regulated targets, expression levels of sRNA-mRNA target pair should be inversely correlated i.e. for a given sRNA, if its expression was up-regulated, the level of expression of its target genes were down-regulated and *vice-versa*. Similarly, for positively regulated sRNA-target pairs, levels of sRNAs as well as their targets changed in the same direction. A total of 57 sRNA-mRNA interactions for 17 sRNAs were correctly correlated to change in expression of 45 experimentally inferred genes (Figure 4), indicating that these relationships are functional YbeY-dependent-sRNA-mRNA combinations during HU response.

Combining the information on known experimentally inferred targets, obtained from sRNAMap, sRNATarbase, and primary literature, as well as clustering of targets into functional groups revealed several important pathways that most likely changed in *∆ybeY* cells when they were exposed to HU (Figure 4). For example pathways related to envelope stress, redox stress and oxidative stress, porin synthesis, translocation of membrane protein and their assembly, signal recognition particles (SRPs), anti-mutation response, metabolism, toxin-anti-toxin pairs, transporters, and cell survival (Figure 4) are altered in *ΔybeY* cells when they are exposed to HU. This analysis highlights the broad consequences of YbeY-dependent sRNA regulation on cellular physiology in response to HU stress (elaborated in the following sections).

### Inferring YbeY dependent sRNA-mRNA interactions using CLR

**Figure 5:**
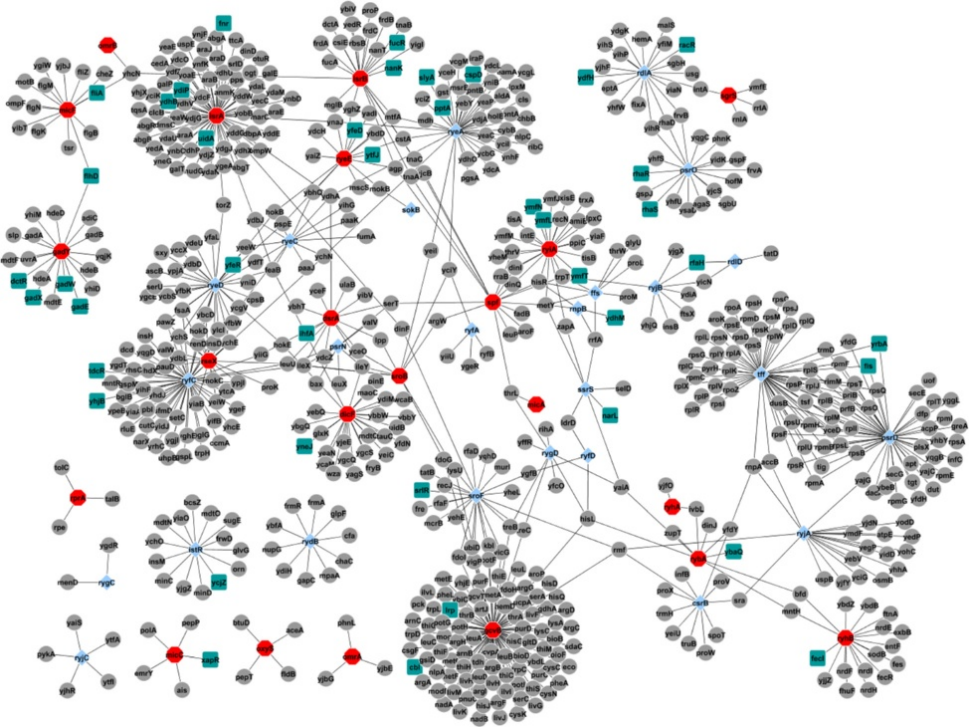
**Global sRNA-target network of *E. coli*. CLR algorithm was applied to 759 RMA-normalized expression arrays to reverse-engineer whole-genome sRNA regulatory network at an FDR corrected P value < 0.005.**

**Figure 6:**
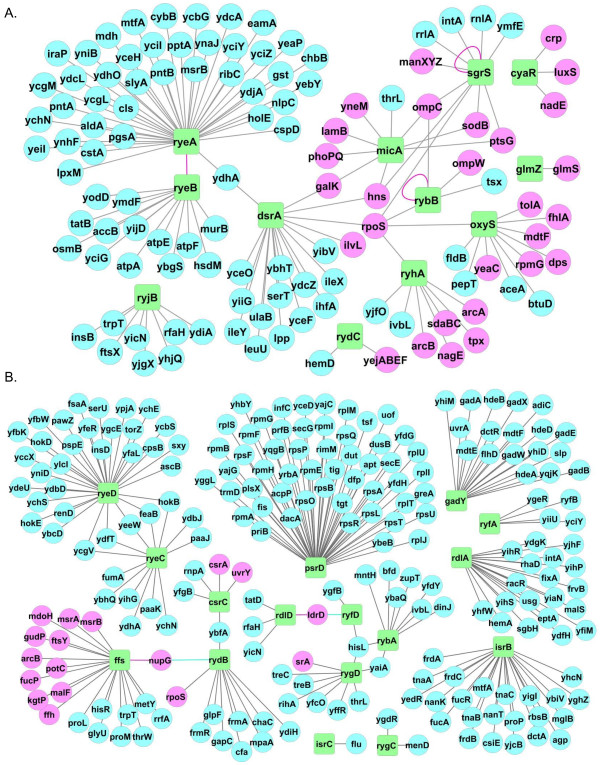
**Network of YbeY-regulated sRNAs and targets. (A)** shows the YbeY as well as Hfq-dependent sRNA target network; **(B)** represents the YbeY-dependent but Hfq-independent network respectively. Green squares are the differentially expressed sRNA in microarray analysis. Pink circles are experimentally validated targets and blue circles are CLR inferred targets that are significantly regulated in microarray analysis. Differential expression of sRNAs and target genes were determined from microarray experiments and sRNA-target interactions were determined using the strategy described in Methods and summarized Figure 2.

Identification of at least 57 sRNA-mRNA relationships that have been already experimentally inferred suggested a much wider role of YbeY in regulating sRNA-mRNA interactions. Furthermore, experimentally evaluated targets for only 17 of 28 YbeY-dependent sRNAs could be obtained from all the sources e.g. sRNATarbase, ‘a database for experimentally validated targets’ and a literature survey [28]. Therefore, in a complementary investigation, we also adapted a novel network-based systems biology approach (CLR) [26,27] to further estimate all the potential YbeY-dependent targets for the 28 differentially regulated sRNAs (Figure 4). We have recently demonstrated the use of our network-based approach in the characterization of Hfq-dependent sRNA-target relationships [27]. We applied the CLR algorithm to an existing compendium of 759 RMA-normalized *E. coli* expression arrays collected under different experimental conditions to reverse engineer and analyze the full regulatory networks for Hfq-dependent and Hfq-independent sRNAs. Using this process, we were able to infer potential targets of each of these sRNAs with a highly significant false-discovery rate (FDR)-corrected P value (q < 0.005). The inferred network (Figure 5) consists of 664 putative direct and indirect targets for the Hfq-dependent and Hfq-independent sRNAs. Based on our microarray analysis, there are 12 Hfq-dependent sRNAs and 16 Hfq-independent sRNAs showing statistical significance among four comparisons. Using these significant sRNA as “seed” nodes, we particularly identified the YbeY-dependent Hfq-dependent sRNA-mRNA sub-network (Figure 6A) as well as YbeY-dependent Hfq-independent sRNA-mRNA sub-network (Figure 6B) from the CLR reverse-engineered full sRNAs network (Figure 5). For these two sRNA-mRNA networks, we have annotated those sRNA targets as the experimentally validated targets (in pink) and putative computationally predicted targets (in blue) respectively. These Hfq-dependent and Hfq-independent sRNA-mRNA networks provide a valuable extension of our knowledge about all the sRNAs in general and YbeY-dependent sRNA and their putative targets in particular.

### Physiological responses of ∆ybeY cells to HU treatment and cell fate

Functional clustering of genes was performed to gain insight into the biological processes that potentially changed after HU-treatment in MC4100 and *∆ybeY* genotypes (Additional file 4: Table S4, Additional file 5: Table S5, Additional file 6: Table S6, Additional file 7: Table S7, Additional file 8: Table S8 and Additional file 9: Table S9). Our analysis of transcriptional reprogramming in *∆ybeY* against HU-stress suggested alteration of several pathways (Additional file 4: Table S4, Additional file 5: Table S5, Additional file 6: Table S6, Additional file 7: Table S7, Additional file 8: Table S8 and Additional file 9: Table S9) that might possibly contribute to the resistance of a *∆ybeY* mutant to HU. For example, genes contributing to envelope stress as well as those responsible for synthesis and repair of cell wall, membrane, lipoproteins and polysaccharides were strongly altered in the *∆ybeY* mutant. Similarly multiple two-component signal transduction systems that enable bacteria to sense, respond and adapt to environmental stresses were specifically regulated in the *∆ybeY* genotype*. ∆ybeY* cells adjust their TCA cycle and components of electron transfer chain in response to HU stress, alterations that could in principle contribute to a reduction in the production of harmful oxidizing radicals so that the damage to genetic material may be reduced [29]. Intriguingly, components of base excision DNA repair, which are employed by cells to repair DNA damage due to oxidizing agents, were up-regulated only in *∆ybeY* cells (Additional file 8: Table S8)*.* Furthermore, components of non-coding RNA biogenesis pathways were reprogrammed in *∆ybeY* genotype (Additional file 8: Table S8). Moreover, components of drug resistance pathways were also evidently regulated only in cells with the *∆ybeY* genotype (Additional file 8: Table S8). *∆ybeY* cells displayed several molecular characteristics similar to those undergoing adaptation to antibiotic exposure [29]. Exposure of WT to HU results in up-regulation of iron-uptake systems (Additional file 9: Table S9), which is highly detrimental to *E. coli* and since it could cause cell death during HU stress by promoting Fenton chemistry [25]. Most of these genes of iron uptake system (e.g. *tonB-exbB-exbD, fhu* system genes) were expressed at lower level in HU-exposed *∆ybeY* as compared to HU-exposed WT MC4100 (Additional file 7: Table S7). When the WT cells are exposed to HU treatment, genes like *tonB, fhuA, B, C, D* are strongly up-regulated, whereas HU-mediated up-regulation of these genes does not occur in *∆ybeY* cells. These results are consistent with our earlier observation that loss of expression of *tonB* provides a protective effect in HU-exposed cells [25].

**Figure 7:**
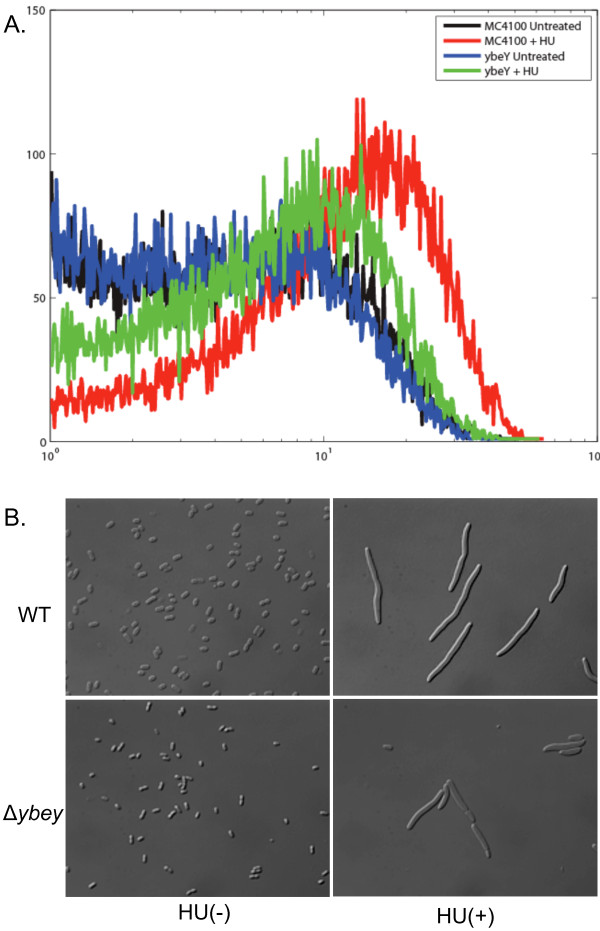
**Physiology of Δ*ybeY *as compared WT bacteria.** During HU exposure Δ*ybeY* cells have reduced production of reactive oxygen species than WT **(A)**, possibly leading to their better survival (Figure 1). FACS analysis was carried out as described in Methods. **(B)** represents microscopic states of WT and Δ*ybeY* cells during absence (HU-) and presence of HU (HU+).

Our previous work has presented evidence that the cytotoxic effect of HU treatment of WT *E. coli* leads to an oxidative response that can be detected by the oxidation to the dye 3′-(ρ-hydroxyphenyl)-fluorescein (HPF), to a fluorescent derivative [25]. Taken all together, our analyses of the of HU-induced changes of gene expression in a *∆ybeY* mutant relative to those in a WT, raised the possibility that a *∆ybeY* mutant might be resistant to killing by HU because this cytotoxic oxidative response does not occur. To test this hypothesis, we compared the oxidation of HPF in HU-treated WT cells [25] to that in HU-treated *∆ybeY* cells (Figure 7). Our results indicate that the HU treatment of the *∆ybeY* cells does not elicit the oxidation of HFP and thus that is possible that this lack of an oxidative response is the primary physiological reason that a *∆ybeY* mutant is not killed by HU. Interestingly, in the course of our experiments, we noted that both the WT and *∆ybeY* cells filamented in response to HU. This is consistent with the induction of the SOS-regulated *sulA* gene, which encodes an inhibitor of septation, in both WT and the *∆ybeY* mutant and suggests that the induction of the SOS network is a separate physiological response from the one that leads to the cytotoxic oxidative response.

## Discussion

Taken together, our results indicate that the highly conserved bacterial protein YbeY plays a major role in bacterial sRNA regulation. Extending our more limited study in *S. meliloti*[16], our analyses suggest that YbeY participates in both Hfq-dependent and Hfq-independent sRNAs-mediated interactions in *E. coli*. This means that YbeY is a remarkably central protein in RNA metabolism in bacteria, as we have additionally shown that YbeY is also involved in 70S ribosome quality control and in rRNA processing [24]. Thus YbeY can help a cell withstand stress both by modulating changes in gene expression through its role in sRNA regulation and by helping to maintain the fidelity of protein translation. YbeY’s three distinct RNA-related physiological roles offer an explanation for why it is one of 206 genes in the postulated minimal bacterial gene set [17].

We have constructed a whole-genome sRNA-target interaction network to explore organism-wide interactions for most of *E. coli* sRNAs and evaluated the subset of network that is reconfigured in YbeY-dependent manner during the response to HU. Our results suggest novel mechanistic insights into how cells respond to HU and reveals that YbeY and sRNAs play central roles. The complexity of *E.coli’*s response to HU that is evident in our experiments is fully consistent with a recent genome-wide screening study with HU that revealed a link between non-essential ribosomal proteins and reactive oxygen species [30] and the subsequent demonstration that a tRNA thiolation pathway, which modulates the intracellular redox state, affects sensitivity to HU [31].

**Figure 8:**
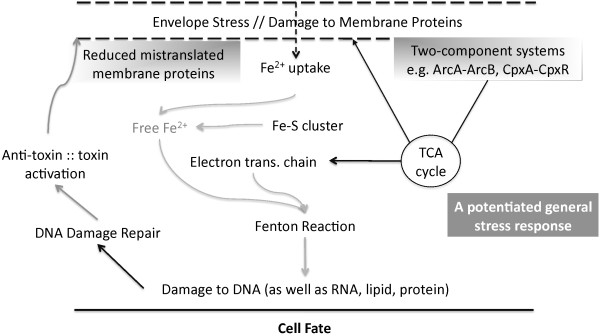
**Schematic summary of cellular signaling pathways leading to reduced reactive oxygen species production and increased fitness in Δ*ybeY *bacteria during HU exposure.** Results suggest that complex molecular changes that are mediated by YbeY action determine the cell fate during HU stress.

sRNAs mediate adaptation of bacteria to environmental fluctuations: for instance their role in quorum sensing, biofilm formation, iron uptake and virulence has been well established [3,32-34]. Yet, their involvement in cellular response to HU stress has not been addressed. A previous study from our group led us to hypothesize that exposure to HU causes cell death due to enhanced production of hydroxyl radicals that are generated as a result of increase in iron uptake, toxins, mistranslated protein and envelope stress [25]. In particular, we hypothesized that exposure to HU results in activation of cellular toxins that lead to improperly translated proteins, membrane stress, and disrupted respiratory chain activity, which causes an increase in superoxide production, eventually leading to production of excessive hydroxyl radicals [25]. However, the manner in which these processes are regulated remains largely unknown and the ultimate explanation will also needs to incorporate the recent discoveries for instance those by Mahoney and Silhavy that a cpxA^*^ mutation that constitutively activates the CpxR stress response leads confers a high level of resistance to HU [35]. Further, Kint et al. demonstrated the involvement of ObgE GTPase during hydroxyl radical toxicity and replication fork arrest [36]. Our integrative biology-guided approach suggests a central role for YbeY in which it acts by enforcing the regulation of sRNA-mediated interactions (Figures 6 and 8). Loss of YbeY resulted in up-regulation of several sRNAs (e.g. OxyS, DsrA, MicA, CyaR etc.) in response to HU that in turn affect several cellular processes central to adaptation to oxidative stresses. Further analysis of functional clusters of genes that were differentially expressed in a *∆ybeY* mutant as compared to WT suggested that exposure of a *∆ybeY* mutant to HU causes elicitation of envelope-stress responses, reprogramming of constituents of two-component systems (that are regulated by sRNAs), changes in the TCA cycle and electron transfer chain, and a reduction in iron-uptake. Together, these changes could potentially down-regulate the Fenton reaction that uses hydrogen peroxide and iron to generate free hydroxyl radicals (Figure 8). Indeed our measurements showed a striking reduction in the level of reactive oxygen species in HU-treated *∆ybeY* cells as compared to HU treated WT cells (Figure 7), an observation that can help to explain why HU is not a severe cytotoxic stress for a *∆ybeY* mutant (Figure 1). In addition, in *∆ybeY* cells, components of base excision repair, among other DNA repair pathways, were specifically up-regulated along with anti-mutation- anti-oxidative stress responses that are under the control of sRNA modules comprising of OxyS. It is well established that OxyS helps in protecting cells against oxidative damage imposed during oxidative stress by hydrogen peroxide as well as other cellular stresses [37].

Several toxin-antitoxin pairs encoded by *E. coli* genome were also differentially reprogrammed in *∆ybeY*, so was the SRP machinery (in which the Ffs sRNA is a central component) that regulates synthesis of membrane proteins and their targeting [38]. We also observed differential expression of several sRNA modules that regulate porins and outer membrane proteins (OMPs) e.g. MicA, CyaR and RybB and their targets [(Figures 4 and 6); [33,39]]. Reprogramming of SRP machinery along with sRNA-OMP modules of regulation could help to reduce the amount of mistranslated membrane proteins, repair damaged membrane proteins, and help circumventing envelope stress, thus enhancing survival (Figures 7 and 8). Our results suggest that oxidative and free radicals stresses pose a multilayered threat to cell and that YbeY dependent sRNAs (which include both Hfq- dependent as well as independent sRNAs) have hitherto unappreciated roles in helping cells to adapt to such stresses.

Although around 70 sRNAs have been estimated in *E. coli*, the targets and functions of many of them still remain unknown. We could obtain targets for only 17 differentially expressed sRNAs from literature and database sources (Figures 2 and 4). The deficiency in experimental information underscores the need to develop complementary approaches for identification of targets and functions of sRNAs as compared to standard, tedious biological and genetic protocols, and large-scale screens of mutant libraries. One such complementary approach has been recently developed in our laboratory that uses the ‘Context Likelihood of Relatedness’ (CLR) algorithm to infer the Hfq-dependent sRNA regulatory network in *E. coli*[26,27]. In our current study, we have used an *E. coli* compendium of 759 microarrays maintained in one of our laboratories [26] to expand this algorithm to all the *E. coli* sRNAs (Hfq-dependent as well as independent). We have inferred potential targets of every sRNA with a very high q-value (FDR corrected p-value; <0.005). Our results suggest presence of a complex global regulatory network modulated by sRNAs (Figures 5 and 6) that would help the bacteria to program its response effectively to changes in their environment. It is not difficult to imagine that bacteria may elicit specific smaller sub-networks and sRNA-circuits in order to adapt to specific stresses. We indeed observed a similar situation as we were able to determine the YbeY-dependent sRNA sub-network that responded to HU (Figure 6). Our analysis is also significant as it demonstrates the relevance of integrative biology approaches to infer the global and specific regulatory circuits of sRNAs, as well as presents examples of different regulatory sRNA circuits. Several models of sRNA circuitry modules have been proposed e.g. single input module, dense overlapping regulon, positive- and negative feedback modules, and feed forward modules [32]. All these modules were readily observed in our global network.

It is evident that sRNAs act post-transcriptionally and modulate gene expression through both extensive and limited base-pairing interactions with their targets. Several of these require the RNA chaperone Hfq for pairing with their targets, with Hfq assisting in the *trans*-annealing of the sRNAs to target mRNA in an antisense manner [9]. Recent progress has offered insights into how bacterial sRNAs are recognized and loaded on to the Hfq protein scaffold during their interaction with the target mRNAs [12-14]. However, Hfq has not been identified in numerous sequenced bacteria, whereas YbeY is extremely highly conserved. Thus, our results raise the possibility that YbeY may play an especially important role in sRNA regulation in bacteria that do not encode Hfq.

The phenomenon of sRNA recognition and its guidance to target mRNA is quite well understood in higher organisms, where miRNAs and siRNAs are loaded onto Argonaute proteins and then guided and assembled on to the targets by the RISC. Structures of Argonaute proteins include a ‘MID domain’ that specifically recognize 5′-phosphate of the sRNAs and anchors them on to the Argonaute/RISC, and a ‘PIWI domain’ that have hydrolytic (RNase) capacity [15]. Interestingly, it was our observation that YbeY has structural similarities to the MID domain of Argonaute proteins that stimulated us to consider the possibility that YbeY might play a role in bacterial sRNA regulation and to carry out modeling studies indicating that it had the potential to bind a small seed RNA.

In contrast, considerably less is known about how bacterial sRNAs are recognized to interact with their targets. Although important recent work has shown that a 5′-monophosphorylated sRNA seed both guides RNase E to its mRNA target and stimulates degradation, and that Hfq is needed for optimal RNase E activity in this sRNA-guided mRNA cleavage [11], many mechanistic questions remain. Furthermore, as noted above, many bacteria lack Hfq yet exhibit sRNA regulation, while our results suggest that YbeY-dependent, Hfq-independent sRNA regulation may be considerably more important in bacteria that possess Hfq than has hitherto been recognized.

It will be extremely interesting to determine how YbeY participates mechanistically in sRNA regulation. YbeY possesses a metal-dependent, single strand endoribonuclease activity that is relatively weak compared to many strictly degradative RNases [24]. Furthermore, as its RNase activity on naked RNA substrates is not particularly specific (a preference for cleavage after U’s), yet its in vivo activities in rRNA processing are highly specific, its RNase activity within living cells must be highly controlled [24].

A particular biochemical characteristic of YbeY that is potentially of interest with respect to its possible roles in sRNA regulation is that YbeY can bind to and cut single stranded oligoribonucleotides as short as 10 nucleotides in length, but is not able to cut a 7 base oligoribonucleotide despite the presence of a site that is cleaved in the context of larger oligoribonucleotides [24]. This raises the possibility that YbeY could play two possible mechanistic roles in sRNA regulation in bacteria. A possible non-catalytic role of YbeY could be that it binds a seed RNA and subsequently influences the interaction of that seed with its target mRNA or with other proteins. A possible catalytic role of YbeY could be that it participates in the degradation of the target mRNA and/or the sRNA.

A particular structural characteristic of YbeY is also of potential interest with respect to its possible roles in sRNA regulation. Both the MID domain of AGO and RNase E have a seed-binding site that is constrained at the 5′-end of the seed RNA and, in fact, both particularly recognize the 5′-phosphate of the RNA seed [11,15]. In contrast, the RNA binding site of YbeY is an open channel [16]. Although our modeling studies suggest that YbeY could bind a short seed RNA and even recognize a 5′-phosphate, the nature of the YbeY structure suggests that it could potentially interact with a seed sequence that is internal to the sRNA. Since some seed sequences of sRNA are internal and thus are not expected to end with a 5′-phosphate [3,39], YbeY might possibly play a particularly important role in their regulatory action.

## Conclusion

Taken together, our study places YbeY in the centre of sRNA-mediated gene regulation in bacterial genomes. Our study also offers mechanistic insights into regulatory basis of response of *E. coli* to HU stress. Along with demonstrating the role of YbeY, this study places sRNA pathway at the center of cellular response to oxidative stress caused by exposure of cells to HU.

## Methods

### Strains, growth conditions, treatment and microarrays

All strains were grown in Luria-Berani (LB) medium at 37°C with constant aeration. Strains were grown with or without 100 mM HU in liquid cultures for microarrays and with 10 mM HU on LB-Agar plates for survival assays [25]. The effect of *ΔybeY* mutation on growth rate is very modest and does not affect the cell density that is achieved [25]. For isolation of RNA, MC4100 and *∆ybeY* were grown with or without HU as described previously [25]. Three independent cultures were grown for isolating RNA as previously described; cDNA was prepared and microarray were performed for three independent biological replicates as described [25].

*.CEL files obtained from microarray hybridizations were combined with those in the ‘*E. coli* CDS compendium’ regularly maintained in J.J. Collins’ lab [26]. The backbone of this compendium is the publically available M3D database at http://m3d.mssm.edu/[40]. The raw intensities were background adjusted, log_2_-transformed and RMA-normalized with RMAexpress. The *E. coli* compendium used here comprised of a total of 759 RMA-normalized *E. coli* expression arrays, which are also publically available at the M3D database. Standard deviation (SD) of expression, σ, was calculated across the entire compendium for each gene.

We used the ‘z scale difference’ statistic described previously [25,29] and defined: ΔZ = [(Xt – Xc)/σ], where Xt and Xc are the normalized gene expression values for a give gene in treatment and control arrays respectively. ΔZ values were calculated for all the four comparisons, WT MC4100 HU treated vs. untreated, *ΔybeY* HU treated vs. untreated, *ΔybeY* HU treated vs. WT MC4100 HU treated, and *ΔybeY* untreated vs. WT MC4100 untreated (Figure 2). Genes with a |ΔZ| score of >1 was considered significant [25,29]. ΔZ allows the measurement of change in expression of each gene in any given comparison in ‘SD units’ in form of a z-test [41]. The SD of the z-score standardization allows comparison of each observation from different normal distributions, and the average of zero avoids introducing aggregation distortions stemming from differences in the normal gene expression means. Thus, genes with extreme expression values will have intrinsically greater effects on the composite standard z-scores.

### Functional clustering of genes, identification of sRNA and their targets, and network maps

Clustering of gene sets was performed using the Database for Annotation, Visualization and Integrated Discovery (DAVID; http://david.abcc.ncifcrf.gov/; [42]) to identify potential gene pathways and key functional groups that may modulate response of bacteria to HU or knock out of *ybeY* (Figure 1). Curated gene sets from KEGG pathways, Swiss-Prot (SP) and Protein Information Resource (SP PIR keywords), Uniprot sequence features (UP Seq Feature), COG (clusters of orthologous groups) ontology, GO term analysis and SMART (simple modular architectural tool) that includes well-studied metabolic and signaling pathways, were used for annotation and clustering of genes into functional groups. Categories of function in the differentially expressed genes were determined for both up- and down-regulated genes.

For evaluating sRNAs, names of all the sRNAs were extracted from sRNAMap database [1]. This list of sRNAs was mapped onto the microarray annotation file and all the information was extracted for the sRNAs that were found on *E. coli* microarray. The nomenclature of *E. coli* sRNAs is still not standardized and many sRNAs are known by alternate names e.g. 4.5S sRNA is also called Ffs sRNA. Therefore, we performed literature mining for all the remaining sRNAs that could not be found on the *E. coli* expression array in our first round of matching; sRNAs with alternate names were remapped to microarrays. Expression of a total of 54 sRNAs was detectable. Level of significance among four comparisons was determined as described above.

For the list of differentially regulated sRNAs in any one of the four comparisons, experimentally inferred information related to targets was extracted from sRNAMap and sRNATarbase databases [1,28]. In parallel, we mined primary literature to extract such information about their targets and functions of all the differentially expressed sRNAs (Figure 2). Similarly, classification of sRNAs into Hfq- dependent and independent categories was based on primary literature and information in these databases. Functional categories for experimentally inferred targets as well as pathways in which these sRNAs may act were determined as described above.

In complementation to the above approach, we adapted a recently developed CLR based strategy to evaluate network of sRNA targets for all the sRNAs and extracted sub-network that responded to HU in YbeY dependent manner. CLR algorithm is based on relevance network theory, infers cellular regulatory interactions from a compendium of expression profiles (the algorithm is available at http://m3d.mssm.edu/network_inference.html). Although CLR was originally designed to identify regulatory interactions of transcription factors, in this work we adapt the algorithm to examine sRNA regulatory influences because of their role as posttranscriptional regulators of mRNA stability. An sRNA and a gene are predicted to interact if the mutual information between their expression levels is above a set threshold. Mutual information is a measure of the statistical dependence between two variables and, in contrast to correlation, does not assume linearity, continuity, or other specific properties of the dependence. CLR computes the significance of mutual information by assembling a background distribution from the mutual information scores of all other microarray probe sets in the compendium. This adaptive background correction allows the algorithm to eliminate false correlations and indirect functional influences. sRNA–gene interactions found to be significant using this procedure are represented in the network diagram as edges between nodes. Network analysis presented in this work focuses on the regulatory influences of Hfq-dependent sRNAs. At the time this work began, 27 Hfq-dependent sRNAs had been identified in *E. coli*. Because of the nature of Affymetrix annotation, our methodology is restricted to inferring relationships for genes associated with a Blattner ID, constraining our network to 24 Hfq-dependent sRNAs. We used a compendium of 759 Affymetrix *E. coli* Antisense2 arrays uniformly normalized with RMA to serve as input to the algorithm. This compendium includes arrays from the Many Microbe Microarray Database (*E_coli*_v3_Build_3), as well as other 235 arrays run in-house. Experiments did not include genetic or environmental perturbations specifically related to sRNAs but were generally focused on bacterial stress response. The reconstructed sRNA-mRNA regulatory network can help us to gain insights into the functional roles of these sRNAs. All computations were run in Matlab (Mathworks).

### Hydroxyurea sensitivity assays, measurement of reactive oxygen species, and microscopy

For determining the effect of knocking out *ybeY* on the survival of *E. coli*, we conducted spot test assays on LB-Agar plates containing 10 mM hydroxyurea. Overnight grown WT MC4100 and *ΔybeY* cultures were diluted 1:1000 (OD_600_ of ~0.01) and grown to OD_600_ 0.5-0.6; these were serially diluted and 5 μl of each dilution was spotted on LB-Agar-HU plates. Plates were incubated at 37°C overnight. For measurement of reactive oxygen species and microscopy, cells were grown to early exponential phase and then treated with 100 mM HU. 100 μL samples were collected hourly, centrifuged at 10,000 rpm, and resuspended in 100 μL PBS + 5 mM 3′-(p-hydroxyphenyl fluorescein (HPF). Cells were incubated in the dark for 15 minutes at room temperature, then centrifuged at 10,000 rpm and resuspended in 1X PBS for microscopy and FACS analysis. For FACS analysis, fluorescence data were collected using a Becton Dickinson FACSCalibur flow cytometer. For microscopy, images were obtained using the 100X oil-immersion objective lens.

## Competing interests

The authors declare that they have no competing interests.

## Authors’ contributions

SPP conceived study, SPP and GCW designed study, SPP, JAW, HL, DMC conducted study and analyzed data, JJC, GCW provided resources, SPP, JAW, HL, JCC and GCW wrote the manuscript. All authors read and approved the manuscript.

## Supplementary Material

Additional file 1: Table S1 Differentially expressed genes and their ΔZ scores in four combinations as summarized in Table 1. Click here for file

Additional file 2: Table S2 Names and ΔZ scores of genes changing during exposure to HU only in *ybeY* + or *ybeY*- condition as shown in Figure 2 (columns A-E). Further, names and ΔZ scores of 535 genes overlapping in Figure 2 are illustrated in G-J. Click here for file

Additional file 3: Table S3 ΔZ scores for all the small-RNAs. Scores ≥ |1| are significantly regulated. Click here for file

Additional file 4: Table S4 Functional clusters of genes differentially up-regulated in Δ*ybeY* vs. WT under unstressed state. Click here for file

Additional file 5: Table S5 Functional clusters of genes differentially down-regulated in *ΔybeY* vs. WT under unstressed state. Click here for file

Additional file 6: Table S6 Functional clusters of genes expressing higher in *ΔybeY* vs. WT under HU. Click here for file

Additional file 7: Table S7 Functional clusters of genes showing significantly lower expression in *ΔybeY* vs. WT under HU. Click here for file

Additional file 8: Table S8 Functional clusters of genes differentially regulated upon HU exposure only in YbeY’s absence. Click here for file

Additional file 9: Table S9 Functional clusters of genes differentially regulated upon HU exposure only in YbeY presence (Figure 2). Click here for file
