# Supplementary material for: Central role for RNase YbeY in Hfq-dependent and Hfq-independent small-RNA regulation in bacteria
Source: BMC Genomics. 2014 Feb 11;15:121. doi: 10.1186/1471-2164-15-121 (PMC3933206; doi:10.1186/1471-2164-15-121)
Supplement: Additional file 4: Table S4 — Functional clusters of genes differentially up-regulated in ΔybeY vs. WT under unstressed state. [file 1471-2164-15-121-S4.pdf]

Supplemental Table 4. Functional clusters of genes differentially up-regulated in *ΔybeY* vs. WT under unstressed state

Annotation Cluster 1	Enrichment Score: 36.31768336124915	
Category	Term	Genes
SP_PIR_KEYWORDS	membrane	YDJZ, YDJX, FSR, TRKG, YDJM, YNAI, EAMA, YNAJ, YBBM, PHOQ, ABRB, YIAV, YDJE, YOAE, ACRE, YBBY, GNTP, BCR, AQPZ, YICL, CAIT, ATPI, RHTA, YBCI, PLDA, HTRE, RFBX, YMBA, GFCE, GFCD, SFMD, YCHE, YCHM, YCIS, YAAH, SAPF, YCIB, YCIC, SAPD, SAPC, YOBD, ANSP, YCFZ, FRDD, MSBA, YAFT, MDTC, SSUB, MDTA, MDTB, YBFP, MDTH, YBFN, MDTK, MDTI, MDTJ, YMFA, CLD, TYRP, TORY, GFCA, YDGC, LOLB, MODC, YBGQ, TEHA, LOLE, YEHW, YDGK, RBBA, YIJP, YDHP, APBE, DJLA, YNJC, YDHU, FTSX, LPXM, YDHC, SBMA, YNJF, AAEB, UIDC, NFRB, YNJI, NFRA, YDHK, YDHJ, YBHT, YIDC, YQFA, YBIO, MLTF, YEJB, YEJM, EXUT, CMTA, SDHC, BAES, MLTD, POTB, YBIP, POTA, YHDU, YBIR, POTC, YFIN, PITA, YEER, MRCB, MRCA, SPR, YCBS, YEDV, HOFC, YEDQ, YAJR, RFC, WBBK, YNIB, YBJE, HOFQ, YEDE, MRDA, MRDB, YEDA, YBJO, RSXC, PGSA, YDCO, YCCS, YAJI, MDOC, YDDG, YLIF, FDRA, YNFA, KDPD, TATB, COBS, YNFE, YNFF, WAAU, YNFH, YDDW, YEGH, UHPB, UHPC, MDOH, WAAA, YJIK, SETA, CMR, YDEE, TORC, TAUC, EMRD, TAUB, TAUD, YCAD, YCAI, PHEP, YCAM, OMPW, LEUE, FLU, CCMG, YTFT, CCMH, CCMF, YEAJ, YKGB, DCUB, YEAN, PRC, TQSA, PGPB, ETK, YNEF, YFDV, MDLA, MDLB, YNEN, YNEM, SPPA, TOLA, AMID, TOLR, YNBB, YECN, RTN, PGAA, PGAD, YFCA, SDAC, FADK, DMSD, YFBV, DMSC, MENA, YEBO, YBAL, YEBT, YEBS, YGJV, ZNUB, ZNUC, YEAV, YEAY, HFLD, GPT, KEFA, YHJV
Annotation Cluster 2	Enrichment Score: 10.89394787982058	
Category	Term	Genes
KEGG_PATHWAY	ecr02020:Two-component system	TORA, NARP, FRDD, DCUB, CITD, TORC, KDPD, CITG, SDIA, MDTC, UHPB, UHPA, MDTA, BAES, AMPC, UHPC, MDTB, PHOQ, CITX, PHOP, FLHC, FLHD
Annotation Cluster 3	Enrichment Score: 7.784671846867312	
Category	Term	Genes
SP_PIR_KEYWORDS	ATP	DNAA, MSBA, ASPS, ASNS, GYRB, YNJD, REP, SSUB, MFD, FADK, YCBS, TMK, HOFB, UVRD, GLTX, MDLA, BIRA, TYRS, MDLB, RECG, UUP, CAIC, YCEQ, ARGS, SAPF, ZNUC, TDK, SAPD, MODC, PRIA, POTA
SP_PIR_KEYWORDS	nucleotide binding	DNAA, YNBB, MSBA, COBU, KDPD, YNJD, REP, SSUB, MFD, NFRA, YCHF, YCBS, TMK, HOFB, UVRD, MDLA, MDLB, TAUB, RECG, UUP, YCEQ, SAPF, ZNUC, TDK, SAPD, TOLA, MODC, CITX, PRIA, POTA
GOTERM_MF_FAT	GO:0016887~ATPase activity	RBBA, MSBA, COBT, COBS, RFBB, YBHA, YNJD, REP, SBMA, YOAA, SSUB, MFD, RECQ, TBPA, YCBZ, UVRD, MDLA, MDLB, TAUB, RECG, LHR, UUP, ZNUB, SAPF, ZNUC, SAPD, MODC, YEHX, MODE, PRIA, POTA
INTERPRO	IPR017871:ABC transporter, conserved site	RBBA, MSBA, MDLA, MDLB, TAUB, RFBB, UUP, YNJD, SAPF, ZNUC, SAPD, SSUB, MODC, YEHX, POTA
UP_SEQ_FEATURE	domain:ABC transporter	SAPF, ZNUC, SAPD, MSBA, SSUB, MDLA, MDLB, TAUB, YEHX, MODC, POTA, YNJD

INTERPRO	IPR003439:ABC transporter-like	RBBA, MSBA, MDLA, MDLB, TAUB, RFBB, UUP, YNJD, SAPF, ZNUC, SAPD, SSUB, MODC, YEHX, POTA
UP_SEQ_FEATURE	nucleotide phosphate-binding region:ATP	TTCA, DNAA, MSBA, COBU, YNJD, REP, GSPA, YOAA, SSUB, MFD, PHOQ, RECQ, FADK, TMK, HOFB, UVRD, MNMA, CMK, MDLA, TAUB, MDLB, RECG, LHR, PDXY, PRPR, SAPF, ZNUC, PYRH, TDK, SAPD, ISPE, COAA, MODC, YEHX, PRIA, POTA, UDK, RHO
Annotation Cluster 4	Enrichment Score: 7.166008866141713	
Category	Term	Genes
SP_PIR_KEYWORDS	transport protein	GFCE, CMR, MDLA, MDLB, UUP, ZNUB, SAPF, ZNUC, SSUB, POTB, POTA, YCHM, YCBS, POTD, POTC
KEGG_PATHWAY	ecd02010:ABC transporters	YEJB, MSBA, TAUC, MDLB, TAUB, SAPA, ZNUB, FTSX, SAPF, ZNUC, YGIS, SAPD, MPPA, SSUB, SAPC, MODC, YEHX, POTB, POTA, LOLE, YEHW, POTD, POTC
Annotation Cluster 5	Enrichment Score: 6.380048826103632	
Category	Term	Genes
SP_PIR_KEYWORDS	nucleotide-binding	NARP, ASPS, MSBA, PRS, CITG, PRPE, YEAP, GSPA, YOAA, SSUB, MFD, PHOQ, RECQ, GLXK, YEDV, ETK, YKFA, TMK, TOPA, HOFB, TOPB, UVRD, GLTX, MNMA, THIK, TYRS, BIRA, MDLA, MDLB, RECG, YBCF, UUP, PDXY, ARGS, PYRH, MODC, YEHX, TTCA, RBBA, USHA, DNAA, YADB, KDPD, COBU, NDK, ASNS, GYRB, HSCC, RFBB, YNJD, REP, UHPB, RFAY, YCHF, FADK, POXA, MENE, NADD, CMK, TAUB, YEJH, LHR, PRPR, SAPF, ZNUC, SAPD, ISPE, TDK, BAES, FOLK, COAA, PCNB, PRIA, POTA, YFJP, RHO, UDK
SP_PIR_KEYWORDS	atp-binding	NARP, ASPS, MSBA, PRS, CITG, PRPE, GSPA, YOAA, SSUB, MFD, PHOQ, RECQ, GLXK, YEDV, ETK, TMK, TOPA, HOFB, TOPB, UVRD, GLTX, MNMA, THIK, TYRS, BIRA, MDLA, MDLB, RECG, YBCF, UUP, PDXY, ARGS, PYRH, MODC, YEHX, TTCA, RBBA, DNAA, YADB, KDPD, COBU, NDK, ASNS, GYRB, HSCC, RFBB, YNJD, REP, UHPB, RFAY, FADK, POXA, MENE, NADD, CMK, TAUB, YEJH, LHR, PRPR, SAPF, ZNUC, SAPD, ISPE, TDK, BAES, YFAL, FOLK, COAA, PCNB, PRIA, POTA, RHO, UDK
GOTERM_MF_FAT	GO:0005524~ATP binding	NARP, ASPS, MSBA, PRS, CITG, PRPE, GSPA, YOAA, SSUB, MFD, PHOQ, RECQ, GLXK, YEDV, ETK, TMK, TOPA, HOFB, TOPB, UVRD, GLTX, MNMA, THIK, TYRS, MDLA, BIRA, MDLB, RECG, QUEC, YBCF, UUP, PDXY, ARGS, PYRH, EUTJ, MODC, YEHX, MODE, TTCA, RBBA, DNAA, YADB, KDPD, COBU, NDK, ASNS, GYRB, HSCC, RFBB, YBHA, YNJD, REP, SBMA, UHPB, NFRB, YEGI, FADK, TBPA, POXA, MENE, NADD, CMK, TAUB, YEJH, LHR, ZNUB, PRPR, SAPF, ZNUC, SAPD, ISPE, TDK, BAES, YFAL, FOLK, COAA, PCNB, INAA, PRIA, POTA, RHO, UDK
GOTERM_MF_FAT	GO:0000166~nucleotide binding	ASPS, YOAA, MFD, UGD, PHOQ, RECQ, YEDV, TMK, TOPA, HOFB, TOPB, UVRD, GLTX, MNMA, THIK, QUEC, RECG, YBCF, PDXY, ARGS, EUTJ, RSXC, TTCA, DNAA, COBU, KDPD, GYRB, UHPB, YEGI, YCHF, POXA, CMK, TAUB, LHR, PRPR, SAPF, SAPD, TDK, ISPE, YFAL, FOLK, COAA, PCNB, PRIA, INAA, RHO, UDK, NARP, MSBA, PRS, CITG, PRPE, YEAP, GSPA, HEMA, SSUB, GLXK, NORV, ETK, YKFA, BIRA, MDLA, TYRS, MDLB, UUP, PYRH, YEHX, MODC, MODE, USHA, RBBA, YADB, NDK, ASNS, DUSC, HSCC, RFBB, YBHA, YNJD, REP, SBMA, NFRB, FADK, YDIR, TBPA, MENE, NADD, YEJH, MIOC, ZNUB, FOLA, ZNUC, FIXB, DUSA, BAES, POTA, YFJP

Annotation Cluster 6	Enrichment Score: 5.6184365503727856	
Category	Term	Genes
SP_PIR_KEYWORDS	lipopolysaccharide biosynthesis	RFAG, YIJP, WBBI, WCAC, WBBJ, RFBA, RFBB, CLD, RFC, RFBC, WBBK, RFBD, WAAU, LPXM, RFAY, GLF, WAAA, RFBX, ARND
GOTERM_MF_FAT	GO:0042280~cell surface antigen activity, host-interacting	RFAG, RFBA, RFC, CLD, RFBB, WAAU, LPXM, RFAY, UGD, ASMA, GLF, WAAA, RFBX, LPXH
GOTERM_BP_FAT	GO:0000271~polysaccharide biosynthetic process	MDOC, MDOG, RFBA, RFBB, RFBC, RFBD, WAAU, HIPA, HIPB, MRCB, LPXM, RFAY, PRC, MRCA, UGD, MDOH, WAAA, LPXH, RFAG, ETK, YEDQ, WCAC, WBBI, WBBJ, ALR, CLD, RFC, WBBK, MRDA, AMPC, ASMA, GLF, AMIC, INAA, RFBX
GOTERM_BP_FAT	GO:0016051~carbohydrate biosynthetic process	MDOC, MDOG, RFBA, RFBB, RFBC, HIPA, WAAU, RFBD, HIPB, MRCB, LPXM, NAGC, RFAY, PRC, MRCA, UGD, MDOH, SDAA, WAAA, LPXH, RFAG, ETK, YEDQ, WCAC, WBBI, WBBJ, ALR, CLD, RFC, WBBK, MRDA, AMPC, ASMA, GLF, AMIC, INAA, RFBX
GOTERM_BP_FAT	GO:0005976~polysaccharide metabolic process	MDOC, MDOG, RFBA, RFBB, RFBC, HIPA, WAAU, RFBD, HIPB, MRCB, LPXM, RFAY, PRC, MRCA, UGD, MDOH, WAAA, LPXH, RFAG, ETK, YIEL, ARRD, YEDQ, WCAC, WBBI, WBBJ, ALR, CLD, RFC, YAGH, WBBK, MRDA, MPPA, AMPC, ASMA, AMID, GLF, AMIC, INAA, RFBX

Annotation Cluster 7	Enrichment Score: 5.48186976177847	
Category	Term	Genes
SP_PIR_KEYWORDS	dna-binding	YCJW, FIS, NARP, YFER, CSPH, CHPS, CYNR, QUUQ, YEAT, CSPF, RSSB, ICLR, YEAM, CSPB, YPHH, YOAA, MATA, MFD, SRLR, RECQ, PHOP, XAPR, FLHC, RECT, YDJF, YDFH, FLHD, YEDW, TOPA, TOPB, UVRD, BIRA, RECG, RACR, YDCQ, DICA, GUTM, CHPB, MODE, YDCN, YBJK, PURR, DNAA, YNFL, YAIW, DEOR, ENVY, YBHD, HIPA, HIPB, REP, SDIA, YDHB, KDGR, UHPA, NAGC, ULAR, LEUO, LSRR, INSH, DGOR, YDIP, UIDR, YBAO, RUTR, RPIR, GALS, ABGR, YHJC, PRPR, YFJR, PERR, ILVY, PRIA, QUUD
SP_PIR_KEYWORDS	Transcription	YCJW, FIS, NARP, YFER, CSPH, CYNR, QUUQ, YEAT, CSPF, RSSB, ICLR, YEAM, CSPB, MATA, MFD, SRLR, PHOP, XAPR, FLHC, YDJF, YDFH, FLHD, YEDW, BIRA, FIME, YDCQ, RACR, DICA, GUTM, MODE, YDCN, YBJK, PURR, YNFL, DEOR, ENVY, YBHD, HIPB, SDIA, YDHB, KDGR, UHPA, NAGC, ULAR, LEUO, LSRR, DGOR, YDIP, UIDR, YBAO, RUTR, RPIR, GALS, YBAK, ABGR, YHJC, PRPR, YFJR, PCNB, PERR, ILVY, QUUD, RHO
SP_PIR_KEYWORDS	transcription regulation	YCJW, FIS, NARP, YFER, CSPH, CYNR, QUUQ, YEAT, CSPF, RSSB, ICLR, YEAM, CSPB, MATA, SRLR, PHOP, XAPR, FLHC, YDJF, YDFH, FLHD, YEDW, BIRA, FIME, YDCQ, RACR, DICA, GUTM, MODE, YDCN, YBJK, PURR, YNFL, DEOR, ENVY, YBHD, HIPB, SDIA, YDHB, KDGR, UHPA, NAGC, ULAR, LEUO, LSRR, DGOR, YDIP, UIDR, YBAO, RUTR, RPIR, GALS, YBAK, ABGR, YHJC, PRPR, YFJR, PERR, ILVY, MLTD, QUUD, RHO

GOTERM_MF_FAT	GO:0003677~DNA binding	YCJW, FIS, CHPS, CYNR, QUUQ, ICLR, INTD, YOAA, YPHH, YCJD, MATA, YNAK, MFD, SRLR, RECQ, PHOP, XAPR, RECT, FLHC, YDJF, FLHD, YEDW, TOPA, TOPB, UVRC, UVRD, RECG, RACR, YDCQ, LIGA, INTR, GUTM, CHPB, INTQ, YDCN, YBJK, PURR, YNFL, DNAA, YAIW, DEOR, GYRB, HIPA, HIPB, KDGR, YBDM, NAGC, UHPA, YEGI, ULAR, DNAN, LEUO, LSRR, INSH, DGOR, YCIV, RUTR, VSR, GALS, PRPR, PRIA, QUUD, ALKA, NARP, YFER, CSPH, XTHA, YEAT, CSPF, RSSB, YEAM, CSPB, HOLB, HOLA, YDFH, BIRA, FIME, DICA, YIIE, MODE, DCM, ENVY, YBHD, REP, SDIA, YDHB, YBAV, UIDR, YDIP, YNCI, YBAO, RPIR, YEJH, HOLE, ABGR, YHJC, YFJR, PERR, ILVY

Annotation Cluster 8	Enrichment Score: 4.2100439367030935	
Category	Term	Genes
KEGG_PATHWAY	ecc00240:Pyrimidine metabolism	TMK, USHA, PYRF, PYRH, TDK, HOLB, HOLA, CMK, NDK, HOLE, DNAN, UDK
KEGG_PATHWAY	eck03430:Mismatch repair	UVRD, HOLB, HOLA, LIGA, HOLE, EXOX, DNAN
GOTERM_BP_FAT	GO:0006261~DNA-dependent DNA replication	TOPA, DNAA, TOPB, UVRD, GYRB, MIOC, HOLE, LHR, RECG, REP, HOLB, HOLA, RECQ, PRIA, DNAN
KEGG_PATHWAY	ecd03440:Homologous recombination	HOLB, HOLA, RUVC, RECG, HOLE, PRIA, DNAN
KEGG_PATHWAY	ecc03440:Homologous recombination	HOLB, HOLA, RUVC, RECG, HOLE, PRIA, DNAN
KEGG_PATHWAY	eum00230:Purine metabolism	USHA, HOLB, HOLA, NDK, GPT, HOLE, YBCF, PURB, ALLD, DNAN
SP_PIR_KEYWORDS	dna replication	REP, TOPA, DNAA, UVRD, HOLB, HOLA, LIGA, HOLE, PRIA, DNAN
GOTERM_BP_FAT	GO:0006260~DNA replication	TOPA, DNAA, TOPB, UVRD, YCIV, GYRB, RECG, LHR, HOLE, MIOC, REP, TDK, HOLB, HOLA, LIGA, RECQ, PRIA, DNAN
GOTERM_MF_FAT	GO:0016779~nucleotidyltransferase activity	NADD, YNBB, YCIV, COBU, RFBA, HOLE, REP, HOLB, HOLA, PCNB, CITX, FLHC, DNAN
GOTERM_MF_FAT	GO:0003887~DNA-directed DNA polymerase activity	REP, YCIV, HOLB, HOLA, HOLE, DNAN
GOTERM_MF_FAT	GO:0034061~DNA polymerase activity	REP, YCIV, HOLB, HOLA, HOLE, DNAN
KEGG_PATHWAY	ecl00230:Purine metabolism	USHA, HOLA, NDK
KEGG_PATHWAY	ecy00230:Purine metabolism	USHA, HOLA, NDK
KEGG_PATHWAY	ecp00230:Purine metabolism	USHA, HOLA, NDK

KEGG_PATHWAY	ecs00230:Purine metabolism	USHA, HOLA, NDK

Annotation Cluster 9	Enrichment Score: 4.165570140627555	
Category	Term	Genes
GOTERM_BP_FAT	GO:0042493~response to drug	CMR, BCR, FSR, EMRD, YAJR, MDLA, AMPH, MRCB, HEMA, SBMA, MRDA, YBBM, PRC, AMPC, MRCA, MDTK, TEHA
SP_PIR_KEYWORDS	antibiotic resistance	BCR, FSR, GYRB, MRCB, FOLA, RLMN, MRDA, AMPC, MRCA, MDTH, TEHA, MDTK, ARND
GOTERM_BP_FAT	GO:0046677~response to antibiotic	MRCB, FOLA, RLMN, BCR, MRDA, FSR, AMPC, MRCA, MDTH, GYRB, MDTK, TEHA

Annotation Cluster 10	Enrichment Score: 3.9608078012294987	
Category	Term	Genes
GOTERM_BP_FAT	GO:0009243~O antigen biosynthetic process	GLF, RFBA, RFBX, RFBB, CLD, RFC, RFBC, RFBD
KEGG_PATHWAY	ecd00523:Polyketide sugar unit biosynthesis	RFBA, RFBB, RFBC, RFBD
GOTERM_BP_FAT	GO:0009226~nucleotide-sugar biosynthetic process	USHA, RFBA, RFBB, RFBC, RFBD
KEGG_PATHWAY	eci00521:Streptomycin biosynthesis	RFBA, RFBB, RFBC, RFBD

Annotation Cluster 11	Enrichment Score: 3.806171257151025	
Category	Term	Genes
SP_PIR_KEYWORDS	protein transport	TOLR, HOFB, SAPF, SAPD, MPPA, HOFC, TOLA, SAPC, LOLB, TATB, SAPA

Annotation Cluster 12	Enrichment Score: 3.6202838797604127	
Category	Term	Genes
GOTERM_BP_FAT	GO:0034654~nucleobase, nucleoside, nucleotide and nucleic acid biosynthetic process	TMK, PURR, FEAB, NADD, PRS, NDK, ATPI, COBT, RFBB, QUEC, YBHA, YBCF, PURB, QUEA, FOLA, PYRF, PYRH, GPT, PNCB, RHO, UDK, PNCA

GOTERM_BP_FAT	GO:0044271~nitrogen compound biosynthetic process	PRS, HEMA, HEMB, PNCB, PNCA, TMK, PABC, BIRA, ATPI, PABB, ALR, QUEC, YBCF, TYRP, QUEA, PURB, AROM, PYRF, PYRH, MODC, MODE, PURR, APBE, COBU, COBT, NDK, COBS, RFBB, YBHA, LTAE, SPED, SDAC, NUDB, LEUO, NADD, IVBL, FEAB, FOLA, PHEP, FOLK, GPT, ILVY, POTB, POTA, POTD, POTC, UDK, RHO, ANSP

Annotation Cluster 13	Enrichment Score: 3.5028568868894014	
Category	Term	Genes
KEGG_PATHWAY	ecd00860:Porphyrin and chlorophyll metabolism	HEMA, HEMB, GLTX, UIDA, COBU, COBT, COBS
SP_PIR_KEYWORDS	cobalamin biosynthesis	COBU, COBT, COBS
GOTERM_BP_FAT	GO:0006779~porphyrin biosynthetic process	HEMA, HEMB, COBU, COBT, COBS
GOTERM_BP_FAT	GO:0033014~tetrapyrrole biosynthetic process	HEMA, HEMB, COBU, COBT, COBS

Annotation Cluster 14	Enrichment Score: 3.2826706153228047	
Category	Term	Genes
SP_PIR_KEYWORDS	metal-binding	PRS, PPHA, XTHA, FUMB, YCJG, MSRB, HEMB, RLMN, PHOQ, YGIQ, YDBK, NORV, TOPA, YFHL, GLTX, PDXY, TORY, HYFA, PLDA, LIGA, AMID, RSXC, RUMB, USHA, YADB, YLII, EFEB, NDK, YDHY, YBDH, RFBA, HYPF, YNFE, YBHA, YNFF, YDHX, YNFG, NUDL, SDAA, RUVC, YCHF, YJIL, NUDB, DMSB, MENC, TORC, VSR, TAUD, SDHC, YDEP, GPT, ALKB, PRIA, CCMH, YCIM
SP_PIR_KEYWORDS	metalloprotein	TORY, HYFA, YIAY, TORC, PPHA, YNFE, YNFF, YDBK, FUMB, YNFG, DMSB
SP_PIR_KEYWORDS	iron	EFEB, PPHA, YDHY, YNFE, YDHX, FUMB, YNFF, YNFG, RLMN, MHPB, YIAY, SDAA, YGIQ, YJIL, YDBK, NORV, DMSB, YFHL, TORC, TAUD, TORY, HYFA, SDHC, YDEP, RSXC, ALKB, CCMH, RUMB
GOTERM_MF_FAT	GO:0043169~cation binding	PRS, XTHA, PPHA, FUMB, EXOX, TRKG, MSRB, HEMB, RLMN, MHPB, YIAY, PHOQ, YGIQ, MDTK, YDBK, NORV, TOPA, YFHL, GLTX, PDXY, TORZ, TORY, HYFA, PLDA, LIGA, ASCB, AMID, MODC, RSXC, MODE, RUMB, USHA, YADB, YLII, EFEB, NDK, YDHY, YBDH, RFBA, HYPF, YNFE, TATD, YBHA, YNFF, YDHX, YNFG, NUDG, YDDW, NUDJ, SPED, UIDA, NUDL, YCHF, SDAA, RUVC, YJIL, NUDB, DMSB, TORA, MENC, TORC, YHJJ, VSR, TAUD, ZNUB, ZNUC, SDHC, YDEP, GPT, KEFA, ALKB, PRIA, CCMH, YCIM

Annotation Cluster 15	Enrichment Score: 3.0335992067431476	
Category	Term	Genes

KEGG_PATHWAY	ecd03420:Nucleotide excision repair	UVRC, UVRD, MFD, LIGA

Annotation Cluster 16	Enrichment Score: 2.8200660875272687	
Category	Term	Genes
GOTERM_BP_FAT	GO:0034660~ncRNA metabolic process	TTCA, YADB, ASPS, ASNS, DUSC, RLML, RLMN, TRMA, PTH, CMOA, CMOB, GIDB, YBEA, POXA, RSMF, GLTX, MNMA, RNT, TYRS, QUEC, QUEA, ARGS, YEDF, RLUE, RLUC, DUSA, RLUB, YCCW, RND, RUMB
SP_PIR_KEYWORDS	s-adenosyl-l-methionine	YBEA, RSMF, CFA, DCM, RLML, RLMN, TRMA, SPED, YCCW, YGIQ, CMOA, CMOB, GIDB, RUMB
GOTERM_BP_FAT	GO:0034470~ncRNA processing	TTCA, YBEA, RSMF, MNMA, RNT, DUSC, QUEC, QUEA, RLML, YEDF, RLMN, RLUE, TRMA, RLUC, DUSA, YCCW, RLUB, RND, CMOA, CMOB, GIDB, RUMB
SP_PIR_KEYWORDS	rrna processing	RLML, YBEA, RLMN, RLUE, RSMF, RLUC, RLUB, YCCW, GIDB, RUMB
SP_PIR_KEYWORDS	methyltransferase	YBEA, CFA, RSMF, PRMC, RRMA, YNBC, MNMA, DCM, RLML, YAFS, RLMN, TRMA, YCCW, ALKB, CMOA, CMOB, GIDB, RUMB, YBCY
GOTERM_BP_FAT	GO:0006399~tRNA metabolic process	TTCA, POXA, YADB, ASPS, GLTX, MNMA, RNT, TYRS, ASNS, DUSC, QUEC, QUEA, YEDF, ARGS, TRMA, DUSA, PTH, RND, CMOA, CMOB
GOTERM_BP_FAT	GO:0009451~RNA modification	YBEA, RSMF, MNMA, QUEC, QUEA, RLML, RLUE, TRMA, RLUC, PCNB, YCCW, RLUB, RND, CMOA, CMOB, RUMB
GOTERM_BP_FAT	GO:0006396~RNA processing	TTCA, YBEA, RSMF, MNMA, RNT, DUSC, QUEC, QUEA, RLML, YEDF, RLMN, RLUE, TRMA, RLUC, DUSA, PCNB, YCCW, RLUB, RND, CMOA, CMOB, GIDB, RUMB
GOTERM_BP_FAT	GO:0042254~ribosome biogenesis	RLML, YBEA, RLMN, RLUE, RSMF, RLUC, RLUB, YCCW, RPLT, GIDB, RUMB
GOTERM_BP_FAT	GO:0006364~rRNA processing	RLML, YBEA, RLMN, RLUE, RSMF, RLUC, RLUB, YCCW, GIDB, RUMB
GOTERM_BP_FAT	GO:0032259~methylation	RLML, YBEA, RSMF, PRMC, YCCW, DCM

Annotation Cluster 17	Enrichment Score: 2.8113707173110707	
Category	Term	Genes
SP_PIR_KEYWORDS	dna repair	UVRC, UVRD, VSR, XTHA, RECG, EXOX, REP, LIGA, MFD, RECQ, CHO, RUVC, ALKB, ALKA
GOTERM_BP_FAT	GO:0006259~DNA metabolic process	FIS, DNAA, YEES, XTHA, DCM, GYRB, INTD, HIPA, EXOX, HIPB, REP, HOLB, HOLA, YEGI, MFD, RECQ, RUVC, CHO, RECT, NUDB, DNAN, YBAV, TOPA, TOPB, INSH, UVRC, UVRD, YCIV, YNCI, RNT, VSR, RECG, HOLE, LHR, MIOC, RECE, FIME, TDK, LIGA, INTR, INTQ, PRIA, ALKB, YKFG, ALKA

Annotation Cluster 18	Enrichment Score: 2.778067085799674	
Category	Term	Genes

SP_PIR_KEYWORDS	palmitate	GFCE, GFCD, APBE, YAFT, YDDW, YEAY, MGRB, LOLB, YBFP, AMID, SPR, MLTD, YMBA, YAJI, ACRE
SP_PIR_KEYWORDS	lipoprotein	GFCE, GFCD, YDHO, YAIW, APBE, YADE, YAFT, YDDW, YEAY, MGRB, AMID, YBFP, LOLB, YBFN, SPR, MLTD, LOLE, YMBA, YDEK, YAJI, ACRE

Annotation Cluster 19	Enrichment Score: 2.775021995371978	
Category	Term	Genes
GOTERM_BP_FAT	GO:0006766~vitamin metabolic process	CAIB, MENC, MENE, NADD, FEAB, MENA, COBU, APBE, BIRA, PABC, COBT, COBS, PABB, RFBB, PDXY, FOLA, FIXB, FOLK, FIXA, NUDB, PNCB, PNCA

Annotation Cluster 20	Enrichment Score: 2.5702190815566985	
Category	Term	Genes
SP_PIR_KEYWORDS	folate biosynthesis	FOLA, FOLK, PABC, PABB, NUDB
GOTERM_BP_FAT	GO:0019438~aromatic compound biosynthetic process	AROM, FOLA, PHEP, SPED, FOLK, PABC, APBE, MODC, PABB, MODE, TYRP, NUDB

Annotation Cluster 21	Enrichment Score: 2.37776948051717	
Category	Term	Genes
SP_PIR_KEYWORDS	magnesium	MENC, PRS, NDK, VSR, XTHA, RFBA, TATD, YBHA, EXOX, YCJG, NUDG, HEMB, NUDJ, SPED, NUDL, LIGA, PHOQ, YCHF, RUVC, GPT, NUDB

Annotation Cluster 22	Enrichment Score: 2.239125122589122	
Category	Term	Genes
SP_PIR_KEYWORDS	protein biosynthesis	RPSB, RPMI, RPSA, POXA, TSF, ASPS, GLTX, TYRS, ASNS, RPMF, RPLY, INFC, PRFA, ARGS, YCIH, RPLT
GOTERM_BP_FAT	GO:0006412~translation	RPSB, RPMI, RPSA, RBBA, POXA, TSF, YADB, ASPS, GLTX, TYRS, CITG, ASNS, RPMF, RPLY, PRFA, INFC, ARGS, PTH, YCIH, RPLT
GOTERM_BP_FAT	GO:0006399~tRNA metabolic process	TTCA, POXA, YADB, ASPS, GLTX, MNMA, RNT, TYRS, ASNS, DUSC, QUEC, QUEA, YEDF, ARGS, TRMA, DUSA, PTH, RND, CMOA, CMOB

Annotation Cluster 23	Enrichment Score: 2.2004469576790546	
Category	Term	Genes
SP_PIR_KEYWORDS	electron transport	YDIR, TORC, MIOC, YNFG, TORY, HYFA, SDHC, FIXB, FIXA, RSXC, YDBK, NORV, DMSB
GOTERM_BP_FAT	GO:0022900~electron transport chain	YDIR, TORC, MIOC, YNFG, YDHU, YNFH, TORY, HYFA, SDHC, FIXB, FIXA, RSXC, YDBK, NORV, DMSC, DMSB

GOTERM_BP_FAT	GO:0006091~generation of precursor metabolites and energy	NARP, FRDD, HYPF, FUMB, YNFG, YDHU, YNFH, YDBK, NORV, DMSC, DMSB, TORA, YDIR, MENC, MENE, TORC, MENA, ATPI, MIOC, PDXY, TORY, UBIC, HYFA, FIXB, SDHC, FIXA, MLTD, RSXC, RHO
GOTERM_MF_FAT	GO:0009055~electron carrier activity	YDHO, YDHY, YNFE, YDHX, YNFF, YNFG, YDHU, YDHV, YDBK, YHIN, NORV, DMSB, YDIR, TORA, YDIS, YFHL, TORC, TAUD, TORZ, TORY, HYFA, FIXB, SDHC, FIXA, YCAK, RSXC, YDGJ

Annotation Cluster 24	Enrichment Score: 2.0931419364395585	
Category	Term	Genes
GOTERM_MF_FAT	GO:0042280~cell surface antigen activity, host-interacting	RFAG, RFBA, RFC, CLD, RFBB, WAAU, LPXM, RFAY, UGD, ASMA, GLF, WAAA, RFBX, LPXH

Annotation Cluster 25	Enrichment Score: 2.053075187608088	
Category	Term	Genes
GOTERM_BP_FAT	GO:0009061~anaerobic respiration	TORA, MENC, NARP, MENE, FRDD, TORC, MENA, HYPF, FUMB, YNFH, HYFA, MLTD, DMSC, DMSB
GOTERM_BP_FAT	GO:0006091~generation of precursor metabolites and energy	NARP, FRDD, HYPF, FUMB, YNFG, YDHU, YNFH, YDBK, NORV, DMSC, DMSB, TORA, YDIR, MENC, MENE, TORC, MENA, ATPI, MIOC, PDXY, TORY, UBIC, HYFA, FIXB, SDHC, FIXA, MLTD, RSXC, RHO
GOTERM_BP_FAT	GO:0045333~cellular respiration	TORA, MENC, NARP, FRDD, MENE, TORC, MENA, HYPF, FUMB, YDHU, YNFH, UBIC, HYFA, SDHC, MLTD, DMSC, DMSB
GOTERM_BP_FAT	GO:0015980~energy derivation by oxidation of organic compounds	TORA, MENC, NARP, FRDD, MENE, TORC, MENA, HYPF, FUMB, YDHU, YNFH, UBIC, HYFA, SDHC, MLTD, DMSC, DMSB

Annotation Cluster 26	Enrichment Score: 2.0181174053842446	
Category	Term	Genes
GOTERM_BP_FAT	GO:0032392~DNA geometric change	REP, TOPA, TOPB, UVRD
GOTERM_BP_FAT	GO:0006268~DNA unwinding during replication	REP, TOPA, TOPB, UVRD
GOTERM_BP_FAT	GO:0032508~DNA duplex unwinding	REP, TOPA, TOPB, UVRD

Annotation Cluster 27	Enrichment Score: 1.986185618719747	
Category	Term	Genes
SP_PIR_KEYWORDS	carbon-oxygen lyase	CAIB, HEMB, SDAA, CITG, RFBB, FUMB

Annotation Cluster 28	Enrichment Score: 1.7973700508440689	
Category	Term	Genes
GOTERM_MF_FAT	GO:0042623~ATPase activity, coupled	RBBA, TBPA, YCBZ, MSBA, UVRD, MDLA, MDLB, COBT, TAUB, COBS, RECG, LHR, YBHA, ZNUB, REP, SBMA, YOAA, MFD, RECQ, MODC, MODE, PRIA, POTA
GOTERM_MF_FAT	GO:0016887~ATPase activity	RBBA, MSBA, COBT, COBS, RFBB, YBHA, YNJD, REP, SBMA, YOAA, SSUB, MFD, RECQ, TBPA, YCBZ, UVRD, MDLA, MDLB, TAUB, RECG, LHR, UUP, ZNUB, SAPF, ZNUC, SAPD, MODC, YEHX, MODE, PRIA, POTA

Annotation Cluster 29	Enrichment Score: 1.7758767688620878	
Category	Term	Genes
SP_PIR_KEYWORDS	protein biosynthesis	RPSB, RPMI, RPSA, POXA, TSF, ASPS, GLTX, TYRS, ASNS, RPMF, RPLY, INFC, PRFA, ARGS, YCIH, RPLT

Annotation Cluster 30	Enrichment Score: 1.7359390499433547	
Category	Term	Genes
KEGG_PATHWAY	eum00760:Nicotinate and nicotinamide metabolism	USHA, NADD, PNCB, PNCA
GOTERM_BP_FAT	GO:0006733~oxidoreduction coenzyme metabolic process	UBIC, NADD, FEAB, SPED, RPIR, RPIB, RFBB, PNCB, PNCA
GOTERM_BP_FAT	GO:0043603~cellular amide metabolic process	NADD, FEAB, BIRA, RPIR, RPIB, RFBB, GLXK, PNCB, PNCA, ALLD

Annotation Cluster 31	Enrichment Score: 1.7051146459393889	
Category	Term	Genes
UP_SEQ_FEATURE	domain:GGDEF	YEAJ, YDEH, YNEF, YFIN, YEGE, YLIF, YEDQ, YEAP
INTERPRO	IPR000160:Diguanylate cyclase, predicted	YEAJ, YDEH, YNEF, YFIN, YEGE, YLIF, YEDQ, YEAP
SMART	SM00267:DUF1	YEAJ, YDEH, YNEF, YFIN, YEGE, YLIF, YEDQ, YEAP

Annotation Cluster 32	Enrichment Score: 1.702535503602171	
Category	Term	Genes
KEGG_PATHWAY	eco00130:Ubiquinone and other terpenoid-quinone biosynthesis	MENC, UBIC, MENE, MENA
GOTERM_BP_FAT	GO:0042373~vitamin K metabolic process	MENC, MENE, MENA
Annotation Cluster 33	Enrichment Score: 1.658411641193403	
Category	Term	Genes
GOTERM_BP_FAT	GO:0006596~polyamine biosynthetic process	PURR, SPED, POTB, POTA, POTD, POTC
GOTERM_BP_FAT	GO:0006576~biogenic amine metabolic process	CAIB, PURR, RSSA, SPED, FIXB, FIXA, POTB, POTA, POTD, POTC
GOTERM_BP_FAT	GO:0006575~cellular amino acid derivative metabolic process	CAIB, PURR, RSSA, SPED, FIXB, FIXA, POTB, POTA, POTD, POTC
Annotation Cluster 34	Enrichment Score: 1.6479465883471112	
Category	Term	Genes
SP_PIR_KEYWORDS	cell wall biogenesis/degradation	MRCB, YCJG, MRDA, MLTF, MRCA, AMID, AMIC, ALR, MLTD
GOTERM_BP_FAT	GO:0044036~cell wall macromolecule metabolic process	MLTF, ARRD, YIEL, ALR, YAGH, HIPA, HIPB, MRCB, MRDA, PRC, AMPC, MRCA, AMIC, ESSD, MLTD
SP_PIR_KEYWORDS	multifunctional enzyme	MRCB, USHA, MRDA, MRCA, COBU, YAGH
GOTERM_BP_FAT	GO:0000270~peptidoglycan metabolic process	MRCB, MRDA, ARRD, MPPA, PRC, AMPC, MRCA, AMID, AMIC, ALR, HIPA, HIPB
GOTERM_BP_FAT	GO:0045229~external encapsulating structure organization	MRCB, ETK, MRDA, MLTF, ECPD, MRCA, AMID, AMIC, YCBF, ALR, MLTD, YCBR

GOTERM_BP_FAT	GO:0007047~cell wall organization	MRCB, MRDA, MLTF, ECPD, MRCA, AMID, AMIC, YCBF, ALR, MLTD, YCBR
GOTERM_BP_FAT	GO:0022604~regulation of cell morphogenesis	MRCB, MRDA, MRDB, MRCA, ALR
GOTERM_BP_FAT	GO:0008360~regulation of cell shape	MRCB, MRDA, MRDB, MRCA, ALR
GOTERM_MF_FAT	GO:0031406~carboxylic acid binding	MRCB, MRDA, MRCA, TAUD

Annotation Cluster 35	Enrichment Score: 1.5605840048726978	
Category	Term	Genes
GOTERM_BP_FAT	GO:0008033~tRNA processing	TTCA, YEDF, TRMA, DUSA, MNMA, RNT, RND, DUSC, QUEC, CMOA, CMOB, QUEA
GOTERM_BP_FAT	GO:0006400~tRNA modification	TRMA, MNMA, QUEC, CMOA, CMOB, QUEA

Annotation Cluster 36	Enrichment Score: 1.5539025681134444	
Category	Term	Genes
GOTERM_BP_FAT	GO:0044271~nitrogen compound biosynthetic process	PRS, HEMA, HEMB, PNCB, PNCA, TMK, PABC, BIRA, ATPI, PABB, ALR, QUEC, YBCF, TYRP, QUEA, PURB, AROM, PYRF, PYRH, MODC, MODE, PURR, APBE, COBU, COBT, NDK, COBS, RFBB, YBHA, LTAE, SPED, SDAC, NUDB, LEUO, NADD, IVBL, FEAB, FOLA, PHEP, FOLK, GPT, ILVY, POTB, POTA, POTD, POTC, UDK, RHO, ANSP
GOTERM_BP_FAT	GO:0009309~amine biosynthetic process	LEUO, PURR, IVBL, PABC, ALR, PABB, QUEC, YBCF, TYRP, LTAE, AROM, FOLA, PYRH, PHEP, SPED, FOLK, SDAC, ILVY, POTB, POTA, NUDB, POTD, ANSP, POTC
GOTERM_BP_FAT	GO:0046394~carboxylic acid biosynthetic process	LEUO, PURR, IVBL, PLSX, PABC, BIRA, FABF, ALR, PABB, QUEC, YBCF, TYRP, LTAE, AROM, FOLA, UBIC, PYRH, PHEP, ACPH, FOLK, SDAC, ILVY, NUDB, ANSP

Annotation Cluster 37	Enrichment Score: 1.5406026077580997	
Category	Term	Genes
PIR_SUPERFAMILY	PIRSF004763:transporter, EamA type	YICL, EAMA, YEDA, RHTA
UP_SEQ_FEATURE	domain:DUF6 1	YICL, EAMA, YEDA, RHTA
UP_SEQ_FEATURE	domain:DUF6 2	YICL, EAMA, YEDA, RHTA

INTERPRO	IPR000620:Protein of unknown function DUF6, transmembrane	YDDG, YICL, EAMA, YEDA, RHTA

Annotation Cluster 38	Enrichment Score: 1.5068746619855726	
Category	Term	Genes
GOTERM_BP_FAT	GO:0009432~SOS response	RFAG, UVRC, YDJM, UVRD, RECQ, CHO
GOTERM_BP_FAT	GO:0009991~response to extracellular stimulus	RFAG, YCAI, UVRC, YDJM, UVRD, RECQ, RSPA, CHO

Annotation Cluster 39	Enrichment Score: 1.5007453875427932	
Category	Term	Genes
SP_PIR_KEYWORDS	iron-sulfur protein	HYFA, RSXC, YNFE, YNFF, YDBK, FUMB, YNFG, DMSB
SP_PIR_KEYWORDS	4fe-4s	YFHL, YDHY, YNFE, YNFF, FUMB, YDHX, YNFG, RLMN, HYFA, YDEP, SDAA, YGIQ, RSXC, YJIL, YDBK, DMSB, RUMB
SP_PIR_KEYWORDS	iron-sulfur	YFHL, YDHY, YNFE, YNFF, FUMB, YDHX, YNFG, RLMN, HYFA, YDEP, SDAA, YGIQ, RSXC, YJIL, YDBK, DMSB, RUMB
PIR_SUPERFAMILY	PIRSF000246:NrfC protein	HYFA, YNFG, DMSB
GOTERM_MF_FAT	GO:0051540~metal cluster binding	YFHL, YDHY, YNFE, YNFF, FUMB, YDHX, YNFG, YDHV, RLMN, HYFA, YDEP, YGIQ, SDAA, RSXC, YJIL, YDBK, DMSB, RUMB
GOTERM_MF_FAT	GO:0051536~iron-sulfur cluster binding	YFHL, YDHY, YNFE, YNFF, FUMB, YDHX, YNFG, YDHV, RLMN, HYFA, YDEP, YGIQ, SDAA, RSXC, YJIL, YDBK, DMSB, RUMB

Annotation Cluster 40	Enrichment Score: 1.4402017226006012	
Category	Term	Genes
KEGG_PATHWAY	ecd00020:Citrate cycle (TCA cycle)	FRDD, CITD, SDHC, FUMB

Annotation Cluster 41	Enrichment Score: 1.4393806207571282	
Category	Term	Genes
GOTERM_BP_FAT	GO:0019395~fatty acid oxidation	PRPR, MHPA, MHPB, PRPE, PRPB

GOTERM_BP_FAT	GO:0006631~fatty acid metabolic process	PRPR, MHPA, MHPB, ACPH, PLSX, FABF, PRPE, FADK, PRPB
Annotation Cluster 42	Enrichment Score: 1.4227698126905468	
Category	Term	Genes
KEGG_PATHWAY	ect00564:Glycerophospholipid metabolism	PLDA, YNBB, PGPB, PGSA
Annotation Cluster 43	Enrichment Score: 1.4146110658050493	
Category	Term	Genes
KEGG_PATHWAY	ecc03410:Base excision repair	LIGA, XTHA, ALKA
Annotation Cluster 44	Enrichment Score: 1.4115261332668156	
Category	Term	Genes
GOTERM_BP_FAT	GO:0034621~cellular macromolecular complex subunit organization	PRFA, TORC, SDHC, MLTD, CCMG, CCMH, RPLT, RHO, CCMF
GOTERM_BP_FAT	GO:0034622~cellular macromolecular complex assembly	TORC, SDHC, MLTD, CCMG, CCMH, RPLT, CCMF
GOTERM_BP_FAT	GO:0017004~cytochrome complex assembly	TORC, SDHC, MLTD, CCMG, CCMH, CCMF
SP_PIR_KEYWORDS	heme	TORY, TORC, SDHC, EFEB, CCMH
GOTERM_BP_FAT	GO:0043933~macromolecular complex subunit organization	PRFA, TORC, SDHC, MLTD, CCMG, CCMH, RPLT, RHO, CCMF
Annotation Cluster 45	Enrichment Score: 1.392104367613481	
Category	Term	Genes
SP_PIR_KEYWORDS	phosphoprotein	YEDV, ETK, RPSA, YEDW, NARP, CITD, KDPD, PPHA, NDK, ALR, RSSB, CAIC, LTAE, INFC, UHPB, CMTA, UHPA, BAES, UGD, PHOQ, PHOP
SP_PIR_KEYWORDS	two-component regulatory system	YEDV, YEDW, PRPR, NARP, UHPB, UHPA, BAES, PHOQ, KDPD, PHOP, RSSB

GOTERM_BP_FAT	GO:0000160~two-component signal transduction system (phosphorelay)	YEDV, NARP, YEDW, KDPD, RSSB, SDIA, PRPR, YEGE, UHPB, MATA, UHPA, BAES, PHOQ, PHOP
Annotation Cluster 46	Enrichment Score: 1.382769664742016	
Category	Term	Genes
SP_PIR_KEYWORDS	nuclease	RNB, RNT, RND, VSR, XTHA, RUVC, TATD, RECE, EXOX
GOTERM_BP_FAT	GO:0006308~DNA catabolic process	UVRC, RNT, VSR, XTHA, RECE, EXOX
GOTERM_BP_FAT	GO:0044265~cellular macromolecule catabolic process	UVRC, RNB, RNT, RND, VSR, XTHA, RECE, EXOX
GOTERM_BP_FAT	GO:0009057~macro molecule catabolic process	UVRC, YIEL, ARRD, RNT, VSR, XTHA, YAGH, RECE, EXOX, RNB, AMID, RND, AMIC
SP_PIR_KEYWORDS	exonuclease	RNB, RNT, RND, XTHA, RECE, EXOX
GOTERM_MF_FAT	GO:0004527~exonucl ease activity	HOLB, RNB, RNT, RND, XTHA, RECE, EXOX, DNAN
Annotation Cluster 47	Enrichment Score: 1.369053927775673	
Category	Term	Genes
UP_SEQ_FEATURE	domain:HTH lysR-type	LEUO, YDHB, YFER, YNFL, CYNR, PERR, YEAT, ILVY, YBHD, XAPR, ABGR, YHJC
INTERPRO	IPR000847:Bacterial regulatory protein, LysR	LEUO, YNFL, YFER, CYNR, YEAT, YBHD, ABGR, YHJC, YDHB, PERR, ILVY, MODE, XAPR
INTERPRO	IPR011991:Winged helix repressor DNA-binding	LEUO, NARP, YFER, YNFL, DGOR, YBAO, BIRA, CYNR, YEAT, YBHD, ABGR, YHJC, REP, SDIA, YDHB, MATA, UHPA, PERR, ILVY, MODE, XAPR, YDFH
Annotation Cluster 48	Enrichment Score: 1.3564123034236524	
Category	Term	Genes
SP_PIR_KEYWORDS	helicase	REP, YOAA, UVRD, MFD, RECQ, YEJH, RECG, LHR, PRIA, RHO
GOTERM_MF_FAT	GO:0008026~ATP-dependent helicase activity	REP, YOAA, UVRD, MFD, RECQ, RECG, LHR, PRIA
GOTERM_MF_FAT	GO:0004386~helicas e activity	REP, YOAA, UVRD, MFD, RECQ, YEJH, RECG, LHR, PRIA, RHO

Annotation Cluster 49	Enrichment Score: 1.3381057234748042	
Category	Term	Genes
KEGG_PATHWAY	eci03018:RNA degradation	PCNB, RECQ, RHO
