# Supplementary material for: Central role for RNase YbeY in Hfq-dependent and Hfq-independent small-RNA regulation in bacteria
Source: BMC Genomics. 2014 Feb 11;15:121. doi: 10.1186/1471-2164-15-121 (PMC3933206; doi:10.1186/1471-2164-15-121)
Supplement: Additional file 5: Table S5 — Functional clusters of genes differentially down-regulated in ΔybeY vs. WT under unstressed state. [file 1471-2164-15-121-S5.pdf]

Supplemental Table 5. Functional clusters of genes differentially down-regulated in *ΔybeY* vs. WT under unstressed state

Annotation Cluster 1	Enrichment Score: 30.118016535325918	
Category	Term	Genes
GOTERM_BP_FAT	GO:0009061~anaerobic respiration	TDCE, NAPG, NAPH, FDNG, NAPF, HYPC, FDNI, NAPC, FDNH, HYPD, NARX, NAPD, NAPA, NAPB, NDH, HYPA, NRFD, NRFE, NRFB, NRFC, ACNB, NRFF, NRFG, NARH, NARG, YFID, NIKD, NARJ, LLDD, NARI, NIKE, NARL, GLTA, HYBG, HYBF, NRFA, HYBC, GLPB, HYBD, GLPA, HYBB, HYBO, GLPC, NIRB, YBIY, NIRD
GOTERM_BP_FAT	GO:0015980~energy derivation by oxidation of organic compounds	TDCE, LDHA, NARX, NRFD, NRFE, NRFB, NRFC, ACNB, NRFF, NRFG, NARH, NARG, YFID, NARJ, NIKD, NARI, NIKE, NARL, GLTA, GLGB, HYBG, GLGC, HYBF, GLPB, HYBC, GLPA, HYBD, HYBB, HYBO, UBIX, GLPD, GLPC, GLGP, SUCB, ARCB, GLGX, UBIH, FRMA, NAPG, NAPH, FDNG, NAPF, HYPC, FDNI, NAPC, HYPD, FDNH, NAPD, NAPA, NAPB, NDH, HYPA, HYPB, GLGA, LLDD, NRFA, NIRB, YBIY, NIRD
GOTERM_BP_FAT	GO:0006091~generation of precursor metabolites and energy	TDCE, LDHA, NARX, NRFD, NRFE, NRFB, NRFC, ACNB, NRFF, ZNTA, NRFG, NARH, PGI, NARG, YFID, NIKD, NARJ, NIKE, NARI, NARL, GLTA, PGK, GLGB, HYBG, GLGC, GLCF, HYBF, GLPB, HYBC, GLPA, HYBD, HYBB, UBIX, HYBO, GLPD, GLPC, GLGP, SUCB, ARCB, GPMA, DIPZ, GLGX, UBIH, YGCO, FRMA, NAPG, NAPH, FDNG, NAPF, NAPC, FDNI, HYPC, HYPD, FDNH, NAPD, FBAA, NAPA, NAPB, NDH, HYPA, HYPB, YQCA, GLGA, LLDD, NRFA, NIRB, CYBC, YBIY, NIRD, CSRA, GLK, YFAE
GOTERM_BP_FAT	GO:0045333~cellular respiration	TDCE, NARX, NRFD, NRFE, NRFB, NRFC, ACNB, NRFF, NRFG, NARH, NARG, YFID, NARJ, NIKD, NARI, NIKE, NARL, GLTA, HYBG, HYBF, HYBC, GLPB, GLPA, HYBD, HYBB, HYBO, UBIX, GLPD, GLPC, SUCB, ARCB, UBIH, NAPG, NAPH, FDNG, NAPF, HYPC, FDNI, NAPC, FDNH, NAPD, HYPD, NAPA, NAPB, HYPA, NDH, LLDD, NRFA, NIRB, YBIY, NIRD

Annotation Cluster 2	Enrichment Score: 17.021450432870655	
Category	Term	Genes
SP_PIR_KEYWORDS	metal-binding	MAEB, CPDB, TDCG, GLDA, APHA, GLPK, ALAS, NRFB, NRFC, LUXS, ACNB, GUDD, KATG, NRFF, ZNTA, NARH, NARG, GLTD, RECR, NARI, NANK, MALS, YQHD, PGM, HEMN, GLTB, GLCF, ARGE, HYBF, YBGI, PEPD, HYBC, HYBD, MOEA, HYBB, GLPF, HYBO, GLPC, CLPX, NIKR, GSHB, YGCO, YBJI, PEPP, FRMA, PTRA, GUDX, NAPG, NAPH, FDNG, NAPF, NAPC, FDNI, HYPD, FBAA, FDNH, ISPH, NAPA, NAPB, AMPD, HYPA, HYPB, NUDF, MOAA, PTSA, CDD, YFGC, NRDB, TAG, YGGW, YFBR, NRFA, FUCI, IDND, DAPE, YAEI, NIRB, CYBC, YBIY, HFLB, ISPB, YBEY, GLMM, DEOB, YFAW, SODA, FUCA, YFAE
SP_PIR_KEYWORDS	iron	NAPG, NAPH, FDNG, TDCG, NAPF, FDNI, NAPC, FDNH, ISPH, NAPA, NAPB, NRFB, NRFC, ACNB, LUXS, MOAA, KATG, NRFF, NARH, GLTD, FECA, NARG, FECB, FECC, NARI, NRDB, HEMN, GLTB, YGGW, GLCF, NRFA, HYBC, FECE, FECD, HYBB, HYBO, FUCO, FECR, GLPC, NIRB, CYBC, YBIY, YGCO, YFAE

GOTERM_MF_FAT	GO:0043167~ion binding	MAEB, GLDA, QOR, APHA, ALAS, GUDD, KATG, FECA, GLTD, NIKD, FECB, RECR, NIKE, FECC, NANK, MALS, YQHD, PGM, NIKC, MALQ, GLTB, GLCF, ARGE, HYBF, HYBC, HYBD, FECE, HYBB, FECD, HYBO, FECR, CLPX, NIKR, YGCO, YBJI, LNT, PTRA, MIAA, PPX, NAPG, NAPH, NAPF, NAPC, FBAA, NAPA, ISPH, NAPB, YQCA, NUDF, MOAA, PTSA, CDD, YFGC, NRDB, YGGW, NRFA, FTNB, DAPE, NIRB, YBEY, GLMM, DEOB, YFAE, CPDB, TDCG, PCK, GLPK, YJCE, NRFB, NRFC, LUXS, ACNB, ZNTA, NRFF, NARH, NARG, NARI, HEMN, GLGB, YIIM, PEPD, YBGI, MOEA, GLPF, GLPC, ZUPT, DGT, GLGX, GSHB, PEPP, FRMA, GUDX, FDNG, YIDK, FDNI, HYPD, FDNH, AMPD, HYPA, HYPB, YHDH, YFBR, FUCI, IDND, FUCO, NADK, YAEI, CYBC, YBIY, HFLB, BGLA, SODA, YFAW, CLCA, FUCA, KEFB

Annotation Cluster 3	Enrichment Score: 12.976456235337231	
Category	Term	Genes
SP_PIR_KEYWORDS	membrane	GUDP, NANC, YGAZ, YQAA, MALF, YOHJ, MALG, ATOS, NANT, MALK, YGBE, FECA, NIKD, NIKE, FECC, NIKC, ACRD, HYBC, FECE, HYBB, FECD, SYD, HYBO, YJET, YCCA, GLTP, NUPG, YHHQ, ARGO, DIPZ, YBJP, YGDR, LNT, YHHN, YTFB, PPX, NAPH, NAPC, OMPC, PPK, YFGA, GLNQ, YFGG, LAMB, GLNP, YGGT, YQJE, YHAH, MTGA, YJHB, PHNC, CPXA, PHNE, CCMA, NIRC, YHFL, TSX, YIHN, TDCC, DCUA, DCUC, NARX, YFEO, NRFD, YJCE, NRFE, ZNTA, NARH, NARG, NARI, YAFY, NARK, GLPB, GSPO, GLPA, GSPL, GSPM, GLPF, GLPC, YPDA, RSEA, ZUPT, XYLE, RSEC, XYLH, MURG, YHCB, ARCB, EVGS, AAEX, YIDK, FDNI, FDNH, YJJP, ENVZ, YJJL, NDH, FTSW, YJJB, YPJD, YJIY, YFJD, FUCP, HFLC, HFLB, HFLK, YGJR, YFAV, CLCA, DEDA, KEFB

Annotation Cluster 4	Enrichment Score: 9.590868001005862	
Category	Term	Genes
SP_PIR_KEYWORDS	phosphoprotein	THRC, FDNG, NARX, ENVZ, ATOS, PPK, ZNTA, PTSA, CPXR, NARL, TDCB, PGM, GLNL, MALP, RFFA, GLNG, EVGA, GLNA, CPXA, GLGP, YPDB, NPR, GCVP, ARCB, GPMA, EVGS, BAER, QSEB, GLMM, OMPR, BASR
KEGG_PATHWAY	ect02020:Two-component system	FDNG, FDNI, NARX, FDNH, ENVZ, OMPC, ATOS, NARH, NARG, NARJ, CPXR, NARI, NARL, GLNL, GLNG, EVGA, GLNA, CPXA, ARCB, EVGS, BAER, QSEB, OMPR, BASR, CSRA

Annotation Cluster 5	Enrichment Score: 9.449743575603822	
Category	Term	Genes
SP_PIR_KEYWORDS	periplasm	CPDB, FDNG, YTFJ, YCFS, NAPA, NAPB, MALE, APHA, TESA, MALM, NRFB, NRFF, NANM, FECB, MALS, SLT, GLNH, NRFA, HYBO, DSBA, FECR, DACB, RSEB, CYBC, RNA, FKLB, PPIA, HISJ, PTRA, DCRB

SP_PIR_KEYWORDS	signal	NANC, CPDB, MALG, YCFS, MALE, APHA, YPEC, MALM, TESA, NRFB, NRFC, NRFF, FECA, NANM, FECB, FECC, YAFY, MALS, SLT, NIKC, YACC, HYBO, RSEB, XYLE, RNA, EVGS, DIPZ, YBJP, YGDR, LNT, DCRB, PTRA, NAPG, FDNG, YHDP, YTFJ, NAPA, YNFD, ENVZ, NAPB, OMPC, YJEI, LAMB, YPFG, YFGC, GLNH, NRFA, DSBA, DACB, YJHS, CYBC, HFLB, PPIA, YEEZ, TSX, HISJ

Annotation Cluster 6	Enrichment Score: 8.920744709462836	
Category	Term	Genes
SP_PIR_KEYWORDS	metalloprotein	NRFA, HYBC, FDNG, NAPC, HYBO, FUCO, FDNH, NRFB, NRFC, YBIY, KATG, NARH, NARG, NARI, SODA, GLTB, FRMA
SP_PIR_KEYWORDS	electron transfer	NRFB, NRFC, NRFA, NAPC, NARH, FDNH, NARG, NARI
SP_PIR_KEYWORDS	iron-sulfur protein	NRFC, FDNG, NARH, FDNH, NARG, GLTB

Annotation Cluster 7	Enrichment Score: 8.12897092247381	
Category	Term	Genes
GOTERM_BP_FAT	GO:0043623~cellular protein complex assembly	NRFA, HYBB, NAPC, FDNI, NAPB, NRFE, NRFB, CCMA, CYBC, NRFF, DIPZ, FTSZ, NRFG, YPJD, NARI
GOTERM_BP_FAT	GO:0017004~cytochrome complex assembly	NRFA, HYBB, FDNI, NAPC, NAPB, NRFE, NRFB, CCMA, CYBC, NRFF, DIPZ, NRFG, NARI
GOTERM_BP_FAT	GO:0006461~protein complex assembly	NRFA, HYBB, NAPC, FDNI, RPON, NAPB, HYPB, NRFE, NRFB, CCMA, CYBC, NRFF, NRFG, DIPZ, FTSZ, YPJD, NARI

Annotation Cluster 8	Enrichment Score: 7.7692984628488695	
Category	Term	Genes
SP_PIR_KEYWORDS	nitrate assimilation	NIRB, NIRD, NIRC, NARH, NARG, NARX, NAPA, NARJ, NARI, NARL, NARK
GOTERM_MF_FAT	GO:0016661~oxidoreductase activity, acting on other nitrogenous compounds as donors	NRFA, NAPG, NAPH, NAPC, NAPD, NAPA, NAPB, QUEF, NIRB, NRFB, NRFC, NIRD, NARH, NARG, NARJ, NARI
GOTERM_BP_FAT	GO:0042128~nitrate assimilation	NIRB, NIRD, NIRC, NARH, NARG, NARX, NAPA, NARJ, NARI, NARL, NARK
KEGG_PATHWAY	ecr00910:Nitrogen metabolism	NIRB, NRFA, GLNA, NIRD, NARH, GLTD, NARG, GCVT, NAPA, NARJ, NARI, GLTB

Annotation Cluster 9	Enrichment Score: 5.687259051500477	
Category	Term	Genes
KEGG_PATHWAY	ecx00500:Starch and sucrose metabolism	GLGB, GLGC, GLGA, MAK, PGI, MALS, PGM, GLK, GLGP, MALQ, MALP

Annotation Cluster 10	Enrichment Score: 4.795743011322636	
Category	Term	Genes

KEGG_PATHWAY	ecd00520:Amino sugar and nucleotide sugar metabolism	NANE, GLGC, NAGB, MAK, NAGA, PGI, GLMM, NANK, NANA, PGM, GLK
GOTERM_BP_FAT	GO:0046364~monosaccharide biosynthetic process	NANE, TDCG, PCK, NANA, NANT, NAGB, MURG, GPMA, NAGA, PGI, NANK, CSRA, PGK

Annotation Cluster 11	Enrichment Score: 4.57442873804627	
Category	Term	Genes
SP_PIR_KEYWORDS	glycolysis	GPMA, PGI, FBAA, GLK, PGK
KEGG_PATHWAY	ecd00010:Glycolysis / Gluconeogenesis	GALM, GPMA, PCK, PGI, FBAA, BGLA, PGM, GLK, PGK, FRMA

Annotation Cluster 12	Enrichment Score: 4.302372669253555	
Category	Term	Genes
GOTERM_BP_FAT	GO:0046364~monosaccharide biosynthetic process	NANE, TDCG, PCK, NANA, NANT, NAGB, MURG, GPMA, NAGA, PGI, NANK, CSRA, PGK
GOTERM_BP_FAT	GO:0046165~alcohol biosynthetic process	NANE, TDCG, PCK, NANA, NANT, NAGB, MURG, GPMA, NAGA, PGI, NANK, CSRA, PGK
SP_PIR_KEYWORDS	gluconeogenesis	GPMA, TDCG, PCK, PGI, FBAA, PGK
GOTERM_BP_FAT	GO:0016051~carbohydrate biosynthetic process	NANE, TDCG, PCK, RFFM, NANA, FTSW, GLGA, NANT, NAGB, NAGA, PGI, NANK, SLT, RFFA, PGK, GLGB, GLGC, MTGA, DACB, GLGP, MURD, LPCA, MURG, GPMA, GMHB, GLGX, GLMM, CSRA
GOTERM_BP_FAT	GO:0006090~pyruvate metabolic process	GPMA, TDCG, PCK, PGI, ISPH, CSRA, PGK

Annotation Cluster 13	Enrichment Score: 4.235191863088521	
Category	Term	Genes
GOTERM_BP_FAT	GO:0019318~hexose metabolic process	TDCE, TDCG, PCK, FBAA, GLGA, GALM, YGBJ, PGI, RPIA, FUCU, PGM, PGL, PGK, GLGB, GLGC, FSAB, FUCK, FUCI, FUCO, GALR, GLGP, FUCP, YBIY, GPMA, GLGX, GLMM, CSRA, GLK, FUCA
SP_PIR_KEYWORDS	Isomerase	NANE, KDUI, FUCI, DSBA, GALM, LPCA, FKLB, NAGB, PPIA, GPMA, PGI, NANM, GLMM, DEOB, RPIA, FUCU, PGM, TRUA, FRLB
SP_PIR_KEYWORDS	glycolysis	GPMA, PGI, FBAA, GLK, PGK
INTERPRO	IPR005952:Phosphoglycerate mutase 1	GPMA
GOTERM_BP_FAT	GO:0006006~glucose metabolic process	GLGB, TDCE, GLGC, FSAB, TDCG, PCK, FBAA, GLGP, GLGA, YGBJ, YBIY, GPMA, GLGX, PGI, GLMM, RPIA, PGM, CSRA, GLK, PGL, PGK

Annotation Cluster 14	Enrichment Score: 4.022734934851986	
Category	Term	Genes
SP_PIR_KEYWORDS	electron transfer	NRFB, NRFC, NRFA, NAPC, NARH, FDNH, NARG, NARI

Annotation Cluster 15	Enrichment Score: 3.7910514387542076	
Category	Term	Genes
SP_PIR_KEYWORDS	iron	NAPG, NAPH, FDNG, TDCG, NAPF, FDNI, NAPC, FDNH, ISPH, NAPA, NAPB, NRFB, NRFC, ACNB, LUXS, MOAA, KATG, NRFF, NARH, GLTD, FECA, NARG, FECB, FECC, NARI, NRDB, HEMN, GLTB, YGGW, GLCF, NRFA, HYBC, FECE, FECD, HYBB, HYBO, FUCO, FECR, GLPC, NIRB, CYBC, YBIY, YGCO, YFAE
SP_PIR_KEYWORDS	4fe-4s	GLCF, NAPG, NAPH, FDNG, TDCG, NAPF, HYBO, FDNH, NAPA, GLPC, NIRB, NRFC, ACNB, YBIY, MOAA, NARH, GLTD, NARG, HEMN, YGGW
GOTERM_MF_FAT	GO:0051536~iron-sulfur cluster binding	NAPG, NAPH, TDCG, FDNG, NAPF, FDNH, NAPA, ISPH, NRFC, ACNB, MOAA, NARH, NARG, GLTD, HEMN, YGGW, GLTB, GLCF, HYBO, GLPC, NIRB, YBIY, NIRD, YGCO, YFAE
GOTERM_MF_FAT	GO:0051540~metal cluster binding	NAPG, NAPH, TDCG, FDNG, NAPF, FDNH, NAPA, ISPH, NRFC, ACNB, MOAA, NARH, NARG, GLTD, HEMN, YGGW, GLTB, GLCF, HYBO, GLPC, NIRB, YBIY, NIRD, YGCO, YFAE
GOTERM_MF_FAT	GO:0009055~electron carrier activity	NAPG, NAPH, FDNG, NAPF, FDNH, NAPA, NDH, YQCA, NRFC, GOR, GLTD, NARG, LLDD, YQJH, YJHC, GLCF, GLPB, AHPF, MDAB, GLPC, NIRB, CYBC, YHHX, NIRD, DIPZ, YGCO, YGJR, YGCN, YFAE
Annotation Cluster 16	Enrichment Score: 3.5104327916440012	
Category	Term	Genes
KEGG_PATHWAY	ecw00030:Pentose phosphate pathway	PGI, FBAA, DEOC, DEOB, RPIA, PGM, PGL
Annotation Cluster 17	Enrichment Score: 3.1811470414951293	
Category	Term	Genes
SP_PIR_KEYWORDS	Molybdenum cofactor biosynthesis	MOAE, MOAD, MOAC, MOAB, MOAA, MOEA
GOTERM_BP_FAT	GO:0006732~coenzyme metabolic process	FSAB, MOEA, NAPA, SUCB, MOAE, FOLP, YGBJ, MOAD, MOAC, ACNB, GOR, MOAB, MOAA, GSHA, UBIH, GSHB, RPIA, YFAY, GLTA, UBIF
GOTERM_BP_FAT	GO:0051186~cofactor metabolic process	NAPA, MOAE, MOAD, YGBJ, MOAC, GOR, MOAB, ACNB, MOAA, RPIA, HEMN, GLTA, YGGW, FSAB, EUTT, CYSG, MOEA, SUCB, BIOH, FOLP, UBIH, GSHA, GSHB, YFAY, UBIF
Annotation Cluster 18	Enrichment Score: 2.902722355774302	
Category	Term	Genes
SP_PIR_KEYWORDS	Lysine biosynthesis	LYSA, DAPB, ASD, DAPE, DAPD
SP_PIR_KEYWORDS	amino-acid biosynthesis	LYSA, DAPB, ARGE, ASD, HISC, THRC, AROD, GLTD, DAPE, DAPD, GLTB
Annotation Cluster 19	Enrichment Score: 2.8725715336880624	

Category	Term	Genes
KEGG_PATHWAY	ect00630:Glyoxylate and dicarboxylate metabolism	GLCF, ACNB, FDNG, FDNI, FUCO, FDNH, GLTA
Annotation Cluster 20	Enrichment Score: 2.7467466191310326	
Category	Term	Genes
SP_PIR_KEYWORDS	glycerol metabolism	GLPK, DHAL, DHAK, GLDA, GLPD
GOTERM_BP_FAT	GO:0006071~glycerol metabolic process	GLPK, GLPB, GLPA, GLPF, DHAL, DHAK, GLDA, GLPD, GLPC
Annotation Cluster 21	Enrichment Score: 2.731945431328348	
Category	Term	Genes
GOTERM_BP_FAT	GO:0006542~glutamine biosynthetic process	GLNG, GLNH, GLNQ, GLNA, GLNP, GLNL
GOTERM_BP_FAT	GO:0006541~glutamine metabolic process	GLNG, GLNH, GUAA, GLNQ, GLNA, GLNP, YAFJ, GLNL, GLTB
SP_PIR_KEYWORDS	amino-acid biosynthesis	LYSA, DAPB, ARGE, ASD, HISC, THRC, AROD, GLTD, DAPE, DAPD, GLTB
GOTERM_BP_FAT	GO:0044271~nitrogen compound biosynthetic process	LYSA, TDCC, ASD, THRC, YGIF, NAPA, MOAE, MOAD, MOAC, SGBH, MOAB, MOAA, GLNQ, ZNTA, GLTD, GLNP, GLNL, HEMN, YGGW, GLTB, GLNG, GLNH, EUTT, ARGE, CYSG, GLNA, MOEA, QUEF, DAPE, GLTP, DAPD, DAPB, FOLP, GUAA, BIOH, LYSR, HISC, GMHB, AROD, YFAY, HISJ
Annotation Cluster 22	Enrichment Score: 2.7306607420725055	
Category	Term	Genes
GOTERM_BP_FAT	GO:0015949~nucleobase, nucleoside and nucleotide interconversion	UDP, CPDB, APHA, GUAA, DGT, NUPG, DEOD, CDD, DEOC, DEOB, TSX, DEOA, NRDB
KEGG_PATHWAY	ect00230:Purine metabolism	NUDF, YFBR, GUAA, PPX, DGT, CPDB, DEOD, DEOB, NRDB
Annotation Cluster 23	Enrichment Score: 2.596674060349352	
Category	Term	Genes
SP_PIR_KEYWORDS	ATP	GLNG, PCK, PHNC, CLPX, ALAS, CCMA, MURD, MALK, GLNQ, CLPP, HSDR, GSHB, NIKD, NIKE, GLNL, PGK
SP_PIR_KEYWORDS	nickel	HYBF, HYBC, HYBD, NIKD, NIKE, NIKC, HYPA, NIKR, HYPB
KEGG_PATHWAY	eck02010:ABC transporters	YCJV, GLNH, FECE, FECD, MALF, PHNC, MALG, PHNE, MALE, CCMA, MALK, GLNQ, XYLH, GLNP, FECB, NIKD, FECC, NIKE, HISJ, NIKC
UP_SEQ_FEATURE	nucleotide phosphate-binding region:ATP	YCJV, MIAA, PCK, ENVZ, YHBJ, GLPK, MALK, GLNQ, NIKD, NIKE, NANK, PGK, GLNG, MAK, FECE, PHNC, RECB, CLPX, MURD, CCMA, GUAA, HFLB, HSDR, YEEZ, GSHB, EUTP, GLK

Annotation Cluster 24	Enrichment Score: 2.5314645033989565	
Category	Term	Genes
KEGG_PATHWAY	eum00260:Glycine, serine and threonine metabolism	ASD, GCVP, THRC, TDCG, GCVT, TDCB

Annotation Cluster 25	Enrichment Score: 2.4303369051233727	
Category	Term	Genes
SP_PIR_KEYWORDS	nucleotide-binding	YCJV, TDCD, CPDB, PCK, NARX, YHBJ, ALAS, GLPK, ATOS, MALK, ZNTA, HFLX, NIKD, NANK, NIKE, NARL, PGK, GLGC, EUTT, FECE, RECB, CLPX, MURD, ARCB, EVGS, CLPP, HSDR, GSHA, GSHB, EUTP, MIAA, YJIA, DHAL, ENVZ, HYPB, PPK, MOAB, MOAA, GLNQ, FTSZ, GLNL, GLNG, GLNE, YFBR, FUCK, MAK, GLNA, PHNC, CPXA, NADK, MUTL, CCMA, GUAA, HFLB, YEEZ, GLK
SP_PIR_KEYWORDS	atp-binding	MIAA, YCJV, TDCD, PCK, DHAL, NARX, ENVZ, YHBJ, ALAS, GLPK, ATOS, PPK, MALK, GLNQ, ZNTA, NIKD, NANK, NIKE, NARL, GLNL, PGK, GLNG, GLGC, EUTT, GLNE, FUCK, MAK, GLNA, FECE, CPXA, PHNC, RECB, NADK, CLPX, MUTL, GUAA, CCMA, MURD, ARCB, HFLB, EVGS, CLPP, HSDR, GSHA, YEEZ, GSHB, EUTP, GLK
GOTERM_MF_FAT	GO:0017076~purine nucleotide binding	YCJV, TDCD, PCK, NARX, YHBJ, ALAS, GLPK, ATOS, GOR, MALK, ZNTA, HFLX, GLTD, NIKD, NIKE, NANK, NARL, PGK, GLGC, EUTT, GLPA, AHPF, FECE, RECB, CLPX, YPDA, MURD, ARCB, EVGS, CLPP, UBIH, HSDR, GSHA, GSHB, EUTP, UBIF, MIAA, YJIA, DHAL, ENVZ, NDH, HYPB, PPK, MOAB, GRPE, MOAA, GLNQ, FTSZ, GLNL, GLNG, GLNE, FUCK, MAK, GLNA, PHNC, CPXA, NADK, NIRB, MUTL, CCMA, GUAA, HFLB, YEEZ, GLK
GOTERM_MF_FAT	GO:0000166~nucleotide binding	YCJV, LDHA, MAEB, TDCD, CPDB, PCK, NARX, YHBJ, ALAS, GLPK, ATOS, GOR, MALK, ZNTA, HFLX, GLTD, NIKD, NIKE, NANK, NARL, PGK, GLGC, EUTT, GLPA, AHPF, FECE, RECB, YPDA, CLPX, MURD, ARCB, EVGS, CLPP, GSHA, UBIH, HSDR, GSHB, EUTP, UBIF, MIAA, ASD, YJIA, DHAL, ENVZ, NDH, HYPB, YQCA, PPK, MOAB, GRPE, MOAA, GLNQ, FTSZ, LLDD, GLNL, GLNG, GLNE, YFBR, FUCK, MAK, GLNA, PHNC, CPXA, NADK, MUTL, NIRB, CCMA, GUAA, HFLB, YEEZ, GLK

Annotation Cluster 26	Enrichment Score: 2.4223054797333674	
Category	Term	Genes
SP_PIR_KEYWORDS	magnesium	GUDX, MIAA, PPX, MOEA, GLPF, APHA, NUDF, DGT, GUDD, ZNTA, ISPB, PTSA, GLMM, GSHB, YFAW, PGM, YBJI, PTRA
GOTERM_MF_FAT	GO:0000287~magnesium ion binding	GUDX, MIAA, PPX, MOEA, GLPF, APHA, NUDF, DGT, GUDD, ZNTA, PTSA, GSHB, GLMM, DEOB, YFAW, PGM, YBJI, PTRA
UP_SEQ_FEATURE	metal ion-binding site:Magnesium	GUDX, GUDD, ZNTA, GLPF, PTSA, GLMM, APHA, YFAW, PGM, YBJI

Annotation Cluster 27	Enrichment Score: 2.3937104636277664	
Category	Term	Genes

GOTERM_BP_FAT	GO:0009410~response to xenobiotic stimulus	KATG, ZNTA, AHPF, CPXA, CPXR, SODA, YFGD
GOTERM_BP_FAT	GO:0006805~xenobiotic metabolic process	KATG, ZNTA, AHPF, CPXA, CPXR, SODA, YFGD
GOTERM_MF_FAT	GO:0016209~antioxidant activity	GOR, KATG, AHPF, SODA

Annotation Cluster 28	Enrichment Score: 2.291653622013819	
Category	Term	Genes
GOTERM_BP_FAT	GO:0015949~nucleobase, nucleoside and nucleotide interconversion	UDP, CPDB, APHA, GUAA, DGT, NUPG, DEOD, CDD, DEOC, DEOB, TSX, DEOA, NRDB
KEGG_PATHWAY	ecd00240:Pyrimidine metabolism	UDP, YFBR, CPDB, DEOD, CDD, DEOA, NRDB

Annotation Cluster 29	Enrichment Score: 2.2302500165299324	
Category	Term	Genes
KEGG_PATHWAY	ecd00620:Pyruvate metabolism	TDCE, MAEB, LDHA, MGSA, PCK, FUCO, LLDD

Annotation Cluster 30	Enrichment Score: 2.1564085686536174	
Category	Term	Genes
SP_PIR_KEYWORDS	heme	NIRB, NRFB, NRFA, CYBC, KATG, NRFF, HYBB, NAPC, FDNI, NAPB, NARI
SP_PIR_KEYWORDS	chromoprotein	NRFB, NRFA, KATG, NAPC, NARI
INTERPRO	IPR011031:Multihaem cytochrome	NRFB, NRFA, NAPC, NAPB
GOTERM_MF_FAT	GO:0046906~tetrapyrrole binding	NIRB, NRFA, CYBC, KATG, NAPC

Annotation Cluster 31	Enrichment Score: 2.075966135949771	
Category	Term	Genes
SP_PIR_KEYWORDS	Fucose metabolism	FUCK, FUCI, FUCO, FUCU, FUCP, FUCA
GOTERM_BP_FAT	GO:0006004~fucose metabolic process	FUCK, FUCI, FUCO, FUCU, FUCP, FUCA
KEGG_PATHWAY	ecd00051:Fructose and mannose metabolism	FUCK, MAK, FUCI, FBAA, YFAW, FUCA

Annotation Cluster 32	Enrichment Score: 1.9858242210851904	
Category	Term	Genes
SP_PIR_KEYWORDS	phosphohistidine	ATOS, NPR, ARCB, GPMA, CPXA, NARX, ENVZ, GLNL
SP_PIR_KEYWORDS	phosphotransferase	GLPK, ATOS, TDCD, PTSN, ARCB, CPXA, NARX, ENVZ, GLK, GLNL, PGK
SP_PIR_KEYWORDS	two-component regulatory system	GLNG, EVGA, CPXA, NARX, ENVZ, YPDB, ATOS, ARCB, EVGS, BAER, QSEB, CPXR, OMPR, NARL, BASR, GLNL
SP_PIR_KEYWORDS	autophosphorylation	ATOS, ARCB, CPXA, NARX, ENVZ, GLNL

SP_PIR_KEYWORDS	kinase	TDCD, FUCK, MAK, RDOA, CPXA, DHAL, DHAK, NARX, NADK, YEII, ENVZ, YPDA, GLPK, ATOS, PPK, NPR, ARCB, PTSN, EVGS, PTSA, NANK, GLNL, GLK, PGK
SMART	SM00387:HATPase_c	MUTL, ATOS, ARCB, EVGS, CPXA, NARX, ENVZ, GLNL, YPDA
SMART	SM00388:HisKA	ATOS, ARCB, EVGS, CPXA, NARX, ENVZ, GLNL
INTERPRO	IPR003594:ATP-binding region, ATPase-like	MUTL, ATOS, ARCB, EVGS, CPXA, NARX, ENVZ, GLNL, YPDA

Annotation Cluster 33	Enrichment Score: 1.98318521499125	
Category	Term	Genes
SP_PIR_KEYWORDS	Flavoprotein	GLPB, GLPA, AHPF, GLPD, MDAB, NDH, YQCA, NIRB, GOR, UBIH, LLDD, GLTB, UBIF, YGCN
SP_PIR_KEYWORDS	FAD	NIRB, GOR, GLPA, AHPF, UBIH, GLPD, NDH, UBIF, GLTB, YGCN
GOTERM_MF_FAT	GO:0050662~coenzyme binding	LDHA, MAEB, ASD, GLPA, AHPF, MDAB, GCVH, NDH, YFCH, YQCA, SUCB, NIRB, YGBJ, GOR, GLTD, UBIH, YEEZ, LLDD, UBIF

Annotation Cluster 34	Enrichment Score: 1.8558134030136189	
Category	Term	Genes
UP_SEQ_FEATURE	nucleotide phosphate-binding region:NADP	MAEB, YAJO, DKGA, IDNO, TAS, YQHD, YDHF, YHDH

Annotation Cluster 35	Enrichment Score: 1.7977954534369145	
Category	Term	Genes
GOTERM_BP_FAT	GO:0006071~glycerol metabolic process	GLPK, GLPB, GLPA, GLPF, DHAL, DHAK, GLDA, GLPD, GLPC
KEGG_PATHWAY	ecg00564:Glycerophospholipid metabolism	GLPB, GLPA, GLPD, GLPC

Annotation Cluster 36	Enrichment Score: 1.7302510491041339	
Category	Term	Genes
KEGG_PATHWAY	eum00480:Glutathione metabolism	PEPD, GOR, GSHA, GSHB
GOTERM_BP_FAT	GO:0006790~sulfur metabolic process	BIOH, MOAD, ASD, GOR, GSHA, SSEA, GSHB

Annotation Cluster 37	Enrichment Score: 1.6563975505401778	
Category	Term	Genes
SP_PIR_KEYWORDS	ion transport	NANC, FECE, YIDK, FECD, FECR, OMPC, YFEO, YJCE, ZUPT, LAMB, FECA, FECB, FECC, TSX, CLCA, KEFB
SP_PIR_KEYWORDS	iron transport	FECE, FECD, FECA, FECR, FECB, FECC

Annotation Cluster 38	Enrichment Score: 1.6376679556962557	
Category	Term	Genes
INTERPRO	IPR012335:Thioredoxin fold	ARSC, YGHU, AHPF, YBBN, DIPZ, DSBA, YFFB, YFGD

Annotation Cluster 39	Enrichment Score: 1.533374935154952	
Category	Term	Genes
SP_PIR_KEYWORDS	cell wall biogenesis/degradation	FTSW, MURD, MTGA, MURG, AMPD, DACB, SLT
SP_PIR_KEYWORDS	cell shape	FTSW, MURD, YFGA, MTGA, MURG, DACB
GOTERM_BP_FAT	GO:0005976~polysaccharide metabolic process	GLGB, GLGC, MTGA, KDUI, RFFM, AMPD, DACB, GLGP, FTSW, GLGA, MURD, LPCA, MURG, GMHB, GLGX, GLMM, MALS, SLT, MALQ, RFFA, MALP
GOTERM_BP_FAT	GO:0007047~cell wall organization	FTSW, MURD, MTGA, MURG, AMPD, DACB, SLT, YPDA
GOTERM_BP_FAT	GO:0051301~cell division	FTSW, MURD, HFLB, MURG, FTSZ, DACB
GOTERM_BP_FAT	GO:0045229~external encapsulating structure organization	FTSW, MURD, MTGA, MURG, AMPD, DACB, SLT, YPDA

Annotation Cluster 40	Enrichment Score: 1.5315464238170997	
Category	Term	Genes
SP_PIR_KEYWORDS	carbon-carbon lyase	LYSA, PCK, UBIX, FBAA, NANA, GLTA

Annotation Cluster 41	Enrichment Score: 1.4684917999045133	
Category	Term	Genes
KEGG_PATHWAY	ecv03440:Homologous recombination	DNAT, RECB, PRIC, RECR

Annotation Cluster 42	Enrichment Score: 1.3745912320160147	
Category	Term	Genes
SP_PIR_KEYWORDS	Protease	PEPD, GSPO, PMBA, HYBD, TESA, HFLC, HFLB, HFLX, CLPP, BCSE, YBEY, HFLK, YFGC, IADA, PEPP, PTRA
GOTERM_MF_FAT	GO:0008233~peptidase activity	ARGE, PEPD, CPDA, GSPO, PMBA, HYBD, DAPE, DACB, TESA, HFLC, HFLB, BCSE, CLPP, HFLX, YBEY, HFLK, YFGC, IADA, PEPP, PTRA

Annotation Cluster 43	Enrichment Score: 1.3596916673585608	
Category	Term	Genes
KEGG_PATHWAY	eck00270:Cysteine and methionine metabolism	ASD, LUXS, TDCG, SSEA

Annotation Cluster 44	Enrichment Score: 1.336106875978563	
Category	Term	Genes

GOTERM_BP_FAT	GO:0015768~maltose transport	MALK, LAMB, MALE
GOTERM_BP_FAT	GO:0042946~glucoside transport	MALK, LAMB, MALE
GOTERM_BP_FAT	GO:0015766~disaccharide transport	MALK, LAMB, MALE
GOTERM_MF_FAT	GO:0051119~sugar transmembrane transporter activity	YCJV, NANT, XYLE, NPR, MALK, PTSN, LAMB, PTSA, MALE
