# Supplementary material for: Central role for RNase YbeY in Hfq-dependent and Hfq-independent small-RNA regulation in bacteria
Source: BMC Genomics. 2014 Feb 11;15:121. doi: 10.1186/1471-2164-15-121 (PMC3933206; doi:10.1186/1471-2164-15-121)
Supplement: Additional file 6: Table S6 — Functional clusters of genes expressing higher in ΔybeY vs. WT under HU. [file 1471-2164-15-121-S6.pdf]

Supplemental Table 6. Functional clusters of genes differentially up-regulated in *ΔybeY* vs. WT under HU

Annotation Cluster 1	Enrichment Score: 22.558010037413176	
Category	Term	Genes
SP_PIR_KEYWORDS	membrane	YABI, GUDP, YFIN, NANC, YOHD, YEER, YCJP, YOHO, YNHF, NANT, WZA, SMPA, EAMA, YPHD, EAMB, YFIB, MRCA, SETB, YQHA, PHOQ, YGBN, YFHM, YFHK, HYBC, YGDD, GNTU, ATPI, NUOH, RHTC, YEDI, YGDL, YHHQ, YEDA, DLD, YGCS, CSTA, YJFL, WECH, YFGO, YEGT, YQJA, NAGE, COBS, FRUA, YNFE, YNFF, YNFH, TATE, YLAC, YFGA, YEGH, LLDP, KGTP, YFGH, YDEE, YJHB, EMRD, YDEA, WZC, YCAD, YTFL, YFEZ, YAHG, YGFQ, YCAP, FLU, MDTP, YIHN, YGIH, ARSB, YIHO, NARQ, YEIB, YHBE, HOKE, YEAN, YEIH, YQEG, YFEO, PRC, MGTA, PROP, DINF, CDSA, YBGH, NLPD, YIGG, MDLB, GSPK, YNEN, CLD, TYRP, RSTB, XYLH, YDGC, AMID, YBGT, YRAJ, RTN, APBE, YFCJ, YRAQ, YNJF, YJEH, IDNT, YFCA, NFRA, YRBG, YDHJ, YFBV, DMSC, MACA, YQFA, MLTF, SHIA, YEBQ, YBAL, YEBN, YEBS, DACD, YEJM, ZNUB, SDHA, ZNUC, SDHC, SDHD, YIDX, MLTA, MLTB, CYST, MLTD, POTB, YFAV, FTSB, POTA, TISB

Annotation Cluster 2	Enrichment Score: 5.481608124094565	
Category	Term	Genes
KEGG_PATHWAY	ecq02020:Two-component system	RCSA, ATOB, NARP, YFHK, NARQ, PHOB, ARCA, RSTA, RSTB, YFHA, UHPA, PHOQ, FLHC, CSRA, FLHD
SP_PIR_KEYWORDS	phosphoprotein	RPSA, NARP, NARQ, YFHK, PHOB, PPHA, NAGE, WZC, ARCA, FRUA, RSTA, INFC, RSTB, YFHA, UHPA, MGTA, UGD, PHOQ

Annotation Cluster 3	Enrichment Score: 5.166545127942868	
Category	Term	Genes
SP_PIR_KEYWORDS	P-loop	GARK, UVRD, COBU, SRMB, MDLB, UUP, BISC, REP, OBGE, ZNUC, NFRA, RNE, YRBF, POTA, SBCC, GMK
UP_SEQ_FEATURE	nucleotide phosphate-binding region:ATP	COAE, UVRD, COBU, CMK, SRMB, MDLB, YGGC, REP, DHAR, ZNUC, PYRH, YFHA, PHOQ, RECQ, YEHX, YGFQ, NANK, YRBF, POTA, SBCC, GMK

Annotation Cluster 4	Enrichment Score: 4.076973525915364	
Category	Term	Genes
SP_PIR_KEYWORDS	metal-binding	GUDX, TYNA, EFEB, PPHA, YDHY, YNFE, YNFF, YEIH, YNFG, ALAS, MSRB, RLMN, SPEA, GUDD, BFR, HEMF, YPHC, MGTA, PHOQ, NANK, AEGA, YFGC, YGBM, PFLC, NORV, HEMN, YFHL, HYBC, GARL, PTSP, FTNA, HYFI, GUAD, DAPE, YAGT, YEAX, SDHC, SDHD, LIGA, HCAC, AMID, ALKB, YGFT, YFAU
SP_PIR_KEYWORDS	iron	EFEB, PPHA, YDHY, YNFE, YNFF, YNFG, RLMN, YIAY, BFR, AEGA, PFLC, HEMN, NORV, YFHL, HYBC, GARL, FTNA, HYFI, YAGT, YEAX, SDHC, SDHD, HCAC, ALKB, YGFT

Annotation Cluster 5	Enrichment Score: 3.681573654246584	
Category	Term	Genes
SP_PIR_KEYWORDS	dna repair	MUG, UVRD, LEXA, YEIH, MUTH, REP, PHR, LIGA, MUTT, RECQ, CHO, ALKB, SBCC
SP_PIR_KEYWORDS	dna replication	REP, SBCD, GSPB, UVRD, LIGA, LEXA, MUTT, SBCC

Annotation Cluster 6	Enrichment Score: 3.66002232739701	
Category	Term	Genes
KEGG_PATHWAY	ecd00360:Phenylalanine metabolism	TYNA, FEAB, HCAF, HCAC, HCAB, HCAD
GOTERM_MF_FAT	GO:0016708~oxidoreductase activity, acting on paired donors, with incorporation or reduction of molecular oxygen, NADH or NADPH as one donor, and incorporation of two atoms of oxygen into one donor	HCAF, HCAC, HCAD
GOTERM_BP_FAT	GO:0019439~aromatic compound catabolic process	HCAF, HCAC, HCAB, HCAD

Annotation Cluster 7	Enrichment Score: 3.6069836511677815	
Category	Term	Genes
SP_PIR_KEYWORDS	dna repair	MUG, UVRD, LEXA, YEIH, MUTH, REP, PHR, LIGA, MUTT, RECQ, CHO, ALKB, SBCC
SP_PIR_KEYWORDS	DNA damage	MUG, UVRD, PHR, LIGA, LEXA, MUTT, RECQ, CHO, ALKB, YEIH, MUTH
GOTERM_BP_FAT	GO:0006281~DNA repair	MUG, UVRD, YEES, LEXA, YEIH, RECE, THRL, YEDI, MUTH, REP, PHR, LIGA, MUTT, RECQ, CHO, ALKB, DINJ, DINF
GOTERM_BP_FAT	GO:0033554~cellular response to stress	MUG, UVRD, YEES, LEXA, YEIH, RECE, THRL, YEDI, MUTH, REP, PHR, LIGA, MUTT, RECQ, CHO, CSTA, ALKB, DINJ, DINF
GOTERM_BP_FAT	GO:0006259~DNA metabolic process	YEES, INTD, YEIH, THRL, MUTH, REP, GSPB, INSO, RECQ, CHO, YRDD, DINF, SBCD, XSEA, MUG, YFDO, UVRD, LEXA, RECE, YEDI, LIGA, PHR, MUTT, REND, INTR, TORI, ALKB, DINJ, SBCC

Annotation Cluster 8	Enrichment Score: 3.56884019745853

Category	Term	Genes
SP_PIR_KEYWORDS	DNA binding	RCSA, NARP, YIAJ, CHPS, UVRD, PHOB, LEXA, CYSB, GALR, CSPF, ENVY, ARCA, RSTA, REP, YCAN, RPOS, PHR, CHPB, FLHC, MARR, ASCG, FLHD
SP_PIR_KEYWORDS	activator	RCSA, NARP, AAER, GCVA, PHOB, CSPF, ENVY, CYSB, ARCA, DHAR, UHPA, YDEO, XAPR, FLHC, EUTR, FLHD
SP_PIR_KEYWORDS	dna-binding	NARP, CHPS, CDAR, PHOB, CSPF, YEAM, YGBI, YFIE, RECQ, FLHC, XAPR, FLHD, RCSA, AAER, UVRD, LEXA, ARCA, GATR, RSTA, XYLH, YFHA, CHPB, EUTR, YFHH, ASCG, YEGW, ENVY, YGGD, REP, OBGE, DHAR, YJIR, UHPA, RPOS, RCNR, MARR, MUG, YIAJ, GCVA, YBAO, IDNR, CYSB, GALR, YHAJ, YGFI, YCAN, PHR, TORI, YDEO
SP_PIR_KEYWORDS	Transcription	NARP, YEGW, CDAR, PHOB, ENVY, CSPF, YEAM, YGBI, DHAR, UHPA, YJIR, RPOS, YFIE, XAPR, FLHC, RCNR, RPOZ, MARR, FLHD, RCSA, YIAJ, AAER, GCVA, YBAO, LEXA, IDNR, CYSB, GALR, ARCA, GATR, YHAJ, RSTA, YGFI, YCAN, YFHA, TORI, PCNB, YDEO, EUTR, YFHH, ASCG
SP_PIR_KEYWORDS	transcription regulation	NARP, YEGW, CDAR, PHOB, ENVY, CSPF, YEAM, YGBI, DHAR, UHPA, YJIR, RPOS, YFIE, XAPR, FLHC, RCNR, MARR, FLHD, RCSA, YIAJ, AAER, GCVA, YBAO, LEXA, IDNR, CYSB, GALR, ARCA, GATR, YHAJ, RSTA, YGFI, YCAN, YFHA, TORI, YDEO, MLTD, EUTR, YFHH, ASCG
INTERPRO	IPR011991:Winged helix repressor DNA-binding	RCSA, NARP, YEGW, AAER, GCVA, YBAO, PHOB, LEXA, CYSB, YHAJ, YQEH, RSTA, REP, YGFI, YCAN, UHPA, YJIR, RPOS, YFIE, XAPR, MARR
GOTERM_BP_FAT	GO:0006350~transcription	NARP, YEGW, CDAR, PHOB, ENVY, CSPF, YEAM, YGBI, DHAR, UHPA, YJIR, RPOS, YFIE, XAPR, FLHC, RCNR, RPOZ, MARR, FLHD, RCSA, YIAJ, AAER, GCVA, YBAO, LEXA, IDNR, CYSB, GALR, ARCA, GATR, YHAJ, RSTA, YGFI, YCAN, YFHA, TORI, PCNB, YDEO, MLTD, EUTR, YFHH, ASCG
GOTERM_BP_FAT	GO:0045449~regulation of transcription	NARP, YEGW, CDAR, PHOB, ENVY, CSPF, YEAM, THRL, YQEH, YGBI, DHAR, UHPA, YJIR, RPOS, YFIE, XAPR, FLHC, RCNR, MARR, FLHD, RCSA, YIAJ, AAER, GCVA, YBAO, LEXA, IDNR, CYSB, GALR, ARCA, GATR, YHAJ, RSTA, YGFI, YCAN, YFHA, TORI, YDEO, EUTR, YFHH, ASCG
GOTERM_MF_FAT	GO:0003677~DNA binding	NARP, CDAR, CHPS, PHOB, CSPF, YEIH, INTD, YEAM, YQEH, YGBI, INSO, YFIE, RECQ, YRDD, XAPR, FLHC, FLHD, RCSA, AAER, UVRD, LEXA, ARCA, YEDI, GATR, RSTA, YFHA, XYLH, LIGA, INTR, CHPB, EUTR, YFHH, ASCG, YRAN, YEGW, ENVY, YGGD, MUTH, REP, OBGE, YFGA, DHAR, UHPA, YJIR, RPOS, RPOZ, RCNR, MARR, YIAJ, MUG, GCVA, YBAO, IDNR, GALR, YEJH, CYSB, YHAJ, YGFI, YCAN, PHR, REND, TORI, YDEO

Annotation Cluster 9	Enrichment Score: 3.4583647369026926	
Category	Term	Genes
SP_PIR_KEYWORDS	leader peptide	IVBL, ILVL, THRL, HISL
GOTERM_BP_FAT	GO:0009088~threonine biosynthetic process	RHTC, THRL
SP_PIR_KEYWORDS	amino-acid biosynthesis	IVBL, ILVL, AROE, CYSB, DAPE, THRL, HISL

GOTERM_BP_FAT	GO:0009067~aspartate family amino acid biosynthetic process	CADA, RHTC, DAPE, THRL

Annotation Cluster 10	Enrichment Score: 2.593006103656498	
Category	Term	Genes
GOTERM_BP_FAT	GO:0009309~amine biosynthetic process	IVBL, CADA, ILVL, CYSB, RHTC, DAPE, YBCF, TYRP, THRL, SPEC, PYRH, SPEA, AROE, PROP, AEGA, POTB, POTA, YGFT, HISL
GOTERM_BP_FAT	GO:0044271~nitrogen compound biosynthetic process	YAFE, CADA, ILVL, COBU, APBE, COBS, THRL, BISC, SPEC, SPEA, HEMF, MGTA, PROP, AEGA, HEMN, GMK, FEAB, IVBL, ATPI, CYSB, RHTC, DAPE, TYRP, YBCF, YAGT, PYRH, AROE, POTB, POTA, HISL, YGFT
GOTERM_BP_FAT	GO:0046394~carboxylic acid biosynthetic process	YAFE, IVBL, SHIA, CADA, ILVL, CYSB, RHTC, DAPE, YBCF, TYRP, THRL, BISC, PYRH, AROE, PROP, AEGA, YGFT, HISL

Annotation Cluster 11	Enrichment Score: 2.4306200666268634	
Category	Term	Genes
SP_PIR_KEYWORDS	nad	FEAB, HCAF, GARR, NUOH, YEAX, GHRA, YGBJ, YIAY, UGD, DLD, LIGA, AROE, HCAB, GLXR, HCAD, NORW, NUDC, FADJ, EPD
INTERPRO	IPR016040:NAD(P)-binding domain	YJHC, YBJT, GARR, YBAL, YGFF, YGDL, GHRA, YGBJ, FOLM, UGD, AROE, HCAB, GLXR, AEGA, EPD, FADJ, YGFT
GOTERM_MF_FAT	GO:0048037~cofactor binding	TYNA, CADA, YGCU, YBJT, GARR, HYFI, NUOH, SDHA, SPEC, GHRA, YGBJ, YJIR, UGD, DLD, AROE, GLXR, YFDZ, HCAD, AEGA, NORW, NORV, EPD, FADJ, YGFT

Annotation Cluster 12	Enrichment Score: 2.299408202652713	
Category	Term	Genes
KEGG_PATHWAY	ecj00071:Fatty acid metabolism	ATOB, YIAY, HCAD, FADJ, FADI
KEGG_PATHWAY	eum00280:Valine, leucine and isoleucine degradation	ATOB, FADJ, FADI

Annotation Cluster 13	Enrichment Score: 2.2973457044037477	
Category	Term	Genes
KEGG_PATHWAY	eum00632:Benzoate degradation via CoA ligation	SDHA, ATOB, SDHC, SDHD, YCCX, FADJ

SP_PIR_KEYWORDS	electron transport	SDHA, YEAX, SDHC, SDHD, HCAC, YGCR, YNFG, NORV
KEGG_PATHWAY	ecd00190:Oxidative phosphorylation	SDHA, SDHC, SDHD, NUOH
KEGG_PATHWAY	ecd00020:Citrate cycle (TCA cycle)	SDHA, SDHC, SDHD
KEGG_PATHWAY	ecy00190:Oxidative phosphorylation	SDHA, SDHC, SDHD
GOTERM_BP_FAT	GO:0009060~aerobic respiration	SDHA, SDHC, DLD, SDHD, NUOH
GOTERM_BP_FAT	GO:0006084~acetyl-CoA metabolic process	SDHA, SDHC, SDHD, SCPC

Annotation Cluster 14	Enrichment Score: 2.196229016581442	
Category	Term	Genes
KEGG_PATHWAY	ecv03430:Mismatch repair	XSEA, UVRD, LIGA, MUTH

Annotation Cluster 15	Enrichment Score: 2.1750395605481305	
Category	Term	Genes
SP_PIR_KEYWORDS	cell wall biogenesis/degradation	MLTF, MRCA, MLTA, AMID, AMIC, MLTB, MLTD, AMIA, DACB, DACD
GOTERM_BP_FAT	GO:0045229~external encapsulating structure organization	MLTF, YRAI, YCBF, WZC, DACB, DACD, MRCA, MLTA, AMID, AMIC, MLTB, MLTD, AMIA, YQIH, YCBR
GOTERM_BP_FAT	GO:0007047~cell wall organization	MLTF, YRAI, YCBF, DACB, DACD, MRCA, MLTA, AMID, AMIC, MLTB, MLTD, AMIA, YQIH, YCBR
GOTERM_BP_FAT	GO:0000271~polysaccharide biosynthetic process	RCSA, KDSC, WCAA, WZC, WCAB, CLD, DACB, DACD, WZA, WZB, PRC, UGD, MRCA, MLTA, ASMA, AMIC, MLTB, AMIA
GOTERM_BP_FAT	GO:0005976~polysaccharide metabolic process	RCSA, YEGX, KDSC, WCAA, WZC, WCAB, CLD, DACB, DACD, WZA, WZB, PRC, UGD, MRCA, MLTA, AMID, ASMA, AMIC, MLTB, AMIA
SP_PIR_KEYWORDS	cell shape	YFGA, MRCA, DACB, DACD
KEGG_PATHWAY	ecd00550:Peptidoglycan biosynthesis	MRCA, DACB, DACD
GOTERM_BP_FAT	GO:0008360~regulation of cell shape	YFGA, MRCA, DACB, DACD

Annotation Cluster 16	Enrichment Score: 2.136664960213052	

Category	Term	Genes
KEGG_PATHWAY	ecr00053:Ascorbate and aldarate metabolism	GUDX, GUDD, UGD, GARL
GOTERM_BP_FAT	GO:0016054~organic acid catabolic process	GUDX, SPEA, GUDD, GARL, ARCA, THRL, FADJ
GOTERM_BP_FAT	GO:0046395~carboxylic acid catabolic process	GUDX, SPEA, GUDD, GARL, ARCA, THRL, FADJ

Annotation Cluster 17	Enrichment Score: 2.1055151957596534	
Category	Term	Genes
SP_PIR_KEYWORDS	palmitate	YFHM, MDTP, NLPD, APBE, WZA, SMPA, MGRB, YFIB, AMID, MLTA, MLTB, MLTD, YFGH
SP_PIR_KEYWORDS	lipoprotein	YFHM, MDTP, NLPD, DEDD, APBE, WZA, SMPA, MGRB, YFIB, YIDX, MLTA, AMID, MLTB, MLTD, YFGH
GOTERM_CC_FAT	GO:0031225~anchored to membrane	YFHM, MDTP, SMPA, NLPD, YFIB, MLTA, APBE, AMID, YFGH, MLTD
UP_SEQ_FEATURE	lipid moiety-binding region:N-palmitoyl cysteine	YFHM, MDTP, NLPD, APBE, WZA, SMPA, MGRB, YFIB, AMID, MLTA, MLTB, MLTD, YFGH
UP_SEQ_FEATURE	lipid moiety-binding region:S-diacylglycerol cysteine	YFHM, MDTP, NLPD, APBE, WZA, SMPA, MGRB, YFIB, AMID, MLTA, MLTB, MLTD, YFGH
GOTERM_CC_FAT	GO:0009279~cell outer membrane	MDTP, NANC, MLTF, YRAJ, ENVY, WZA, SMPA, YFIB, YTFM, MLTA, NFRA, AMID, MLTB, FLU, YFGH
GOTERM_CC_FAT	GO:0019867~outer membrane	MDTP, NANC, MLTF, YRAJ, ENVY, WZA, SMPA, YFAL, YTFM, YFIB, MLTA, NFRA, AMID, MLTB, FLU, YFGH

Annotation Cluster 18	Enrichment Score: 2.0381646171219305	
Category	Term	Genes
GOTERM_BP_FAT	GO:0009242~colanic acid biosynthetic process	RCSA, WZA, WZB, UGD, WCAA, WZC, WCAB
GOTERM_BP_FAT	GO:0016051~carbohydrate biosynthetic process	NANE, NAGE, NANA, WZA, NANT, WZB, PRC, MRCA, UGD, NANK, RCSA, WCAA, KDSC, WCAB, WZC, CLD, DACB, DACD, ASMA, MLTA, AMIC, MLTB, AMIA, CSRA, EPD
GOTERM_BP_FAT	GO:0000271~polysaccharide biosynthetic process	RCSA, KDSC, WCAA, WZC, WCAB, CLD, DACB, DACD, WZA, WZB, PRC, UGD, MRCA, MLTA, ASMA, AMIC, MLTB, AMIA

GOTERM_MF_FAT	GO:0042280~cell surface antigen activity, host-interacting	RCSA, UGD, ASMA, NANA, CLD
GOTERM_BP_FAT	GO:0005976~polysaccharide metabolic process	RCSA, YEGX, KDSC, WCAA, WZC, WCAB, CLD, DACB, DACD, WZA, WZB, PRC, UGD, MRCA, MLTA, AMID, ASMA, AMIC, MLTB, AMIA
SP_PIR_KEYWORDS	lipopolysaccharide biosynthesis	KDSC, WCAA, WCAB, CLD

Annotation Cluster 19	Enrichment Score: 1.9931258934494718	
Category	Term	Genes
KEGG_PATHWAY	eum00310:Lysine degradation	ATOB, CADA, FADJ

Annotation Cluster 20	Enrichment Score: 1.955691199999416	
Category	Term	Genes
GOTERM_BP_FAT	GO:0015949~nucleobase, nucleoside and nucleotide interconversion	PYRH, CMK, XAPR, XAPA, SPOT, GMK

Annotation Cluster 21	Enrichment Score: 1.867920143569332	
Category	Term	Genes
SP_PIR_KEYWORDS	electron transport	SDHA, YEAX, SDHC, SDHD, HCAC, YGCR, YNFG, NORV
GOTERM_BP_FAT	GO:0006091~generation of precursor metabolites and energy	NARP, NARQ, HYBC, ATPI, HYFI, NUOH, YNFG, YNFH, SDHA, YEAX, SDHC, MGTA, SDHD, DLD, HCAC, YGCR, MLTD, PFLC, CSRA, NORV, DMSC
GOTERM_BP_FAT	GO:0022900~electron transport chain	SDHA, YEAX, SDHC, SDHD, HCAC, YGCR, YNFG, NORV, DMSC, YNFH

Annotation Cluster 22	Enrichment Score: 1.8638896700960417	
Category	Term	Genes
SP_PIR_KEYWORDS	magnesium	GUDX, GARL, PTSP, KDSC, NUDG, SPEA, GUDD, ISPD, MGTA, LIGA, PHOQ, MUTT, NUDC, GMK, YFAU

Annotation Cluster 23	Enrichment Score: 1.8579286992190174	
Category	Term	Genes

GOTERM_BP_FAT	GO:0009061~anaerobic respiration	NARP, NARQ, HYBC, HYFI, MLTD, NUOH, PFLC, DMSC, YNFH
GOTERM_BP_FAT	GO:0045333~cellular respiration	NARP, NARQ, HYBC, HYFI, NUOH, YNFH, SDHA, SDHC, SDHD, DLD, MLTD, PFLC, DMSC
GOTERM_BP_FAT	GO:0006091~generation of precursor metabolites and energy	NARP, NARQ, HYBC, ATPI, HYFI, NUOH, YNFG, YNFH, SDHA, YEAX, SDHC, MGTA, SDHD, DLD, HCAC, YGCR, MLTD, PFLC, CSRA, NORV, DMSC

Annotation Cluster 24	Enrichment Score: 1.8566558223268543	
Category	Term	Genes
SP_PIR_KEYWORDS	iron-sulfur protein	YAGT, YNFE, YNFF, YNFG
INTERPRO	IPR017909:Twin arginine translocation signal, Tat	YAGT, EFEB, AMIC, AMIA, YNFE, YNFF
INTERPRO	IPR006311:Twin-arginine translocation pathway signal	YAGT, EFEB, AMIC, AMIA, YNFE, YNFF

Annotation Cluster 25	Enrichment Score: 1.8406693287771927	
Category	Term	Genes
KEGG_PATHWAY	ecd00860:Porphyrin and chlorophyll metabolism	HEMF, COBU, COBS, HEMN
GOTERM_BP_FAT	GO:0018130~heterocycle biosynthetic process	YAGT, YAFE, HEMF, COBU, APBE, PROP, COBS, HEMN, HISL, BISC
GOTERM_BP_FAT	GO:0051188~cofactor biosynthetic process	FEAB, COAE, HEMF, COBU, YGGC, COBS, EPD, HEMN

Annotation Cluster 26	Enrichment Score: 1.8045263666454574	
Category	Term	Genes
SP_PIR_KEYWORDS	sos response	UVRD, LEXA, RECQ, CHO
GOTERM_BP_FAT	GO:0009991~response to extracellular stimulus	UVRD, LEXA, RECQ, CHO, CSTA, DINF, SPOT
GOTERM_BP_FAT	GO:0009432~SOS response	UVRD, LEXA, RECQ, CHO, DINF
GOTERM_BP_FAT	GO:0031668~cellular response to extracellular stimulus	UVRD, LEXA, RECQ, CHO, CSTA, DINF

Annotation Cluster 27	Enrichment Score: 1.7573895430205617	
Category	Term	Genes
KEGG_PATHWAY	ecg00630:Glyoxylate and dicarboxylate metabolism	GARK, GHRA, GARR, GLXR, HYI

Annotation Cluster 28	Enrichment Score: 1.7332313998861009	
Category	Term	Genes
SP_PIR_KEYWORDS	gluconate utilization	IDNT, GNTU, IDNR
GOTERM_BP_FAT	GO:0019520~aldonic acid metabolic process	IDNT, GNTU, IDNR
GOTERM_BP_FAT	GO:0019521~D-gluconate metabolic process	IDNT, GNTU, IDNR

Annotation Cluster 29	Enrichment Score: 1.7240255946878682	
Category	Term	Genes
SP_PIR_KEYWORDS	nuclease	SBCD, XSEA, RNE, YEIH, RECE, SBCC, MUTH
GOTERM_BP_FAT	GO:0009057~macromolecule catabolic process	SBCD, XSEA, MUG, YEGX, AMID, RNE, AMIC, AMIA, CSRA, RECE, SBCC
SP_PIR_KEYWORDS	Endonuclease	SBCD, RNE, CHO, YEIH, THRL, SBCC, MUTH
SP_PIR_KEYWORDS	exonuclease	SBCD, XSEA, RECE, SBCC
GOTERM_MF_FAT	GO:0004518~nuclease activity	SBCD, XSEA, RNE, CHO, YEIH, THRL, RECE, SBCC, MUTH

Annotation Cluster 30	Enrichment Score: 1.7174443313132048	
Category	Term	Genes
KEGG_PATHWAY	ecg03018:RNA degradation	RNE, PCNB, RECQ

Annotation Cluster 31	Enrichment Score: 1.6978773216516245	
Category	Term	Genes
SP_PIR_KEYWORDS	dioxygenase	YEAX, HCAF, HCAC, ALKB

GOTERM_MF_FAT	GO:0016702~oxidoreductase activity, acting on single donors with incorporation of molecular oxygen, incorporation of two atoms of oxygen	YEAX, HCAF, HCAC, ALKB

Annotation Cluster 32	Enrichment Score: 1.6339792435832559	
Category	Term	Genes
GOTERM_BP_FAT	GO:0046349~amino sugar biosynthetic process	NANE, NANT, NAGE, NANK, NANA
GOTERM_BP_FAT	GO:0046364~monosaccharide biosynthetic process	NANE, NANT, NAGE, NANK, NANA, CSRA, EPD
KEGG_PATHWAY	ecd00520:Amino sugar and nucleotide sugar metabolism	NANE, UGD, NAGE, NANK, NANA
GOTERM_BP_FAT	GO:0005996~monosaccharide metabolic process	NANE, GARR, GALR, NAGE, NANA, YIHS, NANT, YGBJ, GLXR, NANK, CSRA, PFLC, EPD

Annotation Cluster 33	Enrichment Score: 1.6006178952648982	
Category	Term	Genes
SP_PIR_KEYWORDS	Symport	YIHO, SHIA, PROP, KGTP
INTERPRO	IPR005829:Sugar transporter, conserved site	NANT, SHIA, YJHB, YGCS, PROP, KGTP
INTERPRO	IPR005828:General substrate transporter	SHIA, YJHB, PROP, KGTP
INTERPRO	IPR004736:Citrate-proton symport	SHIA, PROP, KGTP
PIR_SUPERFAMILY	PIRSF003274:citrate utilization determinant	SHIA, PROP, KGTP

Annotation Cluster 34	Enrichment Score: 1.5642268962258008	
Category	Term	Genes

GOTERM_BP_FAT	GO:0015949~nucleobase, nucleoside and nucleotide interconversion	PYRH, CMK, XAPR, XAPA, SPOT, GMK
KEGG_PATHWAY	ecd00230:Purine metabolism	GUAD, YBCF, RPOZ, XAPA, SPOT, GMK

Annotation Cluster 35	Enrichment Score: 1.532127508373884	
Category	Term	Genes
SP_PIR_KEYWORDS	s-adenosyl-l-methionine	RLMN, TRMA, YFIC, YGDE, TRMI, CMOB, PFLC, HEMN
SP_PIR_KEYWORDS	methyltransferase	YAFE, RLMN, TRMA, YFIC, YGDE, ALKB, TRMI, CMOB, YGBN, YBCY
GOTERM_MF_FAT	GO:0008173~RNA methyltransferase activity	RLMN, TRMA, TRMI, CMOB

Annotation Cluster 36	Enrichment Score: 1.4593204053832587	
Category	Term	Genes
SP_PIR_KEYWORDS	nucleotide-binding	NARP, NARQ, SRMB, COBU, YGGC, YEAP, ALAS, REP, OBGE, DHAR, MGTA, PHOQ, RECQ, NANK, YRBF, RTCA, GMK, GARK, YFHK, YFJK, COAE, UVRD, CMK, MDLB, YEJH, WZC, UUP, YBCF, RSTB, ZNUC, PYRH, YFHA, PHR, PCNB, YEHX, YGFQ, POTA, YFJP, SBCC
SP_PIR_KEYWORDS	atp-binding	NARP, NARQ, SRMB, COBU, YGGC, ALAS, REP, DHAR, MGTA, PHOQ, RECQ, NANK, YRBF, RTCA, GMK, GARK, YFHK, YFJK, COAE, UVRD, CMK, MDLB, YEJH, WZC, UUP, YBCF, RSTB, ZNUC, PYRH, YFHA, YFAL, PCNB, YEHX, YGFQ, POTA, SBCC
GOTERM_MF_FAT	GO:0017076~purine nucleotide binding	NARP, NARQ, SRMB, COBU, YGGC, YEAP, ALAS, REP, OBGE, DHAR, MGTA, PHOQ, RECQ, NANK, YRBF, AEGA, NORW, RTCA, GMK, GARK, YFJK, YFHK, COAE, UVRD, YGCU, CMK, MDLB, YEJH, WZC, UUP, YBCF, YEDI, ZNUB, SDHA, RSTB, ZNUC, PYRH, YFHA, YFAL, DLD, YEHX, PCNB, YGFQ, HCAD, POTA, YFJP, YGFT, SBCC
GOTERM_MF_FAT	GO:0001882~nucleoside binding	NARP, NARQ, SRMB, COBU, YGGC, ALAS, REP, DHAR, MGTA, PHOQ, RECQ, NANK, YRBF, AEGA, NORW, RTCA, GMK, GARK, YFHK, YFJK, COAE, UVRD, YGCU, CMK, MDLB, YEJH, WZC, UUP, YBCF, YEDI, ZNUB, SDHA, RSTB, ZNUC, PYRH, YFHA, YFAL, DLD, YEHX, PCNB, YGFQ, HCAD, POTA, YGFT, SBCC
GOTERM_MF_FAT	GO:0000166~nucleotide binding	NARP, NARQ, SRMB, YEAP, ALAS, MGTA, UGD, PHOQ, RECQ, NANK, NORW, NORV, YFHK, UVRD, YGCU, MDLB, YBCF, UUP, YEDI, RSTB, PYRH, YFHA, DLD, AROE, YEHX, EPD, COBU, YGGC, REP, OBGE, DHAR, AEGA, YRBF, RTCA, FADJ, GMK, GARK, YFJK, COAE, CMK, YEJH, WZC, SDHA, ZNUB, ZNUC, YFAL, PHR, PCNB, YGFQ, HCAD, POTA, YFJP, SBCC, YGFT

Annotation Cluster 37	Enrichment Score: 1.3673289282575685	
Category	Term	Genes

SP_PIR_KEYWORDS	iron	EFEB, PPHA, YDHY, YNFE, YNFF, YNFG, RLMN, YIAY, BFR, AEGA, PFLC, HEMN, NORV, YFHL, HYBC, GARL, FTNA, HYFI, YAGT, YEAX, SDHC, SDHD, HCAC, ALKB, YGFT
SP_PIR_KEYWORDS	iron-sulfur	YFHL, YDHY, HYFI, YNFE, YNFF, YNFG, YAGT, YEAX, RLMN, HCAC, AEGA, PFLC, HEMN, YGFT
SP_PIR_KEYWORDS	4fe-4s	RLMN, YFHL, YDHY, HYFI, AEGA, YNFE, YNFF, PFLC, YNFG, HEMN, YGFT
GOTERM_MF_FAT	GO:0005506~iron ion binding	YFHL, GARL, EFEB, PPHA, FTNA, YDHY, HYFI, YNFE, YNFF, YNFG, YAGT, YEAX, RLMN, YIAY, BFR, SDHC, SDHD, HCAC, AEGA, ALKB, PFLC, NORV, HEMN, YGFT
GOTERM_MF_FAT	GO:0009055~electron carrier activity	YJHC, YFHL, YDHY, YNFE, YNFF, YNFG, BISC, SDHA, YAGT, YEAX, SDHC, HCAC, AEGA, HCAD, YGCR, NORW, NORV, YGFT

Annotation Cluster 38	Enrichment Score: 1.3600012340233274	
Category	Term	Genes
SP_PIR_KEYWORDS	Flavoprotein	SDHA, YEAX, YGCU, DLD, PHR, YGCR, HCAD, NORW, NORV
SP_PIR_KEYWORDS	FAD	SDHA, YGCU, DLD, PHR, YGCR, HCAD, NORW
