# Supplementary material for: Central role for RNase YbeY in Hfq-dependent and Hfq-independent small-RNA regulation in bacteria
Source: BMC Genomics. 2014 Feb 11;15:121. doi: 10.1186/1471-2164-15-121 (PMC3933206; doi:10.1186/1471-2164-15-121)
Supplement: Additional file 7: Table S7 — Functional clusters of genes showing significantly lower expression in ΔybeY vs. WT under HU. [file 1471-2164-15-121-S7.pdf]

Supplemental Table 7. Functional clusters of genes differentially down-regulated in *ΔybeY* vs. WT under HU

Annotation Cluster 1	Enrichment Score: 18.267660213706964	
Category	Term	Genes
SP_PIR_KEYWORDS	metal-binding	MAEB, ENO, HYCI, MAEA, CPDB, CRR, XTHA, GLDA, FUMB, GLPK, HEMB, NRFC, NARH, PRLC, NARG, RECR, NARI, MALS, YQHD, PGM, SLYD, CUTA, GLCF, PURF, PEPD, MELA, MOEA, HMP, YCGM, YSAA, HYFG, NIKR, PURA, RUMA, YCCM, SUCC, GSHB, RIBA, YGCO, PEPQ, YBJI, PFKA, YJJW, PURT, USHA, NAPG, MANA, NAPH, GPMM, NFUA, NAPF, NAPC, HYPD, CCA, NAPA, NAPB, AMPD, GLOA, HYPA, HYPB, PTSI, SPEB, YFCE, DCP, PTSA, NRDF, YJES, RNHA, CDD, PPA, YIDA, BTUB, HCR, HCP, NRFA, PYKF, FUCI, HPT, GALT, FBP, DDLA, NIRB, YBIY, PQQL, TDH, DKSA, YBEY, GLMM, DEOB, SODA, CCME, SODC
GOTERM_MF_FAT	GO:0043169~cation binding	MAEB, ENO, HYCI, UPP, MAEA, GLDA, EUTC, FUMB, FECA, PRLC, RECR, NIKD, FECB, NIKE, FECC, ZUR, MALS, NIKB, NIKC, PGM, YQHD, MALQ, NIKA, CUTA, ENTF, GLCF, FECI, FECE, FECD, FECR, KDSB, YSAA, ENTB, NIKR, SUCC, YCCM, RIBA, YGCO, YBJI, LNT, PURT, MIAA, NAPG, MANA, GPMM, NAPH, NFUA, NAPF, NAPC, NAPA, NAPB, GLOA, YQCA, KDPA, PTSI, PTSA, NRDF, CDD, PPA, BTUB, NRFA, PYKF, GALT, FTNB, NIRB, MOBA, TDH, YBEY, GLMM, DEOB, CCME, FHUC, FHUD, CRR, CPDB, PCK, XTHA, GLPK, HEMB, NRFC, NARH, NARG, DXS, NARI, GLPX, SLYD, FHUB, FHUA, YIIM, PURF, PEPD, NLPE, MELA, MOEA, FEOB, HMP, FEOA, YCGM, HYFG, PURA, RUMA, GLGX, GSHB, ADE, PEPQ, HSLO, PFKA, YJJW, USHA, HYPD, CCA, AMPD, HYPA, HYPB, YHDH, SPEB, UXAA, YFCE, DCP, YJES, RNHA, YIDA, HCR, HCP, FUCI, YNCB, YHJJ, HPT, NADK, FBP, DDLA, PQQL, YBIY, DKSA, ZRAS, BGLA, SODA, SODC

Annotation Cluster 2	Enrichment Score: 14.621421866464393	
Category	Term	Genes
GOTERM_BP_FAT	GO:0009061~anaerobic respiration	YJJW, ENO, FRDC, NAPG, NAPH, NAPF, HYPC, NAPC, HYPD, NARX, NAPD, HYPE, NAPA, NAPB, FUMB, HYPA, NRFC, NARH, NARG, NIKD, NARJ, NARI, NIKE, NARL, MENB, NRFA, PYKF, LPD, HYFG, NIRB, TORR, YBIY, NIRD, UBIE
GOTERM_BP_FAT	GO:0006091~generation of precursor metabolites and energy	LDHA, ENO, FRDC, NARX, FUMB, NRFC, NARH, NARG, NIKD, NARJ, NARI, NIKE, NARL, GLCF, GLPD, YSAA, HYFG, SUCB, YCCM, SUCC, ARCB, GPMA, GLGX, UBIE, YGCO, PFKA, YJJW, TPIA, NAPG, GPMM, NAPH, NAPF, HYPC, NAPC, HYPD, NAPD, NAPA, HYPE, NAPB, HYPA, HYPB, YQCA, GLGA, FHLA, PPC, MENB, HCR, NRFA, PYKF, LPD, DSBB, NIRB, TORR, GRXB, GPSA, GRXC, GRXD, YBIY, NIRD, ZRAS

Annotation Cluster 3	Enrichment Score: 14.206932936809334	
Category	Term	Genes

SP_PIR_KEYWORDS	cell inner membrane	YGAZ, HTPG, YOHJ, MALF, MALG, HDED, PSTB, GLTK, FABI, ATOS, GLTL, MALK, YBBM, NIKD, FECC, NIKE, NIKB, NIKC, YBBY, GNTP, MALX, FECE, FECD, SYD, WBBK, YCCM, NUPG, NUPC, YBJM, YDCO, BASS, LNT, YDDG, NAPH, NAPC, KDPA, DDPB, GLNQ, YRFF, GALP, GLNP, MANY, MANZ, YIAD, LPD, CCMC, CCMD, CCMA, CCMB, CYNX, SAPB, NIRC, YAAJ, EXBD, EXBB, CCME, FHUC, YEAI, FRDC, DCUC, DCUB, NEPI, NARX, TQSA, NARI, MDTF, NARK, MDTL, FHUB, FEOB, HEMX, YPDA, YIHY, YHCB, ARCB, YHBX, YCDT, YIDG, YIDI, PGAC, AMPE, MIND, YJJL, YIJD, YBHS, YJIY, YJEM, RFAL, MTLA, ADIC, YBAN, DSBB, TONB, FUCP, YGJV, EXUT, ZRAS
SP_PIR_KEYWORDS	cell membrane	YHID, YGAZ, HTPG, MALF, YOHJ, MALG, HDED, PSTB, FABI, GLTK, GLTL, ATOS, MALK, YBBM, FECA, NIKD, NIKE, FECC, NIKB, NIKC, MIPA, YBBY, GNTP, MALX, FECE, FECD, SYD, YJET, YICE, WBBK, NUPG, YEDD, YCCM, NUPC, YBJM, BASS, YDCO, LNT, YAJI, YHHN, YDDG, NAPH, NAPC, OMPC, KDPA, DDPB, GLNQ, YRFF, GALP, LAMB, GLNP, MANY, BTUB, MANZ, MTGA, YIAD, LPD, CCMC, CCMD, CCMA, CCMB, CYNX, SAPB, NIRC, YAAJ, EXBD, TSX, EXBB, CCME, FHUC, YEAI, FRDC, DCUC, DCUB, NEPI, NARX, TQSA, NARH, NARG, NARI, MDTF, MDTL, NARK, FHUB, FHUA, YIGM, NLPE, FEOB, HEMX, YPDA, YIHY, YHCB, ARCB, YHBX, YCDT, YIDE, YQGA, YIDG, YIDI, YNBD, PGAA, PGAC, PGAB, AMPE, MIND, YJJL, YIJD, YBHS, FADL, YJIY, YJEM, RFAL, MTLA, ADIC, YBAN, DSBB, TONB, FUCP, YGJV, EXUT, YJDO, ZRAS, BAMC, BAMA

Annotation Cluster 4	Enrichment Score: 11.08469928693394	
Category	Term	Genes
KEGG_PATHWAY	eco00030:Pentose phosphate pathway	TALB, GND, FBP, ZWF, IDNK, GHRB, RPIB, DEOC, DEOB, RPIA, EDD, EDA, PGM, GLPX, PFKA, PGL

Annotation Cluster 5	Enrichment Score: 8.976102835290327	
Category	Term	Genes
KEGG_PATHWAY	ecg00010:Glycolysis / Gluconeogenesis	ENO, TPIA, MALX, PYKF, GPMM, CRR, PCK, LPD, FBP, AGP, GALM, GPMA, BGLA, PGM, GLPX, PFKA

Annotation Cluster 6	Enrichment Score: 8.855452864749044	
Category	Term	Genes
SP_PIR_KEYWORDS	periplasm	USHA, FHUD, CPDB, YBHG, YCFS, NAPA, MALE, NAPB, HDEA, MALM, AGP, SPY, CSGC, TPX, GLTI, FECB, MALS, SLT, NIKA, GLNH, NRFA, DSBA, YHJJ, FECR, YBCL, RNA, MPPA, PPIA, LOLA, DCRB, SODC
SP_PIR_KEYWORDS	signal	FHUD, CPDB, YIBG, MALG, YCFS, MALE, HDEB, HDEA, MALM, NRFC, SPY, CSGC, YMGD, GLTI, TQSA, FECA, FECB, FECC, MALS, SLT, NIKC, MIPA, FHUA, NIKA, NLPE, YACC, YIIQ, YCGJ, YBJH, YBCL, YCGK, YEDD, RNA, MPPA, LOLA, EUTM, LNT, YAJI, DCRB, USHA, NAPG, PGAA, YBHG, PGAB, NAPA, YADE, YNFD, NAPB, OMPC, AGP, LAMB, FADL, BTUB, GLNH, NRFA, YIAD, YHJJ, DSBA, CYNX, PPIA, YEEZ, BAMC, TSX, BAMA, SODC

GOTERM_CC_FAT	GO:0042597~periplasmic space	FHUD, USHA, CPDB, YBHG, NAPD, YCFS, NAPA, MALE, NAPB, HDEA, GALM, MALM, AGP, SPY, TPX, GLTI, FECB, MALS, SLT, NIKA, GLNH, NRFA, DSBA, YHJJ, FECR, TONB, CCMA, YBCL, RNA, MPPA, PPIA, LOLA, DCRB, SODC
UP_SEQ_FEATURE	signal peptide	FHUD, CPDB, YIBG, MALG, YCFS, MALE, HDEB, HDEA, MALM, NRFC, SPY, CSGC, YMGD, GLTI, TQSA, FECA, FECB, FECC, MALS, SLT, NIKC, MIPA, FHUA, NIKA, NLPE, YACC, YIIQ, YCGJ, YBJH, YBCL, YCGK, YEDD, RNA, MPPA, LOLA, EUTM, LNT, YAJI, DCRB, USHA, NAPG, PGAA, YBHG, PGAB, NAPA, YADE, YNFD, NAPB, OMPC, AGP, LAMB, FADL, BTUB, GLNH, NRFA, YIAD, YHJJ, DSBA, CYNX, PPIA, YEEZ, BAMC, TSX, BAMA, SODC
Annotation Cluster 7	Enrichment Score: 8.587064481568945	
Category	Term	Genes
KEGG_PATHWAY	ecx00051:Fructose and mannose metabolism	MANZ, TPIA, FUCK, MTLA, MAK, MANA, MTLD, FUCI, SRLD, FBP, SRLB, MANY, GLPX, PFKA
Annotation Cluster 8	Enrichment Score: 8.251536126069754	
Category	Term	Genes
SP_PIR_KEYWORDS	ATP	FHUC, PCK, MIND, SERS, KDPA, PSTB, GALK, GLTL, MALK, GLNQ, FHLA, GLNS, MFD, NIKD, MDTF, NIKE, GLNL, GLNG, SUFC, HSLU, UVRB, CCMA, HELD, MURF, CLPP, GROS, GSHB, PFKA, GROL
UP_SEQ_FEATURE	nucleotide phosphate-binding region:ATP	FHUC, PURT, MIAA, PCK, MIND, SERS, PSTB, GALK, GLPK, GLTL, YBBL, MALK, GLNQ, MFD, FHLA, RARA, NIKD, NIKE, DING, HEPA, GLNG, ANMK, SUFC, NADE, MAK, HSLU, FECE, UVRB, DDLA, IDNK, CCMA, HELD, SUCC, MURF, YBJD, YEEZ, GSHB, PRKB, EUTP, PFKA
SMART	SM00382:AAA	GLNG, FHUC, SUFC, DNAC, FECE, HSLU, YBHF, PSTB, GLTL, YBBL, CCMA, MALK, GLNQ, FHLA, RARA, NIKD, NIKE
INTERPRO	IPR003593:ATPase, AAA+ type, core	GLNG, FHUC, SUFC, DNAC, FECE, HSLU, YBHF, PSTB, GLTL, YBBL, CCMA, MALK, GLNQ, FHLA, RARA, NIKD, NIKE
GOTERM_MF_FAT	GO:0016887~ATPase activity	FHUC, SUFC, FECE, HSLU, YBHF, MIND, PSTB, KDPA, GLTL, YBBL, CCMA, HELD, MALK, GLNQ, MFD, NIKD, YBHS, NIKE, NIKB, NIKC, DING, NIKA
Annotation Cluster 9	Enrichment Score: 7.40947326383826	
Category	Term	Genes
KEGG_PATHWAY	ecd02010:ABC transporters	FHUC, FHUD, MALF, MALG, MALE, PSTB, GLTK, GLTL, DDPB, MALK, GLNQ, GLTI, GLNP, FECB, NIKD, FECC, NIKE, YBHS, NIKB, NIKC, FHUB, NIKA, GLNH, FECE, FECD, CCMC, CCMA, CCMB, MPPA, SAPB
Annotation Cluster 10	Enrichment Score: 7.169711766510374	
Category	Term	Genes

SP_PIR_KEYWORDS	nucleotide-binding	FHUC, CPDB, CRP, HTPG, PCK, NARX, PSTB, METL, GLPK, GLTL, YBBL, ATOS, GSP, MALK, MFD, NIKD, RARA, NIKE, NARL, YIHA, DING, EUTT, ANMK, ENTF, HSLU, FECE, UVRB, THIK, FEOB, PURC, PURA, SUCC, MURF, ARCB, YBJD, YAJQ, CLPP, GLYQ, GSHB, RIBA, PRKB, BASS, EUTP, PFKA, XYLB, PURT, MIAA, USHA, DNAC, YJIA, DHAL, CCA, YBHF, MIND, HSCC, SERS, KDPA, HYPB, GALK, MOAB, GLNQ, RFAY, FHLA, GLNS, ASNA, NRDE, RFAP, ASNC, GLNL, HEPA, GLNG, SUFC, FUCK, PYKF, NADE, MAK, GLNA, NADK, FBP, DDLA, IDNK, CCMA, HELD, MOBA, ZRAS, YEEZ, COAD, SELB, GROL
GOTERM_MF_FAT	GO:0000166~nucleotide binding	LDHA, MAEB, MAEA, HTPG, MHPF, PSTB, GLTL, ATOS, YBBL, FABG, GOR, MALK, MFD, NIKD, RARA, NIKE, PANE, PDXH, EUTT, ENTF, FECE, UVRB, THIK, SUCC, YBJD, YAJQ, CLPP, RIBA, PRKB, BASS, EUTP, UBIF, PURT, MIAA, DNAC, GND, YJIA, DHAL, SERS, YQCA, KDPA, GALK, MOAB, GLNQ, GRPE, GLNS, NRDE, GLNL, GLNG, HEPA, PYKF, GLNA, LPD, NIRB, CCMA, MOBA, GROS, YEEZ, COAD, GROL, FHUC, CPDB, CRP, PCK, NARX, METL, GLPK, GSP, YIGZ, HEMG, NARL, AZOR, YIHA, DING, ANMK, AHPF, HSLU, FEOB, PURC, HYFG, YPDA, PURA, GHRB, MURF, ARCB, GSHB, TRXB, GLYQ, PFKA, XYLB, USHA, CCA, YBHF, HSCC, MIND, HYPB, USPF, FHLA, ASNA, RFAP, YBHS, ASNC, SUFC, FUCK, NADE, MAK, NADK, FBP, DDLA, RFAD, IDNK, GPSA, HELD, ZRAS, NEMA, SELB
GOTERM_MF_FAT	GO:0032553~ribonucleotid e binding	FHUC, CRP, HTPG, PCK, NARX, METL, PSTB, GLPK, GLTL, YBBL, ATOS, GSP, YIGZ, MALK, MFD, NIKD, RARA, NIKE, NARL, YIHA, DING, EUTT, ANMK, ENTF, HSLU, FECE, UVRB, THIK, FEOB, PURC, YPDA, PURA, SUCC, MURF, ARCB, YBJD, CLPP, GLYQ, GSHB, RIBA, PRKB, BASS, EUTP, PFKA, XYLB, PURT, MIAA, DNAC, YJIA, DHAL, CCA, YBHF, MIND, HSCC, SERS, KDPA, HYPB, GALK, USPF, MOAB, GLNQ, FHLA, GLNS, ASNA, NRDE, RFAP, ASNC, YBHS, GLNL, HEPA, GLNG, SUFC, FUCK, PYKF, NADE, MAK, GLNA, NADK, DDLA, IDNK, CCMA, HELD, MOBA, GROS, ZRAS, YEEZ, COAD, SELB, GROL
Annotation Cluster 11	Enrichment Score: 7.157803847119591	
Category	Term	Genes
SP_PIR_KEYWORDS	phosphoprotein	ENO, CRR, HTPG, NARX, USPA, PTSI, ATOS, AGP, PTSH, PTSA, KBL, CPXR, HEML, NARL, PGM, UVRY, GLNL, MALP, CHBB, GLNG, ENTF, MALX, NADE, MTLA, GLNA, SRLB, GLYA, ACPP, YPDB, TORR, ARCB, GPMA, BAER, QSEB, ZRAS, GLMM, BASS, BASR, GROL
KEGG_PATHWAY	ecd02020:Two-component system	GLNG, MAEA, FRDC, DCUB, GLNA, NARX, OMPC, KDPA, TORR, ATOS, ARCB, BAER, NARH, QSEB, ZRAS, NARG, NARJ, CPXR, NARI, NARL, BASS, BASR, UVRY, GLNL
Annotation Cluster 12	Enrichment Score: 6.914277362344854	
Category	Term	Genes
GOTERM_BP_FAT	GO:0051186~cofactor metabolic process	FUMB, MOG, HEMB, HEMC, GOR, HEMD, HEMG, HEML, PANE, ENTF, FSAB, EUTT, MELA, MOEA, GLYA, HEMX, ENTB, SUCB, SUCC, GSHB, UBIE, UBIF, TPIA, NFUA, GND, NAPA, MIND, ZWF, MOAC, MOAB, PPC, RPIB, GALE, RPIA, SUFD, MENB, NADC, TALB, TESB, SUFB, NADE, CYSG, LPD, RFAD, BIOH, FOLP, YFBB, MOBA, COAD, FOLD

Annotation Cluster 13	Enrichment Score: 6.209774307271593	
Category	Term	Genes
SP_PIR_KEYWORDS	nitrate assimilation	NIRB, NIRD, NIRC, NARH, NARG, NARX, NAPA, NARJ, NARI, NARL, NARK
GOTERM_MF_FAT	GO:0016661~oxidoreductase activity, acting on other nitrogenous compounds as donors	NRFA, HCP, NAPG, NAPH, NAPC, NAPD, NAPA, NAPB, QUEF, NIRB, NRFC, NIRD, NARH, NARG, NARJ, NARI
GOTERM_BP_FAT	GO:0042128~nitrate assimilation	NIRB, NIRD, NIRC, NARH, NARG, NARX, NAPA, NARJ, NARI, NARL, NARK
KEGG_PATHWAY	ecj00910:Nitrogen metabolism	NIRB, NRFA, NADE, MALY, GLNA, NIRD, NARH, NARG, ASNA, NAPA, NARJ, NARI

Annotation Cluster 14	Enrichment Score: 6.0238501611374735	
Category	Term	Genes
KEGG_PATHWAY	eum00230:Purine metabolism	PURH, PURT, USHA, PURF, PYKF, CPDB, PURN, HPT, POLA, PURC, PURB, PURA, NRDF, DEOD, NRDE, DEOB, ADE, ADD

Annotation Cluster 15	Enrichment Score: 5.398310024763359	
Category	Term	Genes
GOTERM_BP_FAT	GO:0009410~response to xenobiotic stimulus	AHPC, NLPE, CYNX, TPX, AHPF, EUTM, CPXR, EUTN, NEMA, SODA, DTD, SODC
GOTERM_BP_FAT	GO:0006805~xenobiotic metabolic process	AHPC, NLPE, CYNX, TPX, AHPF, EUTM, CPXR, EUTN, NEMA, SODA, DTD, SODC
GOTERM_MF_FAT	GO:0016209~antioxidant activity	AHPC, GOR, TPX, AHPF, TRXB, SODA, SODC

Annotation Cluster 16	Enrichment Score: 5.239692204335017	
Category	Term	Genes
SP_PIR_KEYWORDS	magnesium	PURT, MIAA, ENO, UPP, CCA, XTHA, PTSI, HEMB, PTSA, RNHA, DXS, RFAP, PPA, YIDA, PGM, PURF, PYKF, MOEA, HPT, FBP, DDLA, KDSB, PURA, SUCC, MOBA, RIBA, GLMM, GSHB, YBJI, PFKA

Annotation Cluster 17	Enrichment Score: 4.999456300041487	
Category	Term	Genes
GOTERM_BP_FAT	GO:0019318~hexose metabolic process	YJJW, ENO, TPIA, MAEA, GPMM, GND, PCK, ZWF, GALK, GLGA, GALM, AGP, RHAR, RPIB, GALE, RPIA, FUCU, GALF, PGM, PGL, FSAB, FSAA, TALB, PYKF, FUCK, FUCI, GALT, LPD, FBP, FUCP, YBIY, GPMA, GLGX, GLMM, PRKB, PFKA

GOTERM_BP_FAT	GO:0005996~monosaccharide metabolic process	YJJW, ENO, MAEA, TPIA, GPMM, GND, UXUA, PCK, ZWF, GALK, GLGA, AGP, GALM, RHAR, UXAC, GALE, RPIB, RPIA, FUCU, PGM, GALF, PGL, ANMK, FSAB, FSAA, AGAA, TALB, FUCK, KDSA, PYKF, FUCI, GALT, LPD, FBP, KDSB, FUCP, YBIY, GPMA, GLGX, AGAW, GLMM, PRKB, PFKA, AGAV, XYLB
SP_PIR_KEYWORDS	glycolysis	ENO, TPIA, PYKF, GPMM, GPMA, LPD, PFKA
GOTERM_BP_FAT	GO:0006739~NADP metabolic process	FSAB, TPIA, TALB, GND, RPIB, RPIA, ZWF
Annotation Cluster 18	Enrichment Score: 4.923901306570979	
Category	Term	Genes
SP_PIR_KEYWORDS	iron transport	FHUC, ENTF, FHUD, FECI, FECE, FECD, FECR, FEOB, FEOA, TONB, ENTB, FECA, FECB, FECC, FHUB, FHUA
SP_PIR_KEYWORDS	nickel	HYCI, CCA, NIKD, GLOA, NIKE, NIKB, NIKC, SLYD, HYPA, NIKR, NIKA, HYPB
SP_PIR_KEYWORDS	ion transport	FHUC, ENTF, FHUD, FECI, FECE, FECD, FECR, FEOB, FEOA, ENTB, OMPC, KDPA, LAMB, FECA, FECB, FECC, TSX, FHUB, BTUB, FHUA
GOTERM_BP_FAT	GO:0006826~iron ion transport	FHUC, ENTF, FHUD, FECI, FECE, FECD, FECR, FEOB, FTNB, FEOA, TONB, ENTB, FECA, FECB, FECC, EXBD, EXBB, FHUB, FHUA
GOTERM_BP_FAT	GO:0000041~transition metal ion transport	FHUC, ENTF, FHUD, FECI, FECE, FECD, FECR, FEOB, FTNB, FEOA, TONB, ENTB, FECA, FECB, NIKD, FECC, NIKE, EXBD, NIKB, EXBB, NIKC, FHUB, FHUA
GOTERM_BP_FAT	GO:0006811~ion transport	FHUC, FHUD, YIDE, YFEH, OMPC, PSTB, KDPA, UIDB, LAMB, FECA, FECB, NIKD, FECC, NIKE, NIKB, PHOU, NIKC, NARK, FHUB, BTUB, FHUA, ENTF, FECI, FECE, FECD, SSEA, FECR, FEOB, FEOA, FTNB, TONB, ENTB, YAAJ, EXBD, TSX, EXBB
Annotation Cluster 19	Enrichment Score: 4.740461291101534	
Category	Term	Genes
SP_PIR_KEYWORDS	phosphotransferase	CHBB, MANZ, MALX, PYKF, MTLA, CRR, NARX, SRLB, GALK, GLPK, PTSI, ATOS, ARCB, PRKB, MANY, PFKA, GLNL, XYLB
SP_PIR_KEYWORDS	sugar transport system	MANZ, PTSI, PTSH, MALX, CRR, MTLA, SRLB, MANY
KEGG_PATHWAY	ecd02060:Phosphotransferase system (PTS)	CHBB, MANZ, MALX, MTLA, CRR, SRLB, YADI, PTSI, PTSH, PTSA, ULAC, MANY, AGAV
SP_PIR_KEYWORDS	sugar transport	CHBB, MANZ, GNTP, MALX, MTLA, CRR, MALF, MALG, SRLB, MALE, YADI, FUCP, PTSI, MALM, PTSH, MALK, GALP, LAMB, PTSA, MANY, AGAV
GOTERM_BP_FAT	GO:0008643~carbohydrate transport	CRR, MALF, MALG, MALE, YADI, PTSI, MALM, PTSH, MALK, GALP, PTSA, LAMB, MANY, FUCU, CHBB, MANZ, GNTP, MALX, MTLA, SRLB, FUCP, GUTM, AGAW, ULAC, AGAV
Annotation Cluster 20	Enrichment Score: 4.653863928820876	
Category	Term	Genes

KEGG_PATHWAY	ecx00520:Amino sugar and nucleotide sugar metabolism	MANZ, GALK, MAK, MANA, CRR, GALT, GALE, GLMM, MANY, GALF, PGM, NAGZ
GOTERM_BP_FAT	GO:0009242~colanic acid biosynthetic process	GALK, MANA, GALT, GALE

Annotation Cluster 21	Enrichment Score: 4.615717451270133	
Category	Term	Genes
GOTERM_BP_FAT	GO:0015949~nucleobase, nucleoside and nucleotide interconversion	PURH, UDP, UPP, CPDB, HPT, PURA, NUPG, NUPC, CYTR, NRDF, DEOD, NRDE, DEOC, CDD, MTN, DEOB, TSX, DEOA, RNK, ADD
KEGG_PATHWAY	ecv00240:Pyrimidine metabolism	USHA, UDP, UPP, CPDB, DEOD, NRDF, NRDE, TRXB, CDD, POLA, DEOA

Annotation Cluster 22	Enrichment Score: 4.554719739185943	
Category	Term	Genes
GOTERM_MF_FAT	GO:0042280~cell surface antigen activity, host-interacting	RFAF, KDSA, RFAL, RFAI, RFAJ, GALT, KDSB, RFAD, WAAU, RFAB, GALK, RFAZ, LPCA, RFAY, GLMM, RFAP, GALE, COAD, RFAS
KEGG_PATHWAY	ecj00540:Lipopolysaccharide biosynthesis	RFAF, KDSA, RFAL, RFAI, RFAJ, KDSB, RFAD, WAAU, RFAB, LPCA, RFAY, GMHB, RFAP
GOTERM_BP_FAT	GO:0016051~carbohydrate biosynthetic process	ENO, TPIA, MAEA, MANA, PCK, WAAU, GALK, LDCA, GLGA, RFAZ, RFAY, RFAP, GALE, GALF, SLT, RFAS, MIPA, MTGA, RFAF, KDSA, RFAL, RFAI, RFAJ, GALT, FBP, DDLA, KDSB, RFAD, WBBK, RFAB, GPSA, MURF, LPCA, GPMA, GMHB, GLGX, MURI, AGAW, GLMM, AGAV, NAGZ

Annotation Cluster 23	Enrichment Score: 4.287359536606784	
Category	Term	Genes
KEGG_PATHWAY	ecf00040:Pentose and glucuronate interconversions	UXAA, UXAB, UXUA, UXAC, UXUB, EDA, GALF, XYLB

Annotation Cluster 24	Enrichment Score: 4.0638673942719885	
Category	Term	Genes
KEGG_PATHWAY	ecx00500:Starch and sucrose metabolism	GLGA, MALX, MAK, CRR, MALS, GALF, PGM, MALQ, MALP

Annotation Cluster 25	Enrichment Score: 4.021769067741989	
Category	Term	Genes
SP_PIR_KEYWORDS	heat shock	HSLU, CLPP, HTPG, HSLV, GROS, GROL
SP_PIR_KEYWORDS	stress-induced protein	HSLU, HTPG, HSLV, GROS, GROL

Annotation Cluster 26	Enrichment Score: 3.960141562655923	
Category	Term	Genes
SP_PIR_KEYWORDS	glycolysis	ENO, TPIA, PYKF, GPMM, GPMA, LPD, PFKA
INTERPRO	IPR005952:Phosphoglycerate mutase 1	GPMA
GOTERM_MF_FAT	GO:0016866~intramolecular transferase activity	LPCA, GPMM, GPMA, RLUA, GLMM, DEOB, HEML, PGM

Annotation Cluster 27	Enrichment Score: 3.9049103811687464	
Category	Term	Genes
GOTERM_BP_FAT	GO:0009110~vitamin biosynthetic process	PDXH, MENB, EUTT, NADC, NADE, CYSG, MELA, DKGB, DKGA, GLYA, MIND, RFAD, SELA, FOLP, BIOH, YFBB, RIBC, DXS, RIBA, GALE, UBIE, FOLD

Annotation Cluster 28	Enrichment Score: 3.5432343804934217	
Category	Term	Genes
SP_PIR_KEYWORDS	carbon-oxygen lyase	UXAA, CAIB, HEMB, HEMD, UXUA, MGSA, UXUB, FUMB
SP_PIR_KEYWORDS	hydro-lyase	UXAA, CAIB, HEMB, HEMD, UXUA, UXUB, FUMB
GOTERM_MF_FAT	GO:0016836~hydro-lyase activity	UXAA, ENO, HEMB, MOAB, HEMD, UXUA, EDD, FUMB

Annotation Cluster 29	Enrichment Score: 3.5189824617412615	
Category	Term	Genes
SP_PIR_KEYWORDS	iron	FHUC, YJJW, FHUD, NAPG, NAPH, NFUA, NAPF, NAPC, NAPA, NAPB, FUMB, UXAA, NRFC, NARH, FECA, NARG, NRDF, YJES, FECB, FECC, NARI, FHUB, FHUA, HCR, ENTF, GLCF, FECI, NRFA, HCP, FECE, FECD, GALT, FEOB, FECR, HMP, FEOA, ENTB, HYFG, YSAA, NIRB, RUMA, YCCM, YBIY, YGCO, CCME
SP_PIR_KEYWORDS	electron transport	GLCF, HCR, NRFA, NAPG, NAPH, NAPF, NAPC, DSBB, NAPA, NAPB, YSAA, YQCA, GRXB, GRXC, NRFC, YCCM, GRXD, NARH, NARG, NARI, YGCO
GOTERM_MF_FAT	GO:0016661~oxidoreductase activity, acting on other nitrogenous compounds as donors	NRFA, HCP, NAPG, NAPH, NAPC, NAPD, NAPA, NAPB, QUEF, NIRB, NRFC, NIRD, NARH, NARG, NARJ, NARI

SP_PIR_KEYWORDS	4fe-4s	YJJW, GLCF, NAPG, NAPH, NFUA, NAPF, NAPA, YSAA, FUMB, HYFG, RUMA, NIRB, NRFC, YCCM, YBIY, NARH, NARG, YJES
GOTERM_MF_FAT	GO:0005506~iron ion binding	FHUC, YJJW, FHUD, NAPG, NAPH, NFUA, NAPF, NAPC, NAPA, NAPB, FUMB, YQCA, UXAA, NRFC, NARH, FECA, NARG, NRDF, YJES, FECB, FECC, NARI, FHUB, FHUA, HCR, ENTF, GLCF, FECI, NRFA, HCP, FECE, FECD, GALT, FEOB, FECR, HMP, FTNB, FEOA, ENTB, YSAA, HYFG, NIRB, RUMA, YCCM, YBIY, YGCO, CCME
GOTERM_MF_FAT	GO:0008940~nitrate reductase activity	NAPG, NAPH, NAPC, NARH, NAPD, NARG, NAPA, NARJ, NAPB, NARI
Annotation Cluster 30	Enrichment Score: 3.3648891683359463	
Category	Term	Genes
SP_PIR_KEYWORDS	porphyrin biosynthesis	HEMB, HEMC, HEMD, CYSG, HEMG, HEMX, HEML
KEGG_PATHWAY	ecj00860:Porphyrin and chlorophyll metabolism	EUTT, HEMB, HEMC, HEMD, CYSG, HEMG, HEMX, HEML
Annotation Cluster 31	Enrichment Score: 3.2263528130035257	
Category	Term	Genes
KEGG_PATHWAY	ecx00480:Glutathione metabolism	GSP, PEPD, GOR, GND, GSHB, ZWF
Annotation Cluster 32	Enrichment Score: 3.2086828653133614	
Category	Term	Genes
GOTERM_BP_FAT	GO:0017004~cytochrome complex assembly	CUTA, CCMC, CCMD, CCMA, CCMB, NRFA, NAPC, NAPB, NARI, CCME
SP_PIR_KEYWORDS	chromoprotein	NRFA, NAPC, HMP, NARI, CCME
GOTERM_BP_FAT	GO:0043933~macromolecular complex subunit organization	CUTA, FECI, NRFA, NAPC, NAPB, YAEJ, HYPB, PRFA, CCMC, CCMD, CCMA, SECB, CCMB, NARI, CCME
Annotation Cluster 33	Enrichment Score: 3.1804594750438735	
Category	Term	Genes
SP_PIR_KEYWORDS	phage recognition	LAMB, TSX, FADL, TONB, OMPC, BTUB, FHUA
GOTERM_BP_FAT	GO:0051606~detection of stimulus	GLNG, LAMB, TSX, FADL, TONB, OMPC, BTUB, FHUA
GOTERM_BP_FAT	GO:0009581~detection of external stimulus	LAMB, TSX, FADL, TONB, OMPC, BTUB, FHUA
SP_PIR_KEYWORDS	cell outer membrane	NLPE, PGAA, LAMB, FECA, BAMC, TSX, FADL, BAMA, OMPC, BTUB, FHUA, MIPA
SP_PIR_KEYWORDS	receptor	LAMB, FECA, TSX, TONB, BTUB, YHHM, FHUA

SP_PIR_KEYWORDS	Porin	LAMB, TSX, OMPC, BTUB
GOTERM_CC_FAT	GO:0046930~pore complex	LAMB, TSX, OMPC, BTUB
GOTERM_CC_FAT	GO:0009279~cell outer membrane	NLPE, PGAA, YBHG, YIAD, OMPC, LAMB, FECA, BAMC, MDTF, TSX, FADL, BAMA, BTUB, MIPA, FHUA
GOTERM_CC_FAT	GO:0044462~external encapsulating structure part	GLNH, NLPE, PGAA, YBHG, DSBA, YIAD, TONB, OMPC, CCMA, GLTI, LAMB, FECA, LOLA, BAMC, MDTF, FADL, TSX, BAMA, BTUB, FHUA, MIPA
GOTERM_CC_FAT	GO:0030313~cell envelope	PGAA, YBHG, OMPC, GLTK, YRFF, GLTI, LAMB, FECA, GLNP, MDTF, FADL, MIPA, FHUA, BTUB, GLNH, NLPE, DSBA, YIAD, FEOB, TONB, CCMA, LOLA, BAMC, TSX, BAMA

Annotation Cluster 34	Enrichment Score: 2.9848364695122975	
Category	Term	Genes
SP_PIR_KEYWORDS	disulfide bond	AHPC, USHA, AHPF, DSBA, LPD, DSBB, HDEA, GRXB, YBCL, GRXC, GOR, RNA, LAMB, NRDE, TRXB, MALS, HSLO, SLT, FHUA, SODC
SP_PIR_KEYWORDS	Redox-active center	GRXB, AHPC, GRXC, GRXD, GOR, AHPF, DSBA, DSBB, LPD, TRXB, HSLO
UP_SEQ_FEATURE	disulfide bond	AHPC, USHA, AHPF, DSBA, LPD, DSBB, HDEA, GRXB, YBCL, GRXC, GOR, RNA, LAMB, NRDE, TRXB, MALS, HSLO, SLT, FHUA, SODC
GOTERM_BP_FAT	GO:0045454~cell redox homeostasis	GRXB, AHPC, GRXC, GRXD, GOR, TPX, AHPF, YBBN, DSBA, LPD
GOTERM_MF_FAT	GO:0016667~oxidoreductase activity, acting on sulfur group of donors	GRXB, GRXC, GRXD, GOR, AHPF, DSBA, DSBB, LPD, TRXB
GOTERM_BP_FAT	GO:0019725~cellular homeostasis	GRXB, AHPC, GRXC, GRXD, GOR, TPX, AHPF, YBBN, DSBA, LPD, FTNB

Annotation Cluster 35	Enrichment Score: 2.9062381357974165	
Category	Term	Genes
GOTERM_BP_FAT	GO:0046165~alcohol biosynthetic process	GPSA, ENO, MAEA, TPIA, KDSA, GPMA, PCK, AGAW, FBP, KDSB, AGAV

Annotation Cluster 36	Enrichment Score: 2.9034072021863233	
Category	Term	Genes
INTERPRO	IPR005670:Phosphate transport system permease protein 1	PSTB
INTERPRO	IPR015850:ABC transporter, phosphate import, PstB	PSTB

GOTERM_MF_FAT	GO:0042625~ATPase activity, coupled to transmembrane movement of ions	NIKD, NIKE, NIKB, MIND, NIKC, KDPA, NIKA, PSTB
GOTERM_MF_FAT	GO:0015103~inorganic anion transmembrane transporter activity	MIND, NARK, PSTB
GOTERM_BP_FAT	GO:0015698~inorganic anion transport	SSEA, PHOU, NARK, PSTB
PIR_SUPERFAMILY	PIRSF002759:inner membrane protein MalK	FHUC, MALK, PSTB

Annotation Cluster 37	Enrichment Score: 2.8300261754331455	
Category	Term	Genes
KEGG_PATHWAY	ecj00620:Pyruvate metabolism	MAEB, LDHA, MAEA, PYKF, MGSA, PCK, PPC, LPD, GLOA, MHPF

Annotation Cluster 38	Enrichment Score: 2.824104862323159	
Category	Term	Genes
UP_SEQ_FEATURE	nucleotide phosphate-binding region:NADP	MAEB, YDBC, DKGB, DKGA, HMP, RFAD, METL, YHDH, FABG, YAJO, YQHD, YDHF, PANE, YDFG
PIR_SUPERFAMILY	PIRSF000097:aldo-keto reductase	YAJO, YDBC, DKGB, DKGA, YDHF
INTERPRO	IPR001395:Aldo/keto reductase	YAJO, YDBC, DKGB, DKGA, YDHF

Annotation Cluster 39	Enrichment Score: 2.7814636880035564	
Category	Term	Genes
SP_PIR_KEYWORDS	purine nucleotide biosynthesis	PURH, PURT, PURN, PURC, PURB, PURA
SP_PIR_KEYWORDS	purine biosynthesis	PURH, PURT, PURF, PURN, PURC, PURB, FOLD, PURA
KEGG_PATHWAY	ecd00670:One carbon pool by folate	PURH, PURT, PURN, GLYA, FOLD
GOTERM_BP_FAT	GO:0006164~purine nucleotide biosynthetic process	PURH, PURT, PURF, PURN, PURC, PURB, FOLD, PURA, ADD
GOTERM_BP_FAT	GO:0006163~purine nucleotide metabolic process	PURH, PURT, PURF, PURN, PURC, PURB, FOLD, PURA, ADD

Annotation Cluster 40	Enrichment Score: 2.540938451274983	
Category	Term	Genes
SP_PIR_KEYWORDS	glycerol metabolism	GLPK, DHAL, DHAK, GLDA, GLPD
KEGG_PATHWAY	ecd00561:Glycerolipid metabolism	GLPK, MELA, DHAL, DHAK, GLDA
GOTERM_BP_FAT	GO:0019400~alditol metabolic process	GLPK, GPSA, DHAL, DHAK, GLDA, GLPD, GLPX
GOTERM_BP_FAT	GO:0019751~polyol metabolic process	GLPK, GPSA, DHAL, DHAK, GLDA, GLPD, GLPX
Annotation Cluster 41	Enrichment Score: 2.377372725886059	
Category	Term	Genes
SP_PIR_KEYWORDS	DNA binding	GLNG, SFSA, HUPA, ADIY, CRP, POLA, HHA, MPRA, HNS, CSPC, TORR, HELD, RHAR, MALI, FHLA, MELR, CYTR, GUTM, ASNC, NARL, TDCA, YAHB
SP_PIR_KEYWORDS	activator	GLNG, HEPA, GADE, NHAR, CRP, CSPC, TORR, CHBR, RHAR, FHLA, MELR, QSEB, GUTM, ASNC, NARL, BASR, TDCA
SP_PIR_KEYWORDS	dna-binding	SFSA, ADIY, HUPA, CRP, YIHW, HHA, CSPC, MALI, MFD, FABR, ZUR, NANR, NARL, UVRY, TDCA, DING, NHAR, FECI, HSLR, POLA, HNS, YPDB, NIKR, CHBR, ARGP, MELR, GUTM, BASR, YDCN, GADE, YIDF, YJJM, RHAR, MTLR, FHLA, CYTR, CPXR, ASNC, HFQ, GREB, SEQA, GLNG, HEPA, YBIH, UIDR, MPRA, TORR, HELD, YIDZ, BAER, DKSA, QSEB, GCVR, YAHB
SP_PIR_KEYWORDS	transcription regulation	GADE, ADIY, CRP, YIHW, YJJM, CSPC, RHAR, MALI, MTLR, FHLA, CYTR, FABR, CPXR, ASNC, ZUR, PHOU, NANR, NARL, UVRY, TDCA, GREB, HEPA, GLNG, NHAR, FECI, YBIH, MALY, UIDR, MPRA, HNS, YPDB, NIKR, TORR, CHBR, ARCB, YIDZ, ARGP, BAER, MELR, QSEB, GUTM, GCVR, YDCN, BASR, YAHB
SP_PIR_KEYWORDS	Transcription	GADE, ADIY, CRP, YIHW, YJJM, CSPC, RHAR, MALI, MTLR, MFD, FHLA, CYTR, FABR, CPXR, ASNC, ZUR, NANR, NARL, TDCA, UVRY, GREB, HEPA, GLNG, NHAR, FECI, YBIH, MALY, UIDR, MPRA, HNS, YPDB, NIKR, TORR, CHBR, ARCB, YIDZ, ARGP, BAER, MELR, QSEB, GUTM, GCVR, YDCN, BASR, YAHB
GOTERM_BP_FAT	GO:0045449~regulation of transcription	ADIY, CRP, YIHW, CSPC, ATOS, UMUD, MALI, MFD, FABR, ZUR, NANR, NARL, UVRY, TDCA, NHAR, FECI, MALY, HNS, YPDB, NIKR, CHBR, ARCB, ARGP, MELR, GUTM, BASR, YDCN, RNK, GADE, YJJM, RHAR, MTLR, FHLA, CYTR, CPXR, ASNC, HFQ, GLNL, GREB, GLNG, HEPA, YBIH, UIDR, MPRA, TORR, MOBA, YIDZ, BAER, QSEB, GCVR, YAHB
GOTERM_MF_FAT	GO:0003677~DNA binding	SFSA, ADIY, HUPA, CRP, XTHA, YIHW, HHA, CSPC, UMUD, MALI, MFD, FABR, RECR, ZUR, NANR, NARL, UVRY, TDCA, DING, NHAR, FECI, HSLR, UVRC, UVRB, POLA, HNS, YPDB, NIKR, CHBR, YBCK, ARGP, MELR, GUTM, MRR, YDCN, BASR, RNK, GADE, YIDF, YJJM, RHAR, MTLR, FHLA, CYTR, CPXR, ASNC, HFQ, GREB, SEQA, HEPA, GLNG, YBIH, UIDR, MPRA, TORR, HELD, YFBB, MOBA, YIDZ, BAER, DKSA, QSEB, GCVR, YAHB

Annotation Cluster 42	Enrichment Score: 2.345363719779025	
Category	Term	Genes
GOTERM_BP_FAT	GO:0046496~nicotinamide nucleotide metabolic process	FSAB, NADC, TPIA, TALB, NADE, MELA, GND, GALE, RPIB, RPIA, RFAD, ZWF
GOTERM_BP_FAT	GO:0006733~oxidoreduction coenzyme metabolic process	FSAB, NADC, TPIA, TALB, NADE, MELA, GND, RFAD, ZWF, RPIB, GALE, RPIA, UBIE, UBIF
GOTERM_BP_FAT	GO:0006739~NADP metabolic process	FSAB, TPIA, TALB, GND, RPIB, RPIA, ZWF

Annotation Cluster 43	Enrichment Score: 2.325988930791967	
Category	Term	Genes
SP_PIR_KEYWORDS	gluconate utilization	GNTP, GHRB, GND, IDNK

Annotation Cluster 44	Enrichment Score: 2.2198916805541873	
Category	Term	Genes
SP_PIR_KEYWORDS	GTP binding	HSLU, FEOB, SELB, YIHA, PURA
SP_PIR_KEYWORDS	gtp-binding	MOBA, MOAB, YJIA, FEOB, RIBA, SELB, YIHA, PURA, HYPB
GOTERM_MF_FAT	GO:0019001~guanyl nucleotide binding	MOBA, YIGZ, MOAB, YJIA, FEOB, RIBA, SELB, YIHA, PURA, HYPB

Annotation Cluster 45	Enrichment Score: 2.1960705749954004	
Category	Term	Genes
SP_PIR_KEYWORDS	Molybdenum cofactor biosynthesis	MOAC, MOBA, MOAB, MOEA, MOG
GOTERM_BP_FAT	GO:0042559~pteridine and derivative biosynthetic process	FOLP, MOAC, MOBA, MOAB, MOEA, NAPA, GLYA, MOG, FOLD
GOTERM_BP_FAT	GO:0019438~aromatic compound biosynthetic process	FOLP, MOAC, MOBA, MOAB, MOEA, DXS, NAPA, GLYA, MOG, FOLD

Annotation Cluster 46	Enrichment Score: 2.0143435177013824	
Category	Term	Genes
SP_PIR_KEYWORDS	intramolecular oxidoreductase	TPIA, MANA, UXAC, RPIB, RPIA

GOTERM_MF_FAT	GO:0016861~intramolecular oxidoreductase activity, interconverting aldoses and ketoses	TPIA, MANA, FUCI, UXAC, RPIB, RPIA
GOTERM_MF_FAT	GO:0016860~intramolecular oxidoreductase activity	TPIA, MANA, FUCI, UXAC, DSBA, RPIB, RPIA

Annotation Cluster 47	Enrichment Score: 1.9653252122747422	
Category	Term	Genes
SP_PIR_KEYWORDS	dna repair	YICR, HELD, UVRC, UMUD, MFD, UVRB, XTHA, RECR, POLA, DING
KEGG_PATHWAY	ecd03420:Nucleotide excision repair	UVRC, MFD, UVRB, POLA
SP_PIR_KEYWORDS	DNA damage	UVRC, UMUD, MFD, UVRB, XTHA, RECR, POLA, DING
SP_PIR_KEYWORDS	sos response	UVRC, UMUD, UVRB
GOTERM_BP_FAT	GO:0009432~SOS response	UVRC, UMUD, UVRB, DING
GOTERM_BP_FAT	GO:0033554~cellular response to stress	YICR, UVRC, UVRB, XTHA, POLA, XSEB, HELD, UMUD, MFD, RECR, TRXB, DING, YJIY
GOTERM_BP_FAT	GO:0006974~response to DNA damage stimulus	YICR, HELD, UVRC, UMUD, MFD, UVRB, XTHA, RECR, POLA, XSEB, DING
GOTERM_BP_FAT	GO:0006281~DNA repair	YICR, HELD, UVRC, UMUD, MFD, UVRB, XTHA, RECR, POLA, XSEB, DING
GOTERM_BP_FAT	GO:0006259~DNA metabolic process	YICR, UVRC, DNAC, UVRB, XTHA, POLA, XSEB, YBCK, HELD, YFBB, ARGP, UMUD, MFD, NRDF, RNHA, NRDE, RECR, MRR, DING, RDGC, SEQA

Annotation Cluster 48	Enrichment Score: 1.8326642273483613	
Category	Term	Genes
GOTERM_BP_FAT	GO:0034655~nucleobase, nucleoside, nucleotide and nucleic acid catabolic process	USHA, UDP, CPDB, DEOC, MTN, DEOB, ADE

Annotation Cluster 49	Enrichment Score: 1.8187390260480976	
Category	Term	Genes
SP_PIR_KEYWORDS	cell wall biogenesis/degradation	LDCA, MTGA, MURF, MURI, DDLA, AMPD, SLT, NAGZ
SP_PIR_KEYWORDS	peptidoglycan synthesis	LDCA, MTGA, MURF, MURI, DDLA, NAGZ
GOTERM_BP_FAT	GO:0006024~glycosaminoglycan biosynthetic process	LDCA, MTGA, MURF, MURI, GLMM, DDLA, SLT, NAGZ, MIPA

GOTERM_BP_FAT	GO:0006023~aminoglycan biosynthetic process	LDCA, MTGA, MURF, MURI, GLMM, DDLA, SLT, NAGZ, MIPA
GOTERM_BP_FAT	GO:0009252~peptidoglycan biosynthetic process	LDCA, MTGA, MURF, MURI, GLMM, DDLA, SLT, NAGZ, MIPA
SP_PIR_KEYWORDS	cell shape	LDCA, MTGA, MURF, MURI, DDLA, NAGZ
GOTERM_BP_FAT	GO:0030203~glycosaminoglycan metabolic process	LDCA, ANMK, MTGA, MURF, MPPA, MURI, GLMM, DDLA, AMPD, SLT, NAGZ, MIPA
GOTERM_BP_FAT	GO:0044036~cell wall macromolecule metabolic process	LDCA, MTGA, MURF, MURI, YCFS, GLMM, DDLA, SLT, NAGZ, MIPA
Annotation Cluster 50	Enrichment Score: 1.8098182404566971	
Category	Term	Genes
SP_PIR_KEYWORDS	cytochrome c-type biogenesis	CCMC, CCMD, CCMA, CCMB, CCME
GOTERM_MF_FAT	GO:0015232~heme transporter activity	CCMC, CCMA, CCMB
Annotation Cluster 51	Enrichment Score: 1.7688768560218913	
Category	Term	Genes
KEGG_PATHWAY	ecq00250:Alanine, aspartate and glutamate metabolism	PURF, GLNA, ASNA, PURB, PURA
Annotation Cluster 52	Enrichment Score: 1.6892681225175121	
Category	Term	Genes
GOTERM_MF_FAT	GO:0042625~ATPase activity, coupled to transmembrane movement of ions	NIKD, NIKE, NIKB, MIND, NIKC, KDPA, NIKA, PSTB
GOTERM_CC_FAT	GO:0019898~extrinsic to membrane	FHUC, PGAA, FECE, HTPG, LPD, MIND, WBBK, WAAU, PSTB, FABI, GLTL, CCMA, MALK, GLNQ, NARH, NARG, NIKD, NIKE
INTERPRO	IPR017871:ABC transporter, conserved site	FHUC, GLTL, YBBL, CCMA, SUFC, MALK, GLNQ, FECE, YBHF, NIKD, NIKE, PSTB
GOTERM_MF_FAT	GO:0016887~ATPase activity	FHUC, SUFC, FECE, HSLU, YBHF, MIND, PSTB, KDPA, GLTL, YBBL, CCMA, HELD, MALK, GLNQ, MFD, NIKD, YBHS, NIKE, NIKB, NIKC, DING, NIKA
Annotation Cluster 53	Enrichment Score: 1.6646227403377125	

Category	Term	Genes
KEGG_PATHWAY	ecw00300:Lysine biosynthesis	DAPB, MURF, DAPD, METL
SP_PIR_KEYWORDS	amino-acid biosynthesis	DAPB, MALY, ASNA, MTN, FOLD, DAPD, METL
Annotation Cluster 54	Enrichment Score: 1.5812159588603019	
Category	Term	Genes
KEGG_PATHWAY	ecd00020:Citrate cycle (TCA cycle)	SUCB, SUCC, FRDC, PCK, LPD, FUMB
GOTERM_BP_FAT	GO:0009060~aerobic respiration	SUCB, SUCC, ARCB, PPC, LPD, GLPD, NIKD, NIKE, UBIE, FUMB
GOTERM_BP_FAT	GO:0046356~acetyl-CoA catabolic process	SUCB, SUCC, PPC, LPD, FUMB
Annotation Cluster 55	Enrichment Score: 1.5361381339977807	
Category	Term	Genes
SP_PIR_KEYWORDS	cell cycle	MURF, GROS, YIIU, MIND, MINE, YIHA, NAGZ, GROL, MINC
GOTERM_BP_FAT	GO:0007049~cell cycle	MURF, GROS, YIIU, MIND, MINE, YIHA, GIDB, NAGZ, GROL, MINC
SP_PIR_KEYWORDS	septation	YIIU, MIND, YIHA, MINC
GOTERM_BP_FAT	GO:0051301~cell division	MURF, GROS, YIIU, MIND, MINE, YIHA, NAGZ, GROL, MINC
GOTERM_BP_FAT	GO:0000910~cytokinesis	YIIU, MIND, YIHA, MINC
Annotation Cluster 56	Enrichment Score: 1.4775373890213004	
Category	Term	Genes
GOTERM_BP_FAT	GO:0006542~glutamine biosynthetic process	GLNG, GLNH, GLNQ, GLNA, GLNP, GLNL
GOTERM_BP_FAT	GO:0009084~glutamine family amino acid biosynthetic process	GLTK, GLNG, GLNH, GLTL, GLNQ, GLNA, GLTI, GLNP, GLNL
KEGG_PATHWAY	ecy02020:Two-component system	GLNG, GLNA, GLNL
Annotation Cluster 57	Enrichment Score: 1.4706269517216743	
Category	Term	Genes
INTERPRO	IPR013816:ATP-grasp fold, subdomain 2	PURT, SUCC, GSHB, DDLA, PURC
INTERPRO	IPR011761:ATP-grasp fold	PURT, SUCC, GSHB, DDLA

Annotation Cluster 58	Enrichment Score: 1.4155748251734566	
Category	Term	Genes
KEGG_PATHWAY	ecs00010:Glycolysis / Gluconeogenesis	GALM, ENO, TPIA, GPMA, GLPX, PFKA

Annotation Cluster 59	Enrichment Score: 1.4107596819264208	
Category	Term	Genes
KEGG_PATHWAY	ecc00970:Aminoacyl-tRNA biosynthesis	SELA, GLNS, GLYQ, ASNC, SERS
SP_PIR_KEYWORDS	protein biosynthesis	PRFA, SELA, GLNS, GLYQ, ASNC, SELB, SERS
GOTERM_BP_FAT	GO:0006399~tRNA metabolic process	MIAA, YIHY, GLNS, RLUA, CCA, ASNA, GLYQ, QUEF, ASNC, SERS

Annotation Cluster 60	Enrichment Score: 1.3959919806272567	
Category	Term	Genes
GOTERM_BP_FAT	GO:0046394~carboxylic acid biosynthetic process	SERS, METL, FABI, GLTK, GLTL, FABG, GLNQ, GLTI, ASNA, FABR, GLNP, ASNC, GLNL, GLNG, GLNH, KDSA, MALY, GLNA, FABD, DKGB, DKGA, GLYA, KDSB, ACPP, DAPD, DAPB, FOLP, BIOH, GMHB, MTN, FOLD
GOTERM_BP_FAT	GO:0009309~amine biosynthetic process	GLNG, GLNH, MALY, GLNA, GLYA, SERS, DAPD, METL, DAPB, SPEB, GLTK, GLTL, FOLP, GSP, GLNQ, GLTI, GMHB, ASNA, GLNP, MTN, ASNC, FOLD, GLNL
GOTERM_BP_FAT	GO:0008652~cellular amino acid biosynthetic process	GLNG, GLNH, MALY, GLNA, GLYA, SERS, DAPD, METL, DAPB, GLTK, GLTL, FOLP, GLNQ, GLTI, GMHB, ASNA, GLNP, MTN, ASNC, FOLD, GLNL

Annotation Cluster 61	Enrichment Score: 1.3872852196421224	
Category	Term	Genes
SP_PIR_KEYWORDS	purine nucleotide biosynthesis	PURH, PURT, PURN, PURC, PURB, PURA
SP_PIR_KEYWORDS	purine biosynthesis	PURH, PURT, PURF, PURN, PURC, PURB, FOLD, PURA
GOTERM_BP_FAT	GO:0034654~nucleobase, nucleoside, nucleotide and nucleic acid biosynthetic process	PURH, PURT, NADC, PURF, UPP, NADE, MELA, PURN, HPT, QUEF, PURC, RFAD, PURB, PURA, GRXC, GALE, PANE, FOLD, ADD

Annotation Cluster 62	Enrichment Score: 1.3787749734931467	
Category	Term	Genes

GOTERM_BP_FAT	GO:0015768~maltose transport	MALK, LAMB, MALE
GOTERM_BP_FAT	GO:0015766~disaccharide transport	MALK, LAMB, MALE
GOTERM_BP_FAT	GO:0042946~glucoside transport	MALK, LAMB, MALE

Annotation Cluster 63	Enrichment Score: 1.377420886422273	
Category	Term	Genes
KEGG_PATHWAY	eco00760:Nicotinate and nicotinamide metabolism	USHA, NADC, NADE, DEOD, NADK

Annotation Cluster 64	Enrichment Score: 1.3558788095008694	
Category	Term	Genes
SP_PIR_KEYWORDS	Flavoprotein	PDXH, NFSB, HCR, AHPF, LPD, GLPD, HMP, MDAB, YQCA, NIRB, GOR, TRXB, NEMA, AZOR, UBIF
SP_PIR_KEYWORDS	FAD	NIRB, HCR, GOR, AHPF, LPD, GLPD, HMP, TRXB, UBIF
GOTERM_MF_FAT	GO:0016667~oxidoreductase activity, acting on sulfur group of donors	GRXB, GRXC, GRXD, GOR, AHPF, DSBA, DSBB, LPD, TRXB
INTERPRO	IPR001327:Pyridine nucleotide-disulphide oxidoreductase, NAD-binding region	NIRB, GOR, AHPF, LPD, TRXB
GOTERM_MF_FAT	GO:0016668~oxidoreductase activity, acting on sulfur group of donors, NAD or NADP as acceptor	GOR, LPD, TRXB
UP_SEQ_FEATURE	nucleotide phosphate-binding region:FAD	NIRB, GOR, AHPF, LPD, GLPD, HMP, TRXB
INTERPRO	IPR013027:FAD-dependent pyridine nucleotide-disulphide oxidoreductase	NIRB, GOR, AHPF, LPD, TRXB
GOTERM_MF_FAT	GO:0016651~oxidoreductase activity, acting on NADH or NADPH	GOR, AHPF, LPD, TRXB, MDAB, QUEF, AZOR, HYFG
GOTERM_MF_FAT	GO:0050660~FAD binding	NIRB, GOR, AHPF, LPD, TRXB, UBIF
