# Supplementary material for: Central role for RNase YbeY in Hfq-dependent and Hfq-independent small-RNA regulation in bacteria
Source: BMC Genomics. 2014 Feb 11;15:121. doi: 10.1186/1471-2164-15-121 (PMC3933206; doi:10.1186/1471-2164-15-121)
Supplement: Additional file 8: Table S8 — Functional clusters of genes differentially regulated upon HU exposure only in YbeY’s absence. [file 1471-2164-15-121-S8.pdf]

Supplemental Table 8. Functional clusters of genes differentially regulated upon HU exposure only in YbeY's absence

Annotation Cluster 1	Enrichment Score: 39.31374938791406	
Category	Term	Genes
SP_PIR_KEYWORDS	cell inner membrane	YDJZ, QSEC, CHAA, YOHD, YQAA, PITB, TRKG, YJGN, YNAI, YPHD, YBBM, EAMB, MNTH, MRCA, SETB, ACRB, NIKE, YOAE, YGBN, PANF, ACRD, YEDV, SRLE, YGDD, YAJR, GNTU, RHTA, SRLA, WBBK, YHHT, PLDB, YEDE, SANA, YBJG, YHHQ, MRDB, YGCS, ARGK, RSXD, RSXC, RSXB, YCCS, RSXA, MDOB, YDDG, YDDA, YNFA, FRUA, YNFH, KDGT, PTSG, LLDP, YRFF, YQJF, MDOH, MANY, MANX, YHAI, SETA, MANZ, CMR, BTUD, YJHF, BTUC, YCIS, PHNC, EMRB, DGOT, WZC, CCMD, SAPF, YCIB, YCIC, YTFL, SAPD, YCAM, PPID, SAPB, SAPC, TORS, CYOA, CCME, YCFZ, YIHN, YIHO, YIFK, NARQ, TDCC, FRDD, DCUC, FRDC, FRWC, NARX, YHBE, YIHP, AER, MGLC, DCUS, ZNTA, SSUC, MDTG, YIHG, MDTH, MDTK, DINF, MDTI, MDTJ, LSRD, CBRB, GSPO, GLPA, GSPK, YMFA, GLPG, XYLE, SPPA, XYLG, YIHY, CYDC, CYDD, MODB, LOLD, LOLC, MODF, TEHA, YDGI, LOLE, YDGK, TOLR, TOLQ, YIJP, YECN, YIDK, YFCJ, DJLA, FTSN, YNJC, FRYC, MIND, YDHU, YDHC, YNJF, SBMA, YJEH, NFRB, YIJD, YBHS, CREC, YBHQ, DMSD, DMSC, YQFA, RFAL, DSBB, YBAL, SECY, AGAC, AGAD, YGJV, BAES, YCEJ, KEFA, YFAV, YBIP, YHJV, DEDA, HISM, YBIR, KEFB
SP_PIR_KEYWORDS	cell membrane	YDJZ, YDJX, YQAA, TRKG, WZA, YNAI, YBBM, EAMB, ACRB, NIKE, YGBN, YOAE, PANF, ACRD, SRLE, GNTU, SRLA, RHTA, PLDB, PLDA, SANA, YGCG, YGCS, ARGK, KDGT, PTSG, LLDP, MANY, MANX, YHAI, MANZ, YCIS, PHNC, PHNE, WZC, DGOT, SAPF, YCIB, YCIC, SAPD, SAPB, SAPC, CYOA, YCFZ, YIHN, MDTP, YIHO, YIFK, NARQ, FRDD, FRDC, NARX, YHBE, YIHP, AER, SSUC, YIHG, MDTG, SLYB, MDTH, MDTK, DINF, MDTI, MDTJ, LSRD, CBRB, GSPO, GSPK, YMFA, YIHY, MODB, LOLD, LOLC, YDGI, TEHA, MODF, LOLE, YDGK, YIJP, YIDK, APBE, DJLA, FTSN, YNJC, YDHU, YDHC, SBMA, YNJF, NFRB, NFRA, YIJD, YBHS, CREC, YBHQ, YQFA, RFAL, BAES, YCEJ, MLTB, MLTC, YBIP, YHDV, YBIR, QSEC, YOHD, CHAA, PITB, YJGN, YPHD, MRCA, MNTH, SETB, SPR, MIPA, YEDV, YEDQ, YAJR, YGDD, WBBK, YHHT, YNIB, YBJE, YEDE, YBJG, YHHQ, MRDB, RSXD, RSXC, RSXB, YCCS, RSXA, MDOC, MDOB, YDDG, YDDA, YNFA, FRUA, YNFE, YNFF, YNFH, YDDW, YRFF, YQJF, MDOH, YFGH, YJIK, SETA, CMR, BTUD, BTUC, YJHF, YDEE, EMRB, CCMD, YTFL, YCAM, PPID, TORS, OMPW, YGFQ, LEUE, YAHG, FLU, YHFK, CCME, YEAJ, TDCC, DCUC, FRWC, MGLC, DCUS, ZNTA, GLPA, GLPG, XYLE, SPPA, XYLG, YJBF, CYDC, CYDD, TOLR, TOLQ, YRAJ, YECN, YNBD, YFCJ, FRYC, MIND, YRAQ, YJEH, DMSD, DMSC, DSBB, YBAL, SECY, AGAC, AGAD, YGJV, YEAY, KEFA, YFAV, YHJV, HISM, DEDA, KEFB

Annotation Cluster 2	Enrichment Score: 13.424449217862357	
Category	Term	Genes
SP_PIR_KEYWORDS	phosphotransferase	MANZ, CHBB, CHBA, NARQ, TDCD, PYKF, SRLE, NARX, SRLA, SRLB, FRUA, FRYC, PTSI, PTSG, TDK, TORS, COAA, PRKB, PYKA, MANY, CREC, MANX, PFKA, UDK
SP_PIR_KEYWORDS	phosphoprotein	QSEC, NARQ, NARX, USPA, FRUA, FRYC, PTSI, CYAA, PTSG, DCUR, DCUS, ZNTA, FIMZ, CPXR, NARL, TDCB, CREC, MANX, CHBB, YEDV, CHBA, SRLE, SRLA, WZC, SRLB, NPR, BAES, TORS

SP_PIR_KEYWORDS	kinase	QSEC, NARQ, TDCD, FRWD, NARX, YGGC, FRUA, NAGK, PTSI, PTSG, DCUS, FRLD, RFAP, PYKA, MANX, CREC, YEDV, CHBB, PYKF, SRLE, WZC, SRLB, YBCL, NPR, TDK, YGCE, BAES, TORS, COAA, ARGK, PRKB, SELD, PFKA, UDK

Annotation Cluster 3	Enrichment Score: 7.56255043486997	
Category	Term	Genes
SP_PIR_KEYWORDS	phosphotransferase	MANZ, CHBB, CHBA, NARQ, TDCD, PYKF, SRLE, NARX, SRLA, SRLB, FRUA, FRYC, PTSI, PTSG, TDK, TORS, COAA, PRKB, PYKA, MANY, CREC, MANX, PFKA, UDK
KEGG_PATHWAY	ecd00051:Fructose and mannose metabolism	MANZ, TPIA, MANA, SRLE, GMD, FBP, SRLA, FRUA, SRLB, FCL, CPSB, MANY, MANX, PFKA
SP_PIR_KEYWORDS	sugar transport	FRWC, FRWD, FRUA, FRYC, PTSI, KDGT, WZA, MGLC, PTSG, YPHD, SETB, MANY, MANX, CHBB, MANZ, SETA, CHBA, SRLE, GNTU, SRLA, SRLB, AGAC, AGAD, XYLE, XYLG
GOTERM_BP_FAT	GO:0046377~colanic acid metabolic process	RCSA, FCL, WZA, WCAI, WZB, MANA, GMD, GALU, CPSB, WCAA, WZC, WCAB
KEGG_PATHWAY	ecd00520:Amino sugar and nucleotide sugar metabolism	MANZ, FCL, PTSG, MANA, GMD, GALU, CPSB, NAGK, MANY, MANX, NAGZ
GOTERM_BP_FAT	GO:0009401~phosphoenolpyruvate-dependent sugar phosphotransferase system	CHBB, MANZ, CHBA, FRWC, SRLE, FRWD, SRLA, SRLB, FRUA, AGAC, FRYC, AGAD, PTSI, PTSG, NPR, SRLR, GUTM, MANY, MANX

Annotation Cluster 4	Enrichment Score: 7.1384733842949135	
Category	Term	Genes
GOTERM_BP_FAT	GO:0009061~anaerobic respiration	TDCE, NARQ, FRDD, PYKF, FRDC, GLPA, FRDB, NARX, NAPB, HYFG, YNFH, ACEE, DCUR, DCUS, TORS, YFID, LLDD, NIKE, PFLA, NARL, PYKA, PFLC, DMSC
GOTERM_BP_FAT	GO:0045333~cellular respiration	TDCE, NARQ, FRDD, FRDC, FRDB, NARX, NAPB, YDHU, YNFH, ACEE, MNGR, DCUR, DCUS, YFID, LLDD, NIKE, PFLA, MQO, NARL, PYKA, PFLC, DMSC, PYKF, GLPA, UBIX, HYFG, YCEJ, TORS, CYOA
GOTERM_BP_FAT	GO:0006091~generation of precursor metabolites and energy	TDCE, NARQ, TPIA, FRDD, FRDC, FRDB, NARX, NAPB, YNFG, YDHU, YNFH, ACEE, MNGR, DCUR, DCUS, ZNTA, YFID, LLDD, PFLA, NIKE, MQO, NARL, PYKA, PFLC, DMSC, YHJA, PYKF, GLPA, UBIX, DSBB, HYFG, GRXD, YCEJ, TORS, YGFS, HCAC, RSXD, TRXC, YGCR, RSXC, RSXB, CYOA, RSXA, PFKA

Annotation Cluster 5	Enrichment Score: 6.64474218328045	
Category	Term	Genes
SP_PIR_KEYWORDS	lipopolysaccharide biosynthesis	RFAG, YIJP, KDSA, RFAL, GMD, WCAA, RFBA, WCAB, RFBB, RFAC, RFBC, WBBK, RFBD, RFAB, FCL, WCAI, RFAP, CPSB, RFAS
GOTERM_BP_FAT	GO:0016051~carbohydrate biosynthetic process	MDOC, MDOB, TPIA, MDOG, MANA, TDCG, RFBA, RFBB, RFBC, HIPA, RFBD, HIPB, LDCA, WZA, FCL, WZB, NAGC, MRCA, RFAP, MDOH, RFAS, MIPA, RFAG, RCSA, KDSA, RFAL, YEDQ, GMD, GALU, FBP, WCAA, WZC, DDLA, WCAB, RFAC, DACB, AGAD, WBBK, RFAB, WCAI, YBJG, MURF, AMPC, MLTB, AMIB, CPSB, MLTC, NAGZ

GOTERM_BP_FAT	GO:0000271~polysaccharide biosynthetic process	MDOC, MDOB, MDOG, MANA, RFBA, RFBB, RFBC, HIPA, RFBD, HIPB, LDCA, WZA, FCL, WZB, MRCA, RFAP, MDOH, RFAS, MIPA, RCSA, RFAG, KDSA, RFAL, YEDQ, GMD, GALU, WCAA, WZC, DDLA, WCAB, RFAC, DACB, WBBK, RFAB, WCAI, YBJG, MURF, AMPC, MLTB, AMIB, CPSB, MLTC, NAGZ

Annotation Cluster 6	Enrichment Score: 6.16768996954552	
Category	Term	Genes
SP_PIR_KEYWORDS	ATP	BTUD, PHNC, SRMB, ASNS, MIND, NAGK, YNJD, SERS, ARGS, SAPF, HELD, MURF, XYLG, SAPD, TDK, CYDC, CYDD, LOLD, NIKE, MODF, PFKA
SP_PIR_KEYWORDS	nucleotide binding	BTUD, PHNC, SRMB, MIND, NAGK, YNJD, OBGE, SAPF, HELD, MURF, XYLG, SAPD, TDK, CYDC, CYDD, NFRA, LOLD, YCHF, NIKE, MODF
SP_PIR_KEYWORDS	P-loop	BTUD, PHNC, SRMB, MIND, NAGK, YNJD, OBGE, SAPF, HELD, MURF, XYLG, SAPD, TDK, CYDC, CYDD, NFRA, LOLD, YCHF, NIKE, MODF
KEGG_PATHWAY	eco02010:ABC transporters	YDDA, MGLC, YGIS, SSUC, NIKE, YBHS, BTUD, BTUC, LSRD, PHNC, PHNE, PHND, SAPF, SAPD, XYLG, CYDC, SAPB, CYDD, SAPC, MODB, LOLD, LOLC, MODF, LOLE, HISM, POTD
GOTERM_CC_FAT	GO:0019898~extrinsic to membrane	BTUD, BTUC, GLPA, PHNC, MIND, WBBK, PLDB, SAPF, XYLG, SAPD, ARGK, LOLD, FLU, RSXC, NIKE, RSXB, MODF, DMSD, MANX
INTERPRO	IPR017871:ABC transporter, conserved site	BTUD, BTUC, YDDA, PHNC, RFBB, YNJD, YBBL, SAPF, XYLG, SAPD, CYDC, CYDD, LOLD, NIKE, MODF
GOTERM_MF_FAT	GO:0042623~ATPase activity, coupled	BTUD, BTUC, YDDA, SRMB, PHNC, MIND, MGLC, SBMA, HELD, XYLG, CYDC, CYDD, ZNTA, RHLB, RECQ, YBHS, NIKE
UP_SEQ_FEATURE	nucleotide phosphate-binding region:ATP	YDDA, SRMB, YGGC, NAGK, MIND, YNJD, SERS, YBBL, YGGR, RECQ, NIKE, BTUD, BTUC, PHNC, DDLA, TYRR, GUAA, SAPF, HELD, TDK, SAPD, XYLG, MURF, CYDC, RIMK, CYDD, MUTS, RHLB, COAA, ARGK, LOLD, YGFQ, PRKB, SELD, PFKA, UDK
INTERPRO	IPR003593:ATPase, AAA+ type, core	BTUD, BTUC, YDDA, PHNC, TYRR, RFBB, YNJD, YBBL, SAPF, XYLG, SAPD, CYDC, CYDD, YGGR, LOLD, NIKE, MODF, FFH
UP_SEQ_FEATURE	domain:ABC transmembrane type-1	YDDA, CYDC, SAPB, CYDD, SSUC, SAPC, MODB, HISM
GOTERM_MF_FAT	GO:0016887~ATPase activity	BTUD, BTUC, YDDA, PHNC, SRMB, RFBB, MIND, YNJD, YBBL, MGLC, SAPF, SBMA, HELD, XYLG, SAPD, CYDC, CYDD, ZNTA, RHLB, RECQ, LOLD, NIKE, YBHS, MODF
SMART	SM00382:AAA	BTUD, BTUC, YDDA, PHNC, TYRR, RFBB, YNJD, YBBL, SAPF, XYLG, SAPD, CYDC, CYDD, YGGR, LOLD, NIKE, MODF, FFH

Annotation Cluster 7	Enrichment Score: 5.248808349173658	
Category	Term	Genes
SP_PIR_KEYWORDS	DNA binding	YCJW, HUPA, ADIY, SFSB, DEOR, CYNR, CSPF, YCJZ, ICLR, HIPB, METJ, KDGR, MNGR, NAGC, FADR, MALI, CYTR, SRLR, SLYA, NARL, RCSA, RPOD, LEXA, POLA, LRP, GLPG, HELD, LYSR, PHNF, YCGE, IHFA, GUTM, YCIM
SP_PIR_KEYWORDS	repressor	ENVR, YFET, DEOR, CYNR, ICLR, HIPB, METJ, MNGR, NAGC, FADR, MALI, ULAR, CYTR, SRLR, SLYA, NARL, MNTR, RPSD, LSRR, GNTR, YEBK, LEXA, TYRR, CHBR, PAAX, LYSR

SP_PIR_KEYWORDS	dna-binding	YCJW, YFET, HUPA, ADIY, SFSB, CYNR, CSPF, ICLR, YCJZ, YEIE, METJ, DCUR, MNGR, MALI, FIMZ, SRLR, RECQ, SLYA, NARL, MNTR, RCSA, GNTR, LEXA, POLA, TYRR, GLCC, LRP, CHBR, YLBG, PAAX, LYSR, YCGE, GUTM, YDCN, EUTR, CEDA, YBJK, YJIE, ENVR, YJJQ, YAIW, DEOR, YIDL, YGGD, HIPA, HIPB, OBGE, YBDO, YDHB, KDGR, YJIR, NAGC, FADR, ULAR, CYTR, CPXR, LSRR, RPOD, YBAQ, YDIP, YEBK, INSG, ABGR, HELD, PHNF, IHFA, MUTS, ALPA, YFAX
SP_PIR_KEYWORDS	Transcription	YCJW, YFET, ADIY, SFSB, CYNR, CSPF, ICLR, YCJZ, YEIE, METJ, DCUR, MNGR, MALI, FIMZ, SRLR, SLYA, NARL, MNTR, RCSA, GNTR, LEXA, TYRR, GLCC, LRP, RSD, CHBR, PAAX, LYSR, YCGE, GUTM, YDCN, EUTR, YBJK, YJIE, ENVR, YJJQ, DEOR, YIDL, HIPB, YDHB, YBDO, KDGR, NAGC, YJIR, FADR, ULAR, CYTR, CPXR, LSRR, RPSD, RPOD, YDIP, YEBK, YBAQ, RPOA, ABGR, PHNF, IHFA, ALPA, YFAX
SP_PIR_KEYWORDS	transcription regulation	YCJW, YFET, ADIY, SFSB, CYNR, CSPF, ICLR, YCJZ, YEIE, METJ, DCUR, MNGR, MALI, FIMZ, SRLR, SLYA, NARL, MNTR, RCSA, GNTR, LEXA, TYRR, GLCC, LRP, RSD, CHBR, PAAX, LYSR, YCGE, GUTM, YDCN, EUTR, YBJK, YJIE, ENVR, YJJQ, DEOR, YIDL, HIPB, YDHB, YBDO, KDGR, NAGC, YJIR, FADR, ULAR, CYTR, CPXR, LSRR, RPSD, RPOD, YDIP, YEBK, YBAQ, ABGR, PHNF, IHFA, ALPA, YFAX

Annotation Cluster 8	Enrichment Score: 4.8108101673059895	
Category	Term	Genes
SP_PIR_KEYWORDS	metal-binding	FRDB, TDCG, SCPA, YDJJ, ACEE, ZNTA, YPHC, GLTD, PFLA, YGBM, PFLC, CUTA, YACG, YPDE, CHBF, YCGM, HYFG, PLDA, RIMK, RSXC, TRXC, RSXB, YBJI, PFKA, PTRA, TYNA, MANA, YBHJ, YDHY, RFBA, YJJN, GLOA, NAPB, YNFE, NAGK, YNFF, YDHX, YNFG, PTSI, NUDF, SPEA, NUDL, DGOD, YCHF, YIDA, PYKA, YHJA, RIHB, YGJD, PYKF, VSR, FBP, DDLA, YCAL, YCEJ, YGFS, HCAC, YDEM, MUTY, YCIM, CCME, YGFT
GOTERM_MF_FAT	GO:0043169~cation binding	CHAA, FRDB, TDCG, SCPA, EXOX, TRKG, YDJJ, ACEE, ZNTA, MNTH, YPHC, GLTD, PFLA, NIKE, MDTK, CUTC, PFLC, YGBM, MNTR, PANF, CUTA, YIIM, RECJ, YACG, CHBF, YCGM, HYFG, TORZ, PYRE, PLDA, RIMK, MODB, RSXC, TRXC, RSXB, MODF, YBJI, PFKA, PTRA, TYNA, MANA, YBHJ, YIDK, YDHY, RFBA, YJJN, NAPB, EBGA, GLOA, NAGK, YNFE, YNFF, YDHX, YNFG, PTSI, NUDF, SPEA, YDDW, NUDK, NUDL, DGOD, YCHF, PYKA, YIDA, YGGX, YHJA, BTUE, RIHB, YGJD, PYKF, YHJJ, GALU, VSR, FBP, DDLA, YCAL, YCEJ, YGFS, HCAC, BGLA, YDEM, SELD, CYOA, KEFA, MUTY, CCME, YCIM, YGFT, KEFB

Annotation Cluster 9	Enrichment Score: 4.572412933376043	
Category	Term	Genes
GOTERM_BP_FAT	GO:0042493~response to drug	CMR, ENVR, YAJR, EMRB, DACB, YGEA, SBMA, SANA, YBBM, AMPC, MRCA, MDTG, ACRB, MDTK, TEHA, DINF, TEHB, ACRD
SP_PIR_KEYWORDS	antibiotic resistance	MDTP, RPSD, EMRB, DACB, RPLV, YBJG, AMPC, MRCA, MDTG, MDTH, TEHA, MDTK, TEHB
GOTERM_BP_FAT	GO:0046677~response to antibiotic	MDTP, RPSD, EMRB, DACB, RPLV, YBJG, AMPC, MRCA, MDTG, MDTH, TEHA, MDTK, TEHB

Annotation Cluster 10	Enrichment Score: 4.358784751883762	
Category	Term	Genes
SP_PIR_KEYWORDS	electron transport	YHJA, FRDB, DSBB, NAPB, YNFG, GRXD, YCEJ, HCAC, YGFS, RSXD, TRXC, RSXC, YGCR, CYOA, RSXB, RSXA
GOTERM_BP_FAT	GO:0006091~generation of precursor metabolites and energy	TDCE, NARQ, TPIA, FRDD, FRDC, FRDB, NARX, NAPB, YNFG, YDHU, YNFH, ACEE, MNGR, DCUR, DCUS, ZNTA, YFID, LLDD, PFLA, NIKE, MQO, NARL, PYKA, PFLC, DMSC, YHJA, PYKF, GLPA, UBIX, DSBB, HYFG, GRXD, YCEJ, TORS, YGFS, HCAC, RSXD, TRXC, YGCR, RSXC, RSXB, CYOA, RSXA, PFKA
GOTERM_BP_FAT	GO:0022900~electron transport chain	YHJA, FRDB, DSBB, NAPB, YNFG, YDHU, YNFH, GRXD, YCEJ, HCAC, YGFS, RSXD, TRXC, YGCR, RSXC, CYOA, RSXB, RSXA, DMSC
GOTERM_MF_FAT	GO:0009055~electron carrier activity	FRDB, YDHY, YNFE, YNFF, YDHX, YNFG, YDHU, YDHV, GLTD, LLDD, YDIS, YHJA, AHPF, DSBB, HYFG, TORZ, GRXD, YCEJ, YCAK, HCAC, YGFS, TRXB, TRXC, YGCR, HCAD, RSXC, CYOA, RSXB, YDGJ, YGFT

Annotation Cluster 11	Enrichment Score: 4.304510052100273	
Category	Term	Genes
SP_PIR_KEYWORDS	protein biosynthesis	RPMJ, RPSD, RPSC, ASNS, SERS, RPMD, ARGS, RPLW, RPSS, RPLV, RPSM, RIMK, GLYS, RPSJ, GLYQ, RPSK
SP_PIR_KEYWORDS	ribosome	RPMJ, RPLW, RPSS, RPSD, RPSC, RPLV, RPSM, RPSJ, RPSK, RPMD
SP_PIR_KEYWORDS	ribonucleoprotein	RPMJ, RPLW, RPSS, RPSD, RPSC, RPLV, RPSM, RPSJ, RPSK, FFH, RPMD
GOTERM_CC_FAT	GO:0015935~small ribosomal subunit	RPSS, RPSD, RPSC, RPSJ
GOTERM_MF_FAT	GO:0003723~RNA binding	RPSD, RPSC, RNR, SRMB, RLML, OBGE, RPLW, RPSS, RPLV, RPSM, RLUE, SMPB, RHLB, RPSJ, RPSK, TRUA, FFH
GOTERM_MF_FAT	GO:0000049~tRNA binding	RPSS, RPSM, RPSJ

Annotation Cluster 12	Enrichment Score: 3.9113865374882444	
Category	Term	Genes
SP_PIR_KEYWORDS	magnesium	EBGA, RFBA, EXOX, PTSI, NUDF, ACEE, SPEA, ZNTA, NUDK, NUDL, DGOD, YCHF, RFAP, YIDA, PYKA, PYKF, GALU, VSR, FBP, DDLA, PYRE, RIMK, SELD, YBJI, PFKA, PTRA

Annotation Cluster 13	Enrichment Score: 3.7422686075689926	
Category	Term	Genes
SP_PIR_KEYWORDS	phosphoprotein	QSEC, NARQ, NARX, USPA, FRUA, FRYC, PTSI, CYAA, PTSG, DCUR, DCUS, ZNTA, FIMZ, CPXR, NARL, TDCB, CREC, MANX, CHBB, YEDV, CHBA, SRLE, SRLA, WZC, SRLB, NPR, BAES, TORS
SP_PIR_KEYWORDS	phosphohistidine	PTSI, NARQ, NPR, DCUS, TORS, NARX, SRLB, MANX, CREC

KEGG_PATHWAY	ecw02020:Two-component system	RCSA, QSEC, NARQ, FRDD, FRDC, FRDB, NARX, AER, DCUR, DCUS, BAES, AMPC, TORS, FIMZ, CPXR, NARL, CREC
SP_PIR_KEYWORDS	autophosphorylation	NARQ, DCUS, NARX, CREC
INTERPRO	IPR003660:HAMP linker domain	YEDV, QSEC, AER, NARQ, BAES, TORS, NARX, CREC
UP_SEQ_FEATURE	domain:Histidine kinase	YEDV, QSEC, NARQ, DCUS, BAES, TORS, NARX, CREC
INTERPRO	IPR005467:Signal transduction histidine kinase, core	YEDV, QSEC, NARQ, DCUS, BAES, TORS, NARX, CREC
SMART	SM00388:HisKA	YEDV, QSEC, HELD, BAES, TORS, NARX, CREC
SMART	SM00387:HATPase_c	YEDV, QSEC, NARQ, DCUS, BAES, TORS, NARX, CREC
GOTERM_BP_FAT	GO:0000160~two-component signal transduction system (phosphorelay)	RCSA, YEDV, QSEC, NARQ, YJJQ, NARX, TYRR, YEGE, DCUR, DCUS, BAES, TORS, FIMZ, CPXR, NARL, CREC

Annotation Cluster 14	Enrichment Score: 3.7165808580986788	
Category	Term	Genes
KEGG_PATHWAY	ect00230:Purine metabolism	CYAA, NUDF, RIHB, GUAA, PYKF, HOLA, RPOA, POLA, HOLE, PYKA, ADD

Annotation Cluster 15	Enrichment Score: 3.4138636665047963	
Category	Term	Genes
SP_PIR_KEYWORDS	nucleotide-binding	QSEC, NARQ, TDCD, SRMB, NARX, YBBL, DCUS, ZNTA, FRLD, RECQ, NIKE, NARL, YEDV, TYRR, ARGS, XYLG, MURF, CYDC, RIMK, YAJQ, CYDD, RHLB, GLYS, ARGK, GLYQ, LOLD, PRKB, MODF, PFKA, YDDA, ASNS, YGGC, MIND, HSCC, RFBB, NAGK, YNJD, SERS, CYAA, OBGE, YGGR, YCHF, RFAP, PYKA, CREC, RTCA, BTUD, NADD, BTUC, PYKF, PHNC, FBP, WZC, DDLA, SAPF, GUAA, HELD, SAPD, TDK, BAES, TORS, MUTS, COAA, CPSB, YGFQ, SELD, FFH, UDK
SP_PIR_KEYWORDS	atp-binding	QSEC, NARQ, TDCD, SRMB, NARX, YBBL, DCUS, ZNTA, FRLD, RECQ, NIKE, NARL, YEDV, TYRR, ARGS, MURF, XYLG, CYDC, RIMK, CYDD, RHLB, GLYS, ARGK, GLYQ, LOLD, PRKB, MODF, PFKA, YDDA, ASNS, YGGC, MIND, HSCC, RFBB, NAGK, YNJD, SERS, CYAA, USPF, YGGR, RFAP, PYKA, CREC, RTCA, BTUD, NADD, BTUC, PYKF, PHNC, WZC, DDLA, GUAA, SAPF, HELD, SAPD, TDK, BAES, TORS, MUTS, COAA, YGFQ, SELD, UDK
GOTERM_MF_FAT	GO:0005524~ATP binding	QSEC, NARQ, TDCD, SRMB, NARX, YBBL, DCUS, ZNTA, FRLD, RECQ, NIKE, NARL, YEDV, TYRR, ARGS, MURF, XYLG, CYDC, RIMK, CYDD, RHLB, GLYS, ARGK, GLYQ, LOLD, PRKB, MODF, PFKA, YDDA, ASNS, YGGC, MIND, HSCC, RFBB, NAGK, YNJD, SERS, CYAA, USPF, SBMA, NFRB, YGGR, RFAP, YBHS, PYKA, CREC, RTCA, BTUD, NADD, BTUC, PYKF, PHNC, WZC, DDLA, SAPF, GUAA, HELD, SAPD, TDK, BAES, TORS, MUTS, COAA, YGFQ, SELD, UDK

GOTERM_MF_FAT	GO:0032553~ribonucleotide binding	QSEC, NARQ, TDCD, SRMB, NARX, YBBL, DCUS, ZNTA, FRLD, RECQ, NIKE, NARL, YEDV, TYRR, ARGS, XYLG, MURF, CYDC, RIMK, CYDD, RHLB, GLYS, ARGK, GLYQ, LOLD, PRKB, MODF, PFKA, YDDA, ASNS, YGGC, MIND, HSCC, RFBB, NAGK, YNJD, SERS, CYAA, USPF, OBGE, SBMA, NFRB, YGGR, YCHF, RFAP, YBHS, PYKA, CREC, RTCA, BTUD, BTUC, NADD, PYKF, PHNC, WZC, DDLA, SAPF, GUAA, HELD, SAPD, TDK, BAES, TORS, MUTS, COAA, CPSB, YGFQ, SELD, FFH, UDK
GOTERM_MF_FAT	GO:0032555~purine ribonucleotide binding	QSEC, NARQ, TDCD, SRMB, NARX, YBBL, DCUS, ZNTA, FRLD, RECQ, NIKE, NARL, YEDV, TYRR, ARGS, XYLG, MURF, CYDC, RIMK, CYDD, RHLB, GLYS, ARGK, GLYQ, LOLD, PRKB, MODF, PFKA, YDDA, ASNS, YGGC, MIND, HSCC, RFBB, NAGK, YNJD, SERS, CYAA, USPF, OBGE, SBMA, NFRB, YGGR, YCHF, RFAP, YBHS, PYKA, CREC, RTCA, BTUD, BTUC, NADD, PYKF, PHNC, WZC, DDLA, SAPF, GUAA, HELD, SAPD, TDK, BAES, TORS, MUTS, COAA, CPSB, YGFQ, SELD, FFH, UDK
GOTERM_MF_FAT	GO:0000166~nucleotide binding	QSEC, NARQ, TDCD, SRMB, NARX, YBBL, DCUS, ZNTA, FRLD, RECQ, GLTD, NIKE, NARL, PANE, YEDV, PDXH, GLPA, AHPF, YGCU, TYRR, HYFG, ARGS, RPLW, XYLG, MURF, YCGE, CYDC, RIMK, YAJQ, CYDD, RHLB, GLYS, ARGK, TRXB, GLYQ, LOLD, PRKB, RSXC, MODF, PFKA, YDDA, GND, ASNS, YGGC, NAGK, MIND, HSCC, RFBB, YRAR, YNJD, SERS, CYAA, USPF, OBGE, SBMA, NFRB, YGGR, YCHF, RFAP, LLDD, YBHS, PYKA, CREC, FADJ, RTCA, BTUD, BTUC, NADD, PYKF, PHNC, FBP, WZC, DDLA, SAPF, GUAA, HELD, TDK, SAPD, BAES, MUTS, TORS, COAA, YGFQ, CPSB, HCAD, SELD, FFH, YGFT, UDK
UP_SEQ_FEATURE	nucleotide phosphate-binding region:ATP	YDDA, SRMB, YGGC, NAGK, MIND, YNJD, SERS, YBBL, YGGR, RECQ, NIKE, BTUD, BTUC, PHNC, DDLA, TYRR, GUAA, SAPF, HELD, TDK, SAPD, XYLG, MURF, CYDC, RIMK, CYDD, MUTS, RHLB, COAA, ARGK, LOLD, YGFQ, PRKB, SELD, PFKA, UDK

Annotation Cluster 16	Enrichment Score: 3.3724274305760975	
Category	Term	Genes
GOTERM_BP_FAT	GO:0009226~nucleotide-sugar biosynthetic process	RIHA, MANA, CPSB, RFBA, RFBB, RFBC, RFBD
GOTERM_BP_FAT	GO:0009225~nucleotide-sugar metabolic process	RIHA, MANA, GMD, GALU, CPSB, RFBA, RFBB, RFBC, RFBD
KEGG_PATHWAY	ecj00523:Polyketide sugar unit biosynthesis	RFBA, RFBB, RFBC, RFBD

Annotation Cluster 17	Enrichment Score: 3.136465816777248	
Category	Term	Genes
KEGG_PATHWAY	ecd00010:Glycolysis / Gluconeogenesis	PTSG, ACEE, TPIA, PYKF, BGLA, FBP, CHBF, PYKA, PFKA
GOTERM_BP_FAT	GO:0019318~hexose metabolic process	TDCE, TPIA, PYKF, GND, TDCG, GMD, GALU, FBP, RFBA, YIHU, RFBC, YIHS, YGBJ, ACEE, PRKB, PFLA, PYKA, FUCU, PFLC, PFKA
GOTERM_BP_FAT	GO:0005996~monosaccharide metabolic process	TDCE, TPIA, TDCG, GND, YIHU, RFBA, NAGK, YIHS, RFBC, YGBJ, ACEE, NAGC, PFLA, FUCU, PYKA, PFLC, KDSA, PYKF, GMD, GALU, AGAI, FBP, AGAD, PRKB, PFKA

GOTERM_BP_FAT	GO:0006098~pentose-phosphate shunt	YGBJ, TPIA, GND, YIHU
GOTERM_BP_FAT	GO:0006739~NADP metabolic process	YGBJ, TPIA, GND, YIHU
Annotation Cluster 18	Enrichment Score: 2.921562174540362	
Category	Term	Genes
GOTERM_BP_FAT	GO:0009252~peptidoglycan biosynthetic process	DDLA, DACB, HIPA, HIPB, LDCA, YBJG, MURF, AMPC, MRCA, MLTB, MLTC, AMIB, NAGZ, MIPA
SP_PIR_KEYWORDS	cell wall biogenesis/degradation	LDCA, YBJG, MURF, MRCA, MLTB, AMIB, MLTC, DDLA, DACB, NAGZ
SP_PIR_KEYWORDS	cell shape	LDCA, YBJG, MURF, MRDB, MRCA, DDLA, DACB, NAGZ
GOTERM_BP_FAT	GO:0030203~glycosaminoglycan metabolic process	ARRD, DDLA, NAGK, DACB, HIPA, HIPB, LDCA, YBJG, MURF, AMPC, MRCA, MLTB, AMIB, MLTC, NAGZ, MIPA
Annotation Cluster 19	Enrichment Score: 2.710071754846438	
Category	Term	Genes
KEGG_PATHWAY	ecd00620:Pyruvate metabolism	TDCE, ACEE, PYKF, LLDD, GLOA, PYKA, MQO
Annotation Cluster 20	Enrichment Score: 2.6107761139508745	
Category	Term	Genes
SP_PIR_KEYWORDS	sugar transport	FRWC, FRWD, FRUA, FRYC, PTSI, KDGT, WZA, MGLC, PTSG, YPHD, SETB, MANY, MANX, CHBB, MANZ, SETA, CHBA, SRLE, GNTU, SRLA, SRLB, AGAC, AGAD, XYLE, XYLG
SP_PIR_KEYWORDS	Phosphotransferase system	CHBB, MANZ, CHBA, FRWC, SRLE, FRWD, SRLA, SRLB, FRUA, AGAC, FRYC, AGAD, PTSI, PTSG, NPR, SRLR, GUTM, MANY, MANX
GOTERM_MF_FAT	GO:0008982~protein-N(PI)-phosphohistidine-sugar phosphotransferase activity	CHBB, MANZ, CHBA, FRWC, SRLE, FRWD, SRLA, SRLB, FRUA, AGAC, FRYC, AGAD, PTSI, PTSG, MANY, MANX
GOTERM_BP_FAT	GO:0008643~carbohydrate transport	FRWC, FRWD, FRUA, FRYC, PTSI, KDGT, WZA, MGLC, PTSG, YPHD, SETB, SRLR, MANY, FUCU, MANX, YGBN, SETA, CHBB, MANZ, CHBA, YJHF, SRLE, GNTU, SRLA, SRLB, AGAC, AGAD, XYLE, NPR, XYLG, GUTM
Annotation Cluster 21	Enrichment Score: 2.5140381163035124	
Category	Term	Genes
KEGG_PATHWAY	ect00240:Pyrimidine metabolism	PYRE, RIHB, TDK, HOLA, RPOA, TRXB, POLA, HOLE, UDK
KEGG_PATHWAY	ect03430:Mismatch repair	RECJ, HOLA, MUTS, HOLE, SBCB, EXOX
KEGG_PATHWAY	ecd03440:Homologous recombination	RECJ, DNAT, HOLA, POLA, HOLE

KEGG_PATHWAY	ecc03440:Homologous recombination	RECJ, DNAT, HOLA, HOLE
SP_PIR_KEYWORDS	dna replication	HELD, DNAT, HOLA, LEXA, POLA, HOLE
SP_PIR_KEYWORDS	dna-directed dna polymerase	HOLA, POLA, HOLE
KEGG_PATHWAY	ect03030:DNA replication	HOLA, POLA, HOLE
KEGG_PATHWAY	ecl00240:Pyrimidine metabolism	PYRE, RIHB, HOLA

Annotation Cluster 22	Enrichment Score: 2.4875679650870612	
Category	Term	Genes
KEGG_PATHWAY	ecd00052:Galactose metabolism	DGOD, GALU, AGAI, EBGA, AGAC, AGAD, PFKA

Annotation Cluster 23	Enrichment Score: 2.418579839790211	
Category	Term	Genes
KEGG_PATHWAY	ecd00360:Phenylalanine metabolism	TYNA, HCAF, HCAC, HCAB, HCAD
GOTERM_BP_FAT	GO:0019439~aromatic compound catabolic process	HCAF, HCAC, HCAB, TYRR, HCAD
GOTERM_MF_FAT	GO:0016708~oxidoreductase activity, acting on paired donors, with incorporation or reduction of molecular oxygen, NADH or NADPH as one donor, and incorporation of two atoms of oxygen into one donor	HCAF, HCAC, HCAD

Annotation Cluster 24	Enrichment Score: 2.28666317904145	
Category	Term	Genes
GOTERM_MF_FAT	GO:0042280~cell surface antigen activity, host-interacting	RFAG, RCSA, KDSA, RFAL, GMD, RFBA, RFAC, RFBB, RFAB, FCL, CPSB, RFAP, RFAS
KEGG_PATHWAY	eco00540:Lipopolysaccharide biosynthesis	RFAG, KDSA, RFAL, RFAP, RFAC, RFAB

Annotation Cluster 25	Enrichment Score: 2.0606768572433305	
Category	Term	Genes
SP_PIR_KEYWORDS	cell cycle	YCIB, ZAPA, MURF, FTSN, MIND, DACB, MINE, NAGZ, CEDA, MINC
SP_PIR_KEYWORDS	cell division	YCIB, ENVC, ZAPA, MURF, FTSN, MIND, DACB, MINE, NAGZ, CEDA, MINC

Annotation Cluster 26	Enrichment Score: 2.0169063214021836	
Category	Term	Genes

KEGG_PATHWAY	eck00650:Butanoate metabolism	TDCE, FRDD, ACEE, FRDC, FRDB, FADJ, PAAG
KEGG_PATHWAY	ecj00632:Benzoate degradation via CoA ligation	FRDD, FRDC, FRDB, FADJ, PAAG
KEGG_PATHWAY	eck00020:Citrate cycle (TCA cycle)	FRDD, ACEE, FRDC, YBHJ, FRDB
KEGG_PATHWAY	eck00640:Propanoate metabolism	TDCE, TDCD, FADJ, PAAG
KEGG_PATHWAY	ecv00190:Oxidative phosphorylation	FRDD, FRDC, FRDB, CYOA
Annotation Cluster 27	Enrichment Score: 1.9387457480329622	
Category	Term	Genes
SP_PIR_KEYWORDS	palmitate	MDTP, WZA, YDDW, YJBF, YEAY, APBE, SLYB, MLTB, MLTC, SPR, YFGH, CYOA
SP_PIR_KEYWORDS	lipoprotein	MDTP, DEDD, YAIW, APBE, YNBE, YCFL, WZA, YDDW, YJBF, YCAL, YEAY, SLYB, MLTB, LOLD, MLTC, SPR, LOLC, YFGH, CYOA, LOLE, YDEK
Annotation Cluster 28	Enrichment Score: 1.93564258224996	
Category	Term	Genes
GOTERM_BP_FAT	GO:0044265~cellular macromolecule catabolic process	RECJ, RNR, YGDG, RHLB, RND, VSR, MCRC, SBCB, EXOX
SP_PIR_KEYWORDS	nuclease	RECJ, RNR, YGDG, RND, VSR, POLA, SBCB, EXOX
GOTERM_BP_FAT	GO:0006308~DNA catabolic process	RECJ, YGDG, VSR, MCRC, SBCB, EXOX
SP_PIR_KEYWORDS	exonuclease	RECJ, RNR, YGDG, RND, POLA, SBCB, EXOX
GOTERM_MF_FAT	GO:0004518~nuclease activity	RECJ, RNR, VSR, POLA, MCRC, EXOX, YIHY, YGDG, YIHG, RND, CHO, MUTY, SBCB
Annotation Cluster 29	Enrichment Score: 1.9027039857776509	
Category	Term	Genes
SP_PIR_KEYWORDS	protein transport	TOLR, TOLQ, SAPF, SAPD, SAPB, SAPC, SECY
Annotation Cluster 30	Enrichment Score: 1.8528943960188873	
Category	Term	Genes
SP_PIR_KEYWORDS	sensory transduction	RCSA, NARQ, TORS, NARX, NARL
SP_PIR_KEYWORDS	nitrate assimilation	NARQ, NARX, NARL
Annotation Cluster 31	Enrichment Score: 1.6819744533635657	
Category	Term	Genes

GOTERM_BP_FAT	GO:0019674~NAD metabolic process	NADD, GMD, RFBB, CHBF, GLVG, PNCA
GOTERM_BP_FAT	GO:0006769~nicotinamide metabolic process	YGBJ, NADD, TPIA, GND, GMD, YIHU, RFBB, CHBF, GLVG, PNCA
GOTERM_BP_FAT	GO:0006732~coenzyme metabolic process	TPIA, NADD, FRDB, GND, GMD, YGGC, SCPC, YIHU, PABB, RFBB, CHBF, YGBJ, ACEE, MNGR, COAA, MQO, MODF, PANE, GLVG, PANF, PNCA
GOTERM_BP_FAT	GO:0009165~nucleotide biosynthetic process	NADD, GMD, CHBF, RFBB, PURU, PYRE, CYAA, GUAA, ZNTA, GLVG, PANE, ADD, PNCA
GOTERM_BP_FAT	GO:0051186~cofactor metabolic process	TPIA, NADD, FRDB, GND, GMD, YGGC, SCPC, PABB, YIHU, RFBB, MIND, CHBF, YGBJ, ACEE, MNGR, COAA, MQO, MODF, PANE, GLVG, PANF, PNCA
GOTERM_BP_FAT	GO:0006766~vitamin metabolic process	PDXH, NADD, RIBC, GMD, APBE, PABB, RFBB, CHBF, MIND, GLVG, PNCA

Annotation Cluster 32	Enrichment Score: 1.6706726762201645	
Category	Term	Genes
GOTERM_BP_FAT	GO:0044042~glucan metabolic process	MDOC, MDOB, MDOG, YEDQ, MDOH, BCSZ

Annotation Cluster 33	Enrichment Score: 1.6151514743006112	
Category	Term	Genes
GOTERM_BP_FAT	GO:0046364~monosaccharide biosynthetic process	TPIA, KDSA, NAGC, TDCG, FBP, RFBA, RFBC, AGAD
SP_PIR_KEYWORDS	gluconeogenesis	TPIA, TDCG, FBP
GOTERM_BP_FAT	GO:0019319~hexose biosynthetic process	TPIA, TDCG, FBP, RFBA, RFBC
GOTERM_BP_FAT	GO:0006090~pyruvate metabolic process	ACEE, TPIA, TDCG, FBP

Annotation Cluster 34	Enrichment Score: 1.5939361820273072	
Category	Term	Genes
SP_PIR_KEYWORDS	4fe-4s	FRDB, TDCG, YDHY, YNFE, YNFF, YDHX, HYFG, YNFG, YGFS, GLTD, RSXC, PFLA, YDEM, RSXB, MUTY, PFLC, YGFT
SP_PIR_KEYWORDS	iron	YHJA, FRDB, YBHJ, TDCG, YDHY, NAPB, YNFE, YNFF, YDHX, HYFG, YNFG, YCEJ, HCAC, YGFS, GLTD, PFLA, RSXC, YDEM, RSXB, MUTY, PFLC, CCME, YGGX, YGFT
SP_PIR_KEYWORDS	iron-sulfur	YBHJ, FRDB, TDCG, YDHY, YNFE, YNFF, YDHX, HYFG, YNFG, HCAC, YGFS, GLTD, PFLA, RSXC, YDEM, RSXB, MUTY, PFLC, YGFT
SP_PIR_KEYWORDS	iron-sulfur protein	FRDB, RSXC, RSXB, YNFE, YNFF, YNFG
INTERPRO	IPR017896:4Fe-4S ferredoxin, iron-sulpur binding domain	YDJY, FRDB, YGFS, GLTD, YDHY, RSXC, RSXB, PFLC, YDHX, YNFG, YGFT
INTERPRO	IPR001450:4Fe-4S ferredoxin, iron-sulphur binding, subgroup	FRDB, YGFS, YDHY, RSXC, RSXB, YDHX, YNFG, YGFT

GOTERM_MF_FAT	GO:0009055~electron carrier activity	FRDB, YDHY, YNFE, YNFF, YDHX, YNFG, YDHU, YDHV, GLTD, LLDD, YDIS, YHJA, AHPF, DSBB, HYFG, TORZ, GRXD, YCEJ, YCAK, HCAC, YGFS, TRXB, TRXC, YGCR, HCAD, RSXC, CYOA, RSXB, YDGJ, YGFT

Annotation Cluster 35	Enrichment Score: 1.5602878370273219	
Category	Term	Genes
KEGG_PATHWAY	ecd03070:Bacterial secretion system	GSPO, GSPJ, GSPK, GSPH, GSPI, SECY, FFH
SP_PIR_KEYWORDS	methylation	AER, PPDC, PPDB, GSPJ, GSPH, RPSK, GSPI
GOTERM_BP_FAT	GO:0045184~establishment of protein localization	TOLR, TOLQ, YHII, GSPJ, GSPK, GSPH, GSPI, SECY, SAPF, SAPD, SAPB, SAPC, LOLD, FFH
GOTERM_BP_FAT	GO:0015031~protein transport	TOLR, TOLQ, YHII, GSPJ, GSPK, GSPH, GSPI, SECY, SAPF, SAPD, SAPB, SAPC, LOLD, FFH
GOTERM_CC_FAT	GO:0015627~type II protein secretion system complex	GSPJ, GSPH, GSPI
GOTERM_BP_FAT	GO:0046903~secretion	YHII, GSPJ, GSPK, GSPH, GSPI

Annotation Cluster 36	Enrichment Score: 1.5347680896040683	
Category	Term	Genes
SP_PIR_KEYWORDS	ligase	ARGS, GUAA, MURF, RFAL, RIMK, GLYS, GLYQ, ASNS, DDLA, PABB, RTCA, SERS
KEGG_PATHWAY	ecj00970:Aminoacyl-tRNA biosynthesis	ARGS, GLYS, GLYQ, ASNS, SERS
GOTERM_BP_FAT	GO:0043039~tRNA aminoacylation	ARGS, PTH, GLYS, GLYQ, ASNS, SELD, TRUA, SERS
GOTERM_BP_FAT	GO:0043038~amino acid activation	ARGS, PTH, GLYS, GLYQ, ASNS, SELD, TRUA, SERS
GOTERM_BP_FAT	GO:0006418~tRNA aminoacylation for protein translation	ARGS, PTH, GLYS, GLYQ, ASNS, SELD, TRUA, SERS
KEGG_PATHWAY	ecc00970:Aminoacyl-tRNA biosynthesis	ARGS, GLYS, GLYQ, SERS
GOTERM_BP_FAT	GO:0006399~tRNA metabolic process	ARGS, YEDF, YIHY, PTH, GLYS, RND, GLYQ, ASNS, CMOA, SELD, TRUA, SERS
GOTERM_BP_FAT	**GO:0034660~ncRNA metabolic process**	ASNS, SERS, RLML, YEDF, ARGS, YIHY, RLUE, PTH, GLYS, RND, GLYQ, SELD, CMOA, TRUA

Annotation Cluster 37	Enrichment Score: 1.524800314407734	
Category	Term	Genes
GOTERM_MF_FAT	GO:0031420~alkali metal ion binding	TRKG, PYKF, YIDK, MDTK, KEFA, PYKA, PANF, KEFB
SP_PIR_KEYWORDS	potassium	TRKG, PYKF, KEFA, PYKA, KEFB

GOTERM_BP_FAT	GO:0006813~potassium ion transport	TRKG, YBAL, KEFA, KEFB

Annotation Cluster 38	Enrichment Score: 1.4844722414214753	
Category	Term	Genes
KEGG_PATHWAY	eco00640:Propanoate metabolism	TDCE, TDCD, SCPA, SCPB, FADJ, PAAG

Annotation Cluster 39	Enrichment Score: 1.4707755334398063	
Category	Term	Genes
SP_PIR_KEYWORDS	dna repair	HELD, MUTS, LEXA, VSR, RECQ, CHO, POLA, MODF, MUTY, SBCB, EXOX
SP_PIR_KEYWORDS	DNA damage	MUTS, LEXA, VSR, RECQ, CHO, POLA, MODF, MUTY, SBCB, EXOX
GOTERM_BP_FAT	GO:0033554~cellular response to stress	RFAG, RECJ, LEXA, VSR, POLA, EXOX, HELD, MUTS, RECQ, CHO, TRXB, MODF, IRAP, DINF, MUTY, SBCB
GOTERM_BP_FAT	GO:0006974~response to DNA damage stimulus	RECJ, LEXA, VSR, POLA, EXOX, HELD, MUTS, RECQ, CHO, MODF, DINF, MUTY, SBCB
GOTERM_BP_FAT	GO:0006259~DNA metabolic process	MCRC, HIPA, HIPB, EXOX, DNAT, HOLA, RECQ, CHO, DINF, RECJ, YFDO, INSG, LEXA, VSR, POLA, HOLE, YFDM, HELD, TDK, YGDG, MUTS, IHFA, REND, MODF, MUTY, SBCB

Annotation Cluster 40	Enrichment Score: 1.4567075871324318	
Category	Term	Genes
SP_PIR_KEYWORDS	lipid metabolism	HDHA, FADR, FADJ, PAAG

Annotation Cluster 41	Enrichment Score: 1.36116679833629	
Category	Term	Genes
SP_PIR_KEYWORDS	Flavoprotein	PDXH, YDIS, AER, GLPA, YGCU, AHPF, YCAK, LLDD, TRXB, YGCR, HCAD, MQO
SP_PIR_KEYWORDS	FAD	YDIS, AER, GLPA, YGCU, AHPF, TRXB, YGCR, HCAD, MQO
INTERPRO	IPR001327:Pyridine nucleotide-disulphide oxidoreductase, NAD-binding region	AHPF, GLTD, TRXB, HCAD, YGFT
INTERPRO	IPR013027:FAD-dependent pyridine nucleotide-disulphide oxidoreductase	YDIS, AHPF, GLTD, TRXB, HCAD, YGFT

Annotation Cluster 42	Enrichment Score: 1.3514776022801531	
Category	Term	Genes
GOTERM_MF_FAT	GO:0031420~alkali metal ion binding	TRKG, PYKF, YIDK, MDTK, KEFA, PYKA, PANF, KEFB

SP_PIR_KEYWORDS	Sodium	YIDK, MDTK, PANF
SP_PIR_KEYWORDS	Sodium transport	YIDK, MDTK, PANF

Annotation Cluster 43	Enrichment Score: 1.3347730476755864	
Category	Term	Genes
KEGG_PATHWAY	ecf03410:Base excision repair	RECJ, POLA, MUTY
