# Supplementary material for: Central role for RNase YbeY in Hfq-dependent and Hfq-independent small-RNA regulation in bacteria
Source: BMC Genomics. 2014 Feb 11;15:121. doi: 10.1186/1471-2164-15-121 (PMC3933206; doi:10.1186/1471-2164-15-121)
Supplement: Additional file 9: Table S9 — Functional clusters of genes differentially regulated upon HU exposure only in YbeY presence (Figure 2). [file 1471-2164-15-121-S9.pdf]

Supplemental Table 9. Functional clusters of genes differentially regulated upon HU exposure only in YbeY presence (Figure 2)

Annotation Cluster 1	Enrichment Score: 27.091408410016633	
Category	Term	Genes
SP_PIR_KEYWORDS	membrane	GUDP, LGT, AAS, HTPG, MALF, MALG, YCJF, HTPX, TRKH, MALK, YDJK, ABRB, YIAV, NIKD, NIKB, YDJE, NIKC, ACRE, PUUP, YBBY, GNTP, BCR, HYBC, SYD, HYBB, CAIT, HYBO, RHTC, YICE, YBCI, APPB, DLD, HTRE, RFBX, GFCE, SFMD, GSIA, NAGE, YCHE, LAMB, RCSD, RCSF, YCHM, YHAH, YIAD, AFUB, PLSB, CYNX, YAAU, YAAJ, HSRA, YQEG, TTDT, YAFT, SSUB, NARH, NARG, MDTE, MDTF, NARK, YIGM, NLPE, GSPL, GSPM, FEOB, HEMX, CLD, YPDA, GFCA, EVGS, CITT, SUGE, YBGQ, YHBX, YBGT, YQGA, YIDH, YIDI, FTSI, YJJP, ENVZ, YADH, FTSQ, FTSW, LPXL, YADS, YJIY, MACA, YIDC, PQIA, MNGA, SHIA, WZXC, OSMB, NANC, YHID, ZITB, HDED, PSTC, YCBB, PSTB, NUOG, MRCB, NANT, YPHA, YQHA, YFHM, YFHK, HOFC, YJET, RFC, NUOH, NUOI, NUOJ, UBIB, YEDD, NUPG, YCCM, MRDA, FDOI, YDCU, YBJP, YBJO, FDOH, PGSA, YDCO, YAJG, YAJI, CUSA, CADC, YEGT, LPTB, CADB, YFGO, YTFB, WECH, PPX, YQJA, NAPH, YAIY, YFGM, KDPD, TATA, WAAU, TATE, YFGA, UHPB, GLNQ, PSUT, NMPC, YJIJ, YQJE, YPFN, BTUB, TORC, TAUC, EMRD, TAUB, TAUD, FLK, YHFL, CCMG, CCMH, YAHC, CCMF, FHUC, YGIH, YEAI, YKGB, DCUA, YFEO, NRFD, YJCE, SECA, YFDY, FHUB, FHUA, YNEF, MDLA, GLPC, ZUPT, RSEC, MURG, RBN, PYRD, YPJA, PGAB, BCSA, PGAD, IDNT, YEBZ, SDAC, YJEP, FADL, YJEM, YRBK, YEBO, TONB, HFLB, HFLD, YJDF, BAMD, ZRAS, CYST, BAMB, HFLK, YEBE, YDAN

Annotation Cluster 2	Enrichment Score: 16.347572556990297	
Category	Term	Genes
SP_PIR_KEYWORDS	iron	FHUC, FHUD, HYCB, DEF, FIXX, FES, NUOG, NRFB, MHPB, NRFC, NRFF, BFR, NARH, HEMH, NARG, HEMN, FHUB, FHUA, ENTF, HYBC, HYBA, HYBB, HYBO, FEOB, GLPC, YSAA, NUOI, RUMA, YCCM, APPB, FDOI, YGCF, DOS, FDOG, FDOH, YGCO, YJJW, NAPH, NFUA, NAPF, EFEB, ISPG, NAPA, DPS, MOAA, YJES, SDAB, SDAA, AEGA, HCR, HCP, NRFA, TORC, GARL, TAUD, CYBC, YBIY, CCMH
GOTERM_MF_FAT	GO:0046914~transition metal ion binding	HYCI, HYCB, FIXX, ZITB, SURE, EUTB, NUOG, HTPX, MHPB, YDJL, NIKD, NANK, NIKB, ZUR, NIKC, NIKA, ENTF, TOPA, PDXK, HYBC, HYBA, GLTX, HYBB, HYBO, YSAA, NUOI, NIKR, PDXY, APPB, YCCM, FDOI, RIBD, YGCF, RIBA, FDOG, FDOH, YGCO, CUSA, NAPH, NFUA, NAPF, FBAA, ISPG, NAPA, YBDH, NRDR, DNAQ, MOAA, AEGA, YFGC, NRFA, TORC, GARL, GUAD, TAUD, GLMU, PHP, YBEY, PRIA, FOLE, CCMH, FHUC, FHUD, DEF, FES, NRFB, METH, HEMB, SECA, NRFC, BFR, NRFF, NARH, HEMH, DXS, NARG, GLPX, FHUB, HEMN, FHUA, NLPE, FEOB, GLPC, PURD, RUMA, ZUPT, RBN, DOS, ADE, HSLO, PEPQ, YJJW, USHA, RPPH, EFEB, CUEO, AMPD, BISC, DPS, YJJG, YFCE, DCP, SDAB, YJES, SDAA, RPOH, HCR, HCP, DDLB, MAP, RSGA, CYBC, YBIY, HFLB, CYSS, ZRAS, YDAN, YFAU, SODC

GOTERM_MF_FAT	GO:0043169~cation binding	HYCI, UPP, HYCB, FIXX, ZITB, SURE, EUTB, NUOG, HTPX, TRKH, MHPB, GUDD, YDJL, NIKD, NANK, ZUR, NIKB, NIKC, NIKA, ENTF, TOPA, PDXK, HYBC, HYBA, GLTX, HYBB, HYBO, KDSB, YSAA, NUOI, NIKR, PDXY, APPB, YCCM, FDOI, RIBD, YGCF, RIBA, FDOG, FDOH, YGCO, CUSA, PURT, PPX, YEGX, NAPH, NFUA, NAPF, ISPG, FBAA, YBDH, NAPA, NRDR, TATD, DNAQ, MOAA, RUVC, AEGA, YFGC, BTUB, NRFA, TORC, GARL, PTSP, GUAD, TAUD, GLMU, GUAB, PHP, YBEY, PRIA, FOLE, CCMH, DUT, FHUC, FHUD, DEF, CPDB, PRS, FES, YJCE, NRFB, METH, HEMB, SECA, NRFC, BFR, NRFF, HEMH, NARH, DINB, NARG, DXS, GLPX, HEMN, FHUB, FHUA, GLGB, PURF, NLPE, YBGI, FEOB, GLPC, PURD, RUMA, ZUPT, RBN, DOS, ADE, PEPQ, HSLO, YJJW, USHA, RPPH, EFEB, CUEO, AMPD, FRYA, BISC, DPS, YJJG, YFCE, DCP, UIDA, YJES, SDAB, SDAA, RPOH, HCR, MENC, HCP, DDLB, HPT, GCL, MAP, RSGA, CYBC, YBIY, HFLB, CYSS, ZRAS, YBIV, YDAN, YFAU, SODC

Annotation Cluster 3	Enrichment Score: 13.892992058293736	
Category	Term	Genes
SP_PIR_KEYWORDS	signal	NANC, YIBG, MALG, MALE, HDEB, HDEA, ALSB, MALM, SPY, MATC, YIAV, NANM, BGLX, NIKC, ACRE, NIKA, YFHM, HYBA, HYBO, YEDD, HTRE, EUTM, FDOG, YBJP, YAJG, YAJI, YFHG, DCRB, GFCE, CADB, LPTA, SFMD, GSIB, YTFJ, NAPA, PBPG, LAMB, RCSF, YQJC, YFGC, BTUB, NRFA, EMRD, YIAD, DEGP, YJHS, ENDA, CYNX, TORT, YTFM, YAAI, CCMH, FHUD, CPDB, PROX, YAFT, TESA, NRFB, NRFC, CSGC, YMGD, NRFF, MDTE, FHUA, NLPE, YAGV, GFCA, RSEB, EVGS, LOLA, YBGQ, AMIA, YAGX, USHA, ECPD, YBHG, YRAH, YPJA, EFEB, PGAB, CUEO, ENVZ, YADE, YRAP, YADN, YADM, YJEI, YADL, YJJA, YJEP, FADL, MACA, YBAV, DSBA, CYBC, HFLB, BAMD, BAMB, OSMB, SODC

Annotation Cluster 4	Enrichment Score: 13.029446537638417	
Category	Term	Genes
GOTERM_BP_FAT	GO:0009061~anaerobic respiration	YJJW, HYCB, NAPH, NAPF, NAPD, NAPA, NRFD, NUOG, NRFB, NRFC, NRFF, NRFG, NARH, NARG, NIKD, NARJ, GLTA, HYBG, MENB, MENC, NRFA, MENE, HYBC, TORC, HYBB, HYBO, GLPC, NUOH, NUOI, NUOJ, TORR, YBIY, FDOI, TORT, NIRD, FDOG, FDOH
GOTERM_BP_FAT	GO:0006091~generation of precursor metabolites and energy	HYCB, FIXX, NRFD, NUOG, NRFB, NRFC, NRFF, NRFG, NARH, NARG, NIKD, NARJ, HYCA, GLTA, PGK, GLGB, HYBG, PDXK, HYBC, HYBB, HYBO, GLPC, NUOH, YSAA, NUOI, GLGP, NUOJ, PDXY, YCCM, APPB, FDOI, TRXA, DLD, FDOG, FDOH, YGCO, PFKB, YJJW, NAPH, NAPF, NAPD, FBAA, NAPA, YTJC, MENB, MENC, HCR, MENE, NRFA, TORC, MIOC, TORR, GPSA, CYBC, YBIY, FIXC, TORT, FIXB, NIRD, ZRAS, FLDA, ZRAR, GLK, RHO

Annotation Cluster 5	Enrichment Score: 9.932385112588221	
Category	Term	Genes
KEGG_PATHWAY	eco00230:Purine metabolism	PURH, PURT, USHA, PURF, PPX, PURL, CPDB, PRS, HPT, GUAD, PURD, SURE, PURC, YBCF, PURB, YJJG, DNAQ, GUAB, HOLD, HOLB, ADE, RPOZ, GMK, ALLD

Annotation Cluster 6	Enrichment Score: 8.21951015136021	
Category	Term	Genes
SP_PIR_KEYWORDS	metalloprotein	NRFA, HYCB, HYBC, TORC, HYBO, NUOI, MAP, NRFB, NRFC, YCCM, YBIY, NARH, NARG, FDOG, FDOH, SODC
SP_PIR_KEYWORDS	electron transfer	NRFB, NRFC, NRFA, YCCM, TORC, NARH, FLDA, NARG, FDOH, NUOI
SP_PIR_KEYWORDS	iron-sulfur protein	NRFC, YCCM, HYCB, NARH, NARG, FDOG, FDOH, NUOI

Annotation Cluster 7	Enrichment Score: 7.942358472198253	
Category	Term	Genes
SP_PIR_KEYWORDS	electron transport	HYCB, FIXX, NAPH, NAPF, NAPA, NRFB, NRFC, NARH, NARG, HCR, NRFA, TORC, HYBB, GLPC, MIOC, YSAA, APPB, CYBC, YCCM, FDOI, FIXC, TRXA, FIXB, FLDA, FDOH, YGCO
GOTERM_BP_FAT	GO:0006091~generation of precursor metabolites and energy	HYCB, FIXX, NRFD, NUOG, NRFB, NRFC, NRFF, NRFG, NARH, NARG, NIKD, NARJ, HYCA, GLTA, PGK, GLGB, HYBG, PDXK, HYBC, HYBB, HYBO, GLPC, NUOH, YSAA, NUOI, GLGP, NUOJ, PDXY, YCCM, APPB, FDOI, TRXA, DLD, FDOG, FDOH, YGCO, PFKB, YJJW, NAPH, NAPF, NAPD, FBAA, NAPA, YTJC, MENB, MENC, HCR, MENE, NRFA, TORC, MIOC, TORR, GPSA, CYBC, YBIY, FIXC, TORT, FIXB, NIRD, ZRAS, FLDA, ZRAR, GLK, RHO
GOTERM_BP_FAT	GO:0022900~electron transport chain	HYCB, FIXX, NAPH, NAPF, NAPA, NUOG, NRFB, NRFC, NARH, NARG, HCR, NRFA, TORC, HYBB, GLPC, MIOC, YSAA, APPB, CYBC, YCCM, FDOI, FIXC, TRXA, FIXB, FLDA, FDOH, YGCO
GOTERM_MF_FAT	GO:0009055~electron carrier activity	YJJW, HYCB, NAPH, NAPF, NAPA, BISC, NUOG, NRFC, GOR, HEMG, YJES, NARG, AEGA, YKGG, YJHC, HCR, HCP, HYBA, TORC, GLPC, NUOI, YSAA, TAUD, YCCM, CYBC, FDOI, TRXA, FIXB, NIRD, FLDA, FDOG, FDOH, YGCO

Annotation Cluster 8	Enrichment Score: 7.671568463193634	
Category	Term	Genes
KEGG_PATHWAY	eco00670:One carbon pool by folate	PURH, PURT, FOLA, METH, FOLM, GCVT, FMT, GLYA, THYA, FOLD

Annotation Cluster 9	Enrichment Score: 6.980994383574671	
Category	Term	Genes
SP_PIR_KEYWORDS	phosphoprotein	CITD, HTPG, KDPD, NAGE, ENVZ, YTJC, FRYA, UHPB, DPIA, RCSD, CREB, EVGA, CYSK, ENTF, YFHK, ALR, ARCA, GLYA, CAIC, GLGP, TORR, GCVP, YFHA, EVGS, BAER, ZRAS, FDOG, ZRAR, OMPR, BASR
KEGG_PATHWAY	ecd02020:Two-component system	CITD, KDPD, ENVZ, UHPB, DPIA, NARH, NARG, NARJ, RCSD, RCSF, CREB, EVGA, YFHK, TORC, ARCA, TORR, DEGP, YFHA, CITT, BAER, ZRAS, ZRAR, OMPR, BASR, CUSA
INTERPRO	IPR001789:Signal transduction response regulator, receiver region	TORR, EVGA, DPIA, YFHA, EVGS, BAER, ZRAR, ARCA, OMPR, CREB, BASR
UP_SEQ_FEATURE	domain:Response regulatory	TORR, EVGA, DPIA, YFHA, EVGS, BAER, ZRAR, ARCA, OMPR, CREB, BASR

Annotation Cluster 10	Enrichment Score: 6.499500185419999	
Category	Term	Genes
SP_PIR_KEYWORDS	nucleotide-binding	ASPS, AAS, HTPG, SURE, PSTB, YOAA, MALK, NIKD, RARA, NANK, PGK, ENTF, TMK, YFHK, TOPA, HOFB, TOPB, PDXK, GLTX, MNMA, THIK, YBCF, RECF, PDXY, YFHA, YDCT, RIBA, EUTP, PURT, LPTB, DNAC, GSIA, KDPD, GYRB, NRDR, YEGD, DHAR, UHPB, MOAA, GLNQ, GLNS, RCSD, DGOK, GMK, GARK, GLNE, COAE, TAUB, PRPR, ISPE, DBPA, PHR, PCNB, COAD, PRIA, FOLE, GLK, RHO, FHUC, CPDB, PRS, PRPE, METL, SECA, HDA, SSUB, GLXK, YIHA, YKFA, PURL, MDLA, FEOB, PURD, PROB, PURC, YGHT, EVGS, HSDR, GSHA, PFKB, USHA, HISS, YBHF, ENVZ, YJJG, RFAY, FTSZ, SUFC, MENE, MAK, DDLB, IDNK, RSGA, HFLB, CYSS, ZRAS, ZRAR
SP_PIR_KEYWORDS	atp-binding	FHUC, ASPS, AAS, PRS, HTPG, PRPE, METL, PSTB, YOAA, SECA, MALK, SSUB, NIKD, RARA, NANK, GLXK, PGK, TMK, ENTF, TOPA, YFHK, HOFB, PDXK, TOPB, PURL, GLTX, THIK, MNMA, MDLA, PROB, PURD, PURC, YBCF, RECF, PDXY, YGHT, YFHA, EVGS, YDCT, GSHA, HSDR, EUTP, PFKB, PURT, LPTB, GSIA, DNAC, HISS, YPJA, KDPD, YBHF, GYRB, ENVZ, NRDR, YEGD, DHAR, UHPB, GLNQ, RFAY, GLNS, RCSD, GMK, DGOK, GARK, GLNE, SUFC, MENE, COAE, MAK, DDLB, TAUB, IDNK, PRPR, ISPE, HFLB, DBPA, CYSS, PCNB, ZRAS, ZRAR, COAD, PRIA, GLK, RHO

Annotation Cluster 11	Enrichment Score: 5.90301100354393	
Category	Term	Genes
GOTERM_BP_FAT	GO:0016051~carbohydrate biosynthetic process	NANE, FTSI, BCSA, NAGE, YTJC, NANA, WAAU, FTSW, MRCB, LPXL, NANT, RFAZ, NAGB, RFAY, NAGA, PBPG, SDAB, SDAA, GALE, NANK, LPXH, RCSF, PGK, GLGB, RFAI, RFAJ, DDLB, WCAC, ALR, KDSB, CLD, RFC, WZXC, GLGP, GPSA, GLMU, MRDA, MURG, MURI, AGAW, GLF, AMIA, RFBX, EPD

Annotation Cluster 12	Enrichment Score: 5.792713598893311	
Category	Term	Genes
KEGG_PATHWAY	ecd00240:Pyrimidine metabolism	USHA, TMK, UPP, CPDB, SURE, YJJG, PYRF, DNAQ, HOLD, HOLB, PYRD, RPOZ, THYA, DUT

Annotation Cluster 13	Enrichment Score: 5.24850957400413	
Category	Term	Genes
KEGG_PATHWAY	eco00860:Porphyrin and chlorophyll metabolism	HEMB, HEMC, HEMD, CYSG, GLTX, UIDA, HEMG, HEMH, HEMX, HEMN

Annotation Cluster 14	Enrichment Score: 5.150728776157705	
Category	Term	Genes
SP_PIR_KEYWORDS	heme	NRFB, APPB, NRFA, CYBC, FDOI, NRFF, BFR, TORC, HYBB, EFEB, DOS, CCMH

GOTERM_BP_FAT	GO:0043933~macromolecular complex subunit organization	RPOH, NRFA, TORC, NUSB, HYBB, RPON, PRFA, SECB, NRFB, CYBC, FDOI, NRFF, RPOS, NRFG, FTSZ, CCMG, CCMH, RHO, CCMF
GOTERM_BP_FAT	GO:0070271~protein complex biogenesis	RPOH, NRFA, TORC, HYBB, RPON, NRFB, SECB, CYBC, FDOI, NRFF, RPOS, FTSZ, NRFG, CCMG, CCMH, CCMF
GOTERM_BP_FAT	GO:0017004~cytochrome complex assembly	NRFB, NRFA, CYBC, FDOI, NRFF, TORC, HYBB, NRFG, CCMG, CCMH, CCMF
Annotation Cluster 15	Enrichment Score: 4.665546389601961	
Category	Term	Genes
SP_PIR_KEYWORDS	magnesium	PURT, PPX, RPPH, UPP, PRS, TATD, SURE, FRYA, YJJG, HEMB, DNAQ, GUDD, DXS, DINB, RUVC, GMK, MENC, PURF, PDXK, GARL, DDLB, HPT, PTSP, GCL, KDSB, PURD, GLMU, DOS, RIBA, YBIV, YFAU, DUT
Annotation Cluster 16	Enrichment Score: 4.484340146938689	
Category	Term	Genes
SP_PIR_KEYWORDS	iron	FHUC, FHUD, HYCB, DEF, FIXX, FES, NUOG, NRFB, MHPB, NRFC, NRFF, BFR, NARH, HEMH, NARG, HEMN, FHUB, FHUA, ENTF, HYBC, HYBA, HYBB, HYBO, FEOB, GLPC, YSAA, NUOI, RUMA, YCCM, APPB, FDOI, YGCF, DOS, FDOG, FDOH, YGCO, YJJW, NAPH, NFUA, NAPF, EFEB, ISPG, NAPA, DPS, MOAA, YJES, SDAB, SDAA, AEGA, HCR, HCP, NRFA, TORC, GARL, TAUD, CYBC, YBIY, CCMH
SP_PIR_KEYWORDS	4fe-4s	YJJW, HYCB, FIXX, NAPH, NFUA, NAPF, ISPG, NAPA, NUOG, NRFC, MOAA, NARH, SDAB, YJES, NARG, SDAA, AEGA, HEMN, HYBA, HYBO, GLPC, NUOI, YSAA, RUMA, YCCM, YBIY, YGCF, FDOG, FDOH
Annotation Cluster 17	Enrichment Score: 3.9173645522070517	
Category	Term	Genes
SP_PIR_KEYWORDS	ATP	FHUC, LPTB, ASPS, GSIA, HISS, GYRB, BISC, PSTB, HTPX, SECA, MALK, GLNQ, SSUB, GLNS, NIKD, MDTF, PGK, GMK, TMK, SUFC, HOFB, GLTX, MDLA, CAIC, DBPA, CYSS, HSDR, PRIA
KEGG_PATHWAY	eco02010:ABC transporters	FHUC, LPTB, FHUD, GSIB, GSIA, TAUC, MALF, TAUB, MALG, AFUB, MALE, PSTC, YADH, PSTB, PROX, ALSB, MALK, GLNQ, SSUB, NIKD, NIKB, NIKC, FHUB, NIKA
GOTERM_MF_FAT	GO:0042625~ATPase activity, coupled to transmembrane movement of ions	NIKD, NIKB, NIKC, NIKA, PSTB
INTERPRO	IPR017871:ABC transporter, conserved site	FHUC, LPTB, SUFC, GSIA, MDLA, TAUB, YBHF, PSTB, MALK, SSUB, GLNQ, YDCT, NIKD
INTERPRO	IPR003593:ATPase, AAA+ type, core	FHUC, LPTB, HOFB, SUFC, GSIA, DNAC, MDLA, YBHF, TAUB, PSTB, PRPR, DHAR, MALK, SSUB, HFLB, GLNQ, YFHA, YDCT, RARA, NIKD, ZRAR, RHO

GOTERM_MF_FAT	GO:0016887~ATPase activity	FHUC, LPTB, SUFC, GSIA, MDLA, YBHF, TAUB, YADH, PSTB, YOAA, MALK, SSUB, HFLB, GLNQ, DBPA, YDCT, NIKD, NIKB, PRIA, NIKC, NIKA
Annotation Cluster 18	Enrichment Score: 3.868061177406305	
Category	Term	Genes
KEGG_PATHWAY	ecv00230:Purine metabolism	PURH, PURT, USHA, PURF, PPX, PURL, CPDB, HPT, PURD, SURE, PURC, PURB, YJJG, DNAQ, GUAB, HOLB, ADE, RPOZ, GMK, ALLD
Annotation Cluster 19	Enrichment Score: 3.8210636025927815	
Category	Term	Genes
SP_PIR_KEYWORDS	carbon-oxygen lyase	CYSK, CAIB, HEMB, HEMD, GUDD, CYNS, SDAB, AROD, SDAA
SP_PIR_KEYWORDS	hydro-lyase	CAIB, HEMB, HEMD, GUDD, CYNS, SDAB, AROD, SDAA
Annotation Cluster 20	Enrichment Score: 3.819127034274014	
Category	Term	Genes
GOTERM_BP_FAT	GO:0044271~nitrogen compound biosynthetic process	UPP, YGIF, PRS, MOG, METL, PROX, HEMB, METH, HEMC, HEMD, HEMG, HEMH, DXS, HEMN, PURH, TMK, PURF, PURL, PABC, ALR, GLYA, HEMX, RHTC, PROB, QUEC, PURD, PURC, YBCF, PURB, PROC, PYRF, RIBD, AROG, RIBE, PYRD, AROD, RIBA, THYA, PURT, CADB, ASD, ILVL, NAPA, ILVC, BISC, SPEC, MOAD, TYRB, MOAA, GLNQ, SDAC, GALE, AEGA, GMK, CYSK, NADC, IVBL, CYSG, HPT, WRBA, DAPF, FOLA, GUAB, ILVY, MTN, FOLE, FOLD, RHO, HISL
SP_PIR_KEYWORDS	amino-acid biosynthesis	CYSK, IVBL, ASD, ILVL, PROB, DAPF, ILVC, METL, PROC, METH, TYRB, AROG, AROD, MTN, ILVY, FOLD, HISL
Annotation Cluster 21	Enrichment Score: 3.644975593106647	
Category	Term	Genes
GOTERM_BP_FAT	GO:0006766~vitamin metabolic process	FIXX, BISC, DXS, GALE, MENB, MENC, CAIB, NADC, PDXK, MENE, CYSG, PABC, DKGB, GLYA, CAIE, PDXY, SELA, FOLA, FIXC, RIBD, FIXB, RIBE, RIBA, FOLE, FOLD, EPD
Annotation Cluster 22	Enrichment Score: 3.6307365196164296	
Category	Term	Genes
KEGG_PATHWAY	eck00910:Nitrogen metabolism	NRFA, NIRD, TNAA, CYNS, NARH, NARG, GCVT, NAPA, NARJ, YBCF
GOTERM_MF_FAT	GO:0016661~oxidoreductase activity, acting on other nitrogenous compounds as donors	NRFB, NRFC, NRFA, HCP, NAPH, NIRD, NARH, NAPD, NARG, NAPA, NARJ

Annotation Cluster 23	Enrichment Score: 3.483537224371926	
Category	Term	Genes
KEGG_PATHWAY	eum00260:Glycine, serine and threonine metabolism	GARK, ASD, GCVP, SDAB, GCVT, SDAA, GLYA, GLXK, METL

Annotation Cluster 24	Enrichment Score: 3.1512856893410395	
Category	Term	Genes
SP_PIR_KEYWORDS	phosphate transport	PSTC, PHOU, PSTB
INTERPRO	IPR005670:Phosphate transport system permease protein 1	PSTB
INTERPRO	IPR015850:ABC transporter, phosphate import, PstB	PSTB
GOTERM_MF_FAT	GO:0015415~phosphate transmembrane-transporting ATPase activity	PSTB
GOTERM_BP_FAT	GO:0015698~inorganic anion transport	CYST, SSEA, PSTC, PHOU, NARK, YCHM, YFEO, PSTB

Annotation Cluster 25	Enrichment Score: 3.1181401056680267	
Category	Term	Genes
GOTERM_BP_FAT	GO:0046349~amino sugar biosynthetic process	MRCB, NANE, NANT, NAGB, MURG, NAGA, AGAW, NAGE, NANK, NANA
KEGG_PATHWAY	ecj00520:Amino sugar and nucleotide sugar metabolism	NANE, GLMU, NAGB, MAK, NAGA, GALE, NAGE, NANK, NANA, YAGH, GLK
SP_PIR_KEYWORDS	carbohydrate metabolism	NANE, RHAM, NAGB, MAK, NAGA, NANM, GALE, GALR, NANK, NANA, GLPX, GLGP

Annotation Cluster 26	Enrichment Score: 2.958892606400057	
Category	Term	Genes
KEGG_PATHWAY	ecj00270:Cysteine and methionine metabolism	CYSK, METH, ASD, TYRB, SDAB, SSEA, SDAA, MTN, METL
GOTERM_BP_FAT	GO:0006790~sulfur metabolic process	CYSK, ASD, TAUC, TAUB, SSEA, YBBB, TAUD, METL, BISC, SUFS, METH, MOAD, GOR, SSUB, SSUD, GSHA, DXS, MTN, FOLD

Annotation Cluster 27	Enrichment Score: 2.852257349266379	
Category	Term	Genes
GOTERM_BP_FAT	GO:0006631~fatty acid metabolic process	FABD, FABF, PRPE, PRPB, MHPC, PRPR, FABG, MHPA, FABH, MHPB, ACCB, ACPH, SDAB, FADL

Annotation Cluster 28	Enrichment Score: 2.756736845637827	
Category	Term	Genes
SP_PIR_KEYWORDS	palmitate	GFCE, YFHM, NLPE, YIAD, PGAB, YAFT, YEDD, BAMD, BAMB, MDTE, YBJP, OSMB, YAJG, YAJI, ACRE
GOTERM_CC_FAT	GO:0031225~anchored to membrane	YFHM, NLPE, YIAD, PGAB, YAFT, YEDD, BAMD, BAMB, MDTE, YBJP, OSMB, YAJG, YAJI, ACRE, YPFN
SP_PIR_KEYWORDS	lipoprotein	GFCE, YFHM, NLPE, YIAD, PGAB, YADE, YAFT, YEDD, BAMD, LOLA, BAMB, MDTE, YBJP, OSMB, YAJG, YAJI, ACRE

Annotation Cluster 29	Enrichment Score: 2.722013632090176	
Category	Term	Genes
INTERPRO	IPR002509:Polysaccharide deacetylase	PGAB, YADE

Annotation Cluster 30	Enrichment Score: 2.561717609579521	
Category	Term	Genes
KEGG_PATHWAY	ecg00564:Glycerophospholipid metabolism	GPSA, AAS, GLPC, PGSA, EUTB, PLSB

Annotation Cluster 31	Enrichment Score: 2.5547766226192237	
Category	Term	Genes
KEGG_PATHWAY	ecq00630:Glyoxylate and dicarboxylate metabolism	GARK, FDOI, GARR, GCL, FDOG, FDOH, GLXK, GLTA, FOLD
KEGG_PATHWAY	ecv00680:Methane metabolism	FDOI, FDOG, FDOH, GLYA
SP_PIR_KEYWORDS	heterotrimer	FDOI, FDOG, FDOH
GOTERM_MF_FAT	GO:0016620~oxidoreductase activity, acting on the aldehyde or oxo group of donors, NAD or NADP as acceptor	ASD, FDOI, FDOG, FDOH, YSAA, EPD

Annotation Cluster 32	Enrichment Score: 2.5404935542735196	
Category	Term	Genes
GOTERM_BP_FAT	GO:0009451~RNA modification	YBEA, RSMF, MNMA, LIGT, MNMG, QUEC, DTD, RRMJ, RUMA, SELA, RLUC, DBPA, RLUD, RLUA, PCNB, RLUB, TRUD, RLMF, FMT, RSUA
SP_PIR_KEYWORDS	rrna processing	RUMA, YBEA, RSMF, RLUC, RLUD, RLUA, RLUB, RLMF, RSUA, GIDB, RRMJ
GOTERM_BP_FAT	GO:0022613~ribonucleoprotein complex biogenesis	YBEA, RSMF, RRMJ, RUMA, RMF, RNC, RLUC, RLUD, DBPA, RLUA, RLUB, RLMF, RSUA, GIDB

GOTERM_BP_FAT	GO:0042254~ribosome biogenesis	YBEA, RSMF, RRMJ, RUMA, RMF, RNC, RLUC, RLUD, DBPA, RLUA, RLUB, RLMF, RSUA, GIDB
GOTERM_BP_FAT	GO:0034470~ncRNA processing	YBEA, RSMF, MNMA, MNMG, DUSC, QUEC, RRMJ, RUMA, RBN, RLUC, RNC, RLUD, KPTA, RLUA, RLUB, TRUD, RLMF, RSUA, GIDB, RPH
GOTERM_BP_FAT	GO:0006399~tRNA metabolic process	ASPS, GLTX, HISS, MNMA, MNMG, DUSC, QUEC, RBN, GLNS, KPTA, RLUA, CYSS, TRUD, RPH
GOTERM_BP_FAT	GO:0008033~tRNA processing	RBN, KPTA, MNMA, RLUA, TRUD, MNMG, DUSC, QUEC, RPH
GOTERM_BP_FAT	GO:0006400~tRNA modification	MNMA, TRUD, MNMG, QUEC
Annotation Cluster 33	Enrichment Score: 2.5101008116264243	
Category	Term	Genes
KEGG_PATHWAY	ecg00230:Purine metabolism	PURH, PURT, USHA, PURF, PPX, PURL, CPDB, PRS, HPT, GUAD, PURD, SURE, PURC, YBCF, PURB, YJJG, DNAQ, GUAB, HOLD, HOLB, RPOZ, GMK, ALLD
Annotation Cluster 34	Enrichment Score: 2.4682438929724873	
Category	Term	Genes
KEGG_PATHWAY	ecj00500:Starch and sucrose metabolism	GLGB, MAK, UIDA, BCSA, YAGH, BGLX, GLK, GLGP
Annotation Cluster 35	Enrichment Score: 2.400942183093628	
Category	Term	Genes
GOTERM_BP_FAT	GO:0009437~carnitine metabolic process	CAIB, FIXC, FIXX, FIXB, CAIE
GOTERM_BP_FAT	GO:0006575~cellular amino acid derivative metabolic process	CAIB, FIXX, CAIE, WRBA, PROX, SPEC, METH, GOR, FIXC, FIXB, TNAA, GSHA, MTN, EUTP
GOTERM_BP_FAT	GO:0006576~biogenic amine metabolic process	PROX, SPEC, CAIB, FIXC, FIXX, FIXB, TNAA, EUTP, WRBA, CAIE
Annotation Cluster 36	Enrichment Score: 2.36996752030282	
Category	Term	Genes
SP_PIR_KEYWORDS	protein biosynthesis	RPMG, ASPS, DEF, GLTX, HISS, RPLB, RPMB, MAP, PRFA, RPSR, SELA, GLNS, CYSS, FMT, RPSF
KEGG_PATHWAY	ecc00970:Aminoacyl-tRNA biosynthesis	SELA, ASPS, GLTX, HISS, GLNS, CYSS, FMT
UP_SEQ_FEATURE	short sequence motif:"KMSKS" region	GLTX, GLNS, CYSS

UP_SEQ_FEATURE	short sequence motif:"HIGH" region	GLTX, GLNS, CYSS
UP_SEQ_FEATURE	binding site:ATP	PURT, GLTX, GLNS, CYSS, ENVZ, PGK
Annotation Cluster 37	Enrichment Score: 2.2459984921705636	
Category	Term	Genes
GOTERM_BP_FAT	GO:0030258~lipid modification	MHPC, PRPR, MHPA, MHPB, MURG, SDAB, PRPE, FADL, PRPB
GOTERM_BP_FAT	GO:0006631~fatty acid metabolic process	FABD, FABF, PRPE, PRPB, MHPC, PRPR, FABG, MHPA, FABH, MHPB, ACCB, ACPH, SDAB, FADL
Annotation Cluster 38	Enrichment Score: 2.183676519902815	
Category	Term	Genes
SP_PIR_KEYWORDS	cell wall biogenesis/degradation	FTSW, MRCB, GLMU, MRDA, MURG, MURI, FTSI, PBPG, DDLB, ALR, AMPD, AMIA
SP_PIR_KEYWORDS	cell shape	FTSW, MRCB, GLMU, YFGA, MRDA, MURG, MURI, FTSI, PBPG, DDLB, ALR
GOTERM_BP_FAT	GO:0000271~polysaccharide biosynthetic process	FTSI, BCSA, WAAU, FTSW, MRCB, LPXL, RFAZ, RFAY, PBPG, GALE, RCSF, LPXH, GLGB, RFAI, RFAJ, WCAC, DDLB, ALR, KDSB, RFC, CLD, WZXC, GLGP, GLMU, MRDA, MURG, MURI, GLF, AMIA, RFBX
GOTERM_BP_FAT	GO:0005976~polysaccharide metabolic process	YEGX, FTSI, BCSA, AMPD, WAAU, FTSW, MRCB, LPXL, RFAZ, RFAY, PBPG, GALE, RCSF, LPXH, GLGB, RFAI, RFAJ, DDLB, WCAC, ALR, KDSB, CLD, RFC, WZXC, YAGH, GLGP, GLMU, MRDA, MURG, MURI, GLF, AMIA, RFBX
Annotation Cluster 39	Enrichment Score: 2.177248588246868	
Category	Term	Genes
SP_PIR_KEYWORDS	nadp	ASD, YDBC, DKGB, ILVC, METL, PROC, FOLA, FABG, GOR, RIBD, FOLM, NEMA, FOLD
GOTERM_MF_FAT	GO:0050661~NADP or NADPH binding	FOLA, ASD, GOR, RIBD, ILVC, METL
UP_SEQ_FEATURE	nucleotide phosphate-binding region:NADP	FOLA, FABG, YDBC, RIBD, DKGB, METL
Annotation Cluster 40	Enrichment Score: 2.151861868815369	
Category	Term	Genes
SP_PIR_KEYWORDS	nickel	HYCI, HYBC, NIKD, NIKB, NIKC, SURE, YFAU, NIKR, NIKA
SP_PIR_KEYWORDS	ion transport	FHUC, GFCE, ENTF, FHUD, NANC, ZITB, FEOB, FES, YFEO, TRKH, YJCE, ZUPT, LAMB, NMPC, FHUB, YDAN, CUSA, BTUB, FHUA
SP_PIR_KEYWORDS	iron transport	FHUC, ENTF, FHUD, FEOB, FES, TONB, FHUB, FHUA
GOTERM_BP_FAT	GO:0006811~ion transport	GFCE, FHUC, FHUD, NANC, ZITB, YFEH, PSTC, FES, YGHZ, YFEO, PSTB, YJCE, TRKH, TTDT, YEBZ, UIDB, BFR, LAMB, NMPC, NIKD, NIKB, PHOU, NIKC, YCHM, NARK, FHUB, BTUB, FHUA, ENTF, SSEA, FEOB, TONB, ZUPT, CITT, CYST, YAAJ, YDAN, CUSA, RHO

Annotation Cluster 41	Enrichment Score: 2.1404886401160224	
Category	Term	Genes
SP_PIR_KEYWORDS	gluconate utilization	GNTP, IDNT, IDNR, IDNK

Annotation Cluster 42	Enrichment Score: 2.0870405775145575	
Category	Term	Genes
SP_PIR_KEYWORDS	Flavoprotein	HCR, YDDH, MNMG, GCL, DUSC, WRBA, MIOC, MHPA, GOR, FIXC, FIXB, PYRD, PHR, DLD, GLF, FLDA, NEMA
SP_PIR_KEYWORDS	FAD	HCR, MHPA, GOR, FIXC, FIXB, DLD, PHR, MNMG, GLF, GCL, DUSC
UP_SEQ_FEATURE	nucleotide phosphate-binding region:FAD	MHPA, GOR, FIXB, PHR, MNMG, GLF
GOTERM_MF_FAT	GO:0050660~FAD binding	GOR, FIXB, DLD, MNMG, DUSC, AEGA

Annotation Cluster 43	Enrichment Score: 2.08637279835233	
Category	Term	Genes
KEGG_PATHWAY	ecj01040:Biosynthesis of unsaturated fatty acids	FABG, TESA, TESB

Annotation Cluster 44	Enrichment Score: 2.014401750999762	
Category	Term	Genes
SP_PIR_KEYWORDS	cell cycle	FTSW, KILR, HFLB, MURG, FTSZ, FTSI, YIIU, SLMA, YIHA, ACRE, FTSQ
SP_PIR_KEYWORDS	cell division	FTSW, FLK, KILR, HFLB, MURG, FTSZ, FTSI, YIIU, SLMA, YIHA, ACRE, FTSQ

Annotation Cluster 45	Enrichment Score: 2.001922235426106	
Category	Term	Genes
SP_PIR_KEYWORDS	phage recognition	LAMB, FADL, TONB, BTUB, FHUA
GOTERM_BP_FAT	GO:0009581~detection of external stimulus	LAMB, FADL, TONB, BTUB, FHUA

Annotation Cluster 46	Enrichment Score: 1.9872215232081591	
Category	Term	Genes
SP_PIR_KEYWORDS	rrna processing	RUMA, YBEA, RSMF, RLUC, RLUD, RLUA, RLUB, RLMF, RSUA, GIDB, RRMJ
GOTERM_BP_FAT	GO:0006364~rRNA processing	RUMA, YBEA, RSMF, RLUC, RNC, RLUD, RLUA, RLUB, RLMF, RSUA, GIDB, RRMJ
GOTERM_BP_FAT	GO:0016072~rRNA metabolic process	RUMA, YBEA, RSMF, RLUC, RNC, RLUD, RLUA, RLUB, RLMF, RSUA, GIDB, RRMJ

Annotation Cluster 47	Enrichment Score: 1.8585074544206475	
Category	Term	Genes
GOTERM_BP_FAT	GO:0006261~DNA-dependent DNA replication	TOPA, TOPB, DNAC, GYRB, MIOC, RECF, DNAQ, HOLD, HDA, ROB, HOLB, DINB, PRIA, PRIB, RDGC
KEGG_PATHWAY	eum03440:Homologous recombination	DNAQ, HOLD, HOLB, RUVC, PRIA, PRIB, RECF
KEGG_PATHWAY	eco03430:Mismatch repair	XSEA, DNAQ, HOLD, HOLB, XSEB
GOTERM_MF_FAT	GO:0004527~exonuclease activity	XSEA, DNAQ, HOLD, RBN, HOLB, XSEB, RPH
GOTERM_BP_FAT	GO:0006260~DNA replication	TOPA, TOPB, DNAC, GYRB, MIOC, RECF, DNAQ, DIAA, HOLD, HDA, HOLB, ROB, DINB, PRIA, PRIB, RDGC
SP_PIR_KEYWORDS	exonuclease	XSEA, DNAQ, XSEB
GOTERM_BP_FAT	GO:0006259~DNA metabolic process	DNAC, INTA, GYRB, DNAQ, HOLD, HDA, HOLB, ROB, YEGI, DINB, RUVC, YRDD, RECT, XSEA, YBAV, MUG, TOPA, TOPB, INSH, MIOC, RECF, XSEB, YBCK, DIAA, ENDA, PHR, HSDR, PRIA, PRIB, YKFG, RDGC

Annotation Cluster 48	Enrichment Score: 1.849601035463412	
Category	Term	Genes
SP_PIR_KEYWORDS	activator	CADC, GADE, EVGA, NHAR, AAER, CSPH, ARCA, ALLS, TORR, PRPR, DHAR, DPIA, RHAR, MELR, ZRAR, RHAS, ILVY, OMPR, BASR
SP_PIR_KEYWORDS	dna-binding	YKGD, CSPH, CDAR, HUPB, YOAA, YGIT, ROB, DINB, ZUR, RECT, YJGJ, EVGA, NHAR, TOPA, TOPB, HSLR, AAER, ARCA, YPDC, RECF, NIKR, RACR, YFHA, MELR, HSDR, SLMA, BASR, YFHH, ASCG, GADE, YTFA, CADC, XYLR, NRDR, CSIR, ALLS, DPS, DHAR, DPIA, RHAR, RPOS, MTLR, HDFR, RHAS, HFQ, CREB, RCNR, RPOH, MUG, INSH, YBIH, DGOR, UIDR, RUTR, IDNR, RPON, GALR, YHAJ, TORR, PRPR, YIDZ, PHR, BAER, ZRAR, ILVY, OMPR, PRIA, PRIB
SP_PIR_KEYWORDS	Transcription	YKGD, CSPH, CDAR, YGIT, HYCA, ZUR, YJGJ, EVGA, NHAR, AAER, NUSB, ARCA, YPDC, NIKR, RACR, YFHA, MELR, SLMA, BASR, YFHH, ASCG, GADE, YTFA, CADC, XYLR, NRDR, CSIR, ALLS, DHAR, DPIA, RHAR, RPOS, MTLR, HDFR, RHAS, CREB, RPOZ, RCNR, RPOH, YBIH, DGOR, UIDR, RUTR, IDNR, GALR, RPON, YHAJ, TORR, PRPR, YIDZ, BAER, PCNB, ZRAR, ILVY, OMPR, RHO
SP_PIR_KEYWORDS	transcription regulation	YKGD, CSPH, CDAR, YGIT, HYCA, ZUR, PHOU, YJGJ, NHAR, EVGA, AAER, NUSB, ARCA, YPDC, NIKR, RACR, YFHA, MELR, SLMA, BASR, YFHH, ASCG, GADE, YTFA, CADC, XYLR, NRDR, CSIR, ALLS, DHAR, DPIA, RHAR, RPOS, MTLR, HDFR, RHAS, CREB, RCNR, RPOH, YBIH, DGOR, UIDR, RUTR, IDNR, GALR, RPON, YHAJ, TORR, PRPR, YIDZ, BAER, ZRAR, ILVY, OMPR, RHO

Annotation Cluster 49	Enrichment Score: 1.8394338687546952	
Category	Term	Genes

GOTERM_MF_FAT	GO:0042280~cell surface antigen activity, host-interacting	RFAI, RFAJ, KDSB, RFC, NANA, CLD, WAAU, LPXL, GLMU, RFAZ, RFAY, GLF, GALE, COAD, RCSF, LPXH, RFBX
GOTERM_BP_FAT	GO:0016051~carbohydrate biosynthetic process	NANE, FTSI, BCSA, NAGE, YTJC, NANA, WAAU, FTSW, MRCB, LPXL, NANT, RFAZ, NAGB, RFAY, NAGA, PBPG, SDAB, SDAA, GALE, NANK, LPXH, RCSF, PGK, GLGB, RFAI, RFAJ, DDLB, WCAC, ALR, KDSB, CLD, RFC, WZXC, GLGP, GPSA, GLMU, MRDA, MURG, MURI, AGAW, GLF, AMIA, RFBX, EPD
SP_PIR_KEYWORDS	lipopolysaccharide biosynthesis	RFAI, RFAJ, WCAC, KDSB, RFC, CLD, WZXC, WAAU, LPXL, RFAZ, RFAY, GLF, RFBX

Annotation Cluster 50	Enrichment Score: 1.703010602357233	
Category	Term	Genes
SP_PIR_KEYWORDS	s-adenosyl-l-methionine	YBEA, YJJW, RSMF, CYSG, PCM, HEMX, RRMJ, RUMA, METH, YBIY, MOAA, YGCF, RLMF, GIDB, HEMN
SP_PIR_KEYWORDS	rrna processing	RUMA, YBEA, RSMF, RLUC, RLUD, RLUA, RLUB, RLMF, RSUA, GIDB, RRMJ
SP_PIR_KEYWORDS	methyltransferase	YBEA, RSMF, CYSG, RRMA, MNMA, PCM, HEMX, RRMJ, RUMA, YAFS, METH, HSDR, GCVT, RLMF, FMT, THYA, GIDB

Annotation Cluster 51	Enrichment Score: 1.6821235167587012	
Category	Term	Genes
GOTERM_BP_FAT	GO:0051186~cofactor metabolic process	NFUA, NAPA, MOG, BISC, METH, HEMB, MOAD, HEMC, HEMD, GOR, MOAA, HEMG, HEMH, GALE, GLTA, HEMN, SUFD, MENB, MENC, ENTF, NADC, TALB, TESB, SUFB, MENE, COAE, CYSG, PABC, GARR, GLYA, HEMX, FOLA, UBIB, GSHA, COAD, FOLE, EPD, FOLD
GOTERM_BP_FAT	GO:0019438~aromatic compound biosynthetic process	PABC, NAPA, GLYA, WRBA, MOG, FOLA, MOAD, MOAA, TYRB, AROG, DXS, AROD, FOLE, FOLD
GOTERM_BP_FAT	GO:0009396~folic acid and derivative biosynthetic process	FOLA, METH, PABC, GLYA, FOLE, FOLD
GOTERM_BP_FAT	GO:0006730~one-carbon metabolic process	YBEA, FOLA, RSMF, FOLM, GLYA, FOLE, YCBB, FOLD, RRMJ

Annotation Cluster 52	Enrichment Score: 1.6385975772330281	
Category	Term	Genes
SP_PIR_KEYWORDS	one-carbon metabolism	FOLA, FOLM, GLYA, FOLE, FOLD
GOTERM_MF_FAT	GO:0016646~oxidoreductase activity, acting on the CH-NH group of donors, NAD or NADP as acceptor	FOLA, FOLM, FOLD, PROC

Annotation Cluster 53	Enrichment Score: 1.5870917155948252	
Category	Term	Genes
KEGG_PATHWAY	eum00633:Trinitrotoluene degradation	HYBC, HYBO, NEMA

Annotation Cluster 54	Enrichment Score: 1.583393939620284	
Category	Term	Genes
SP_PIR_KEYWORDS	gluconeogenesis	SDAB, FBAA, SDAA, PGK
GOTERM_BP_FAT	GO:0006094~gluconeogenesis	SDAB, SDAA, YTJC, EPD, PGK

Annotation Cluster 55	Enrichment Score: 1.5579182173593675	
Category	Term	Genes
SP_PIR_KEYWORDS	cobalt	USHA, YJJG, GLMU, METH, GARL, EUTB, SURE, MAP
KEGG_PATHWAY	eum00760:Nicotinate and nicotinamide metabolism	USHA, YJJG, NADC, SURE

Annotation Cluster 56	Enrichment Score: 1.5198068260318203	
Category	Term	Genes
SP_PIR_KEYWORDS	cell outer membrane	GFCE, NANC, SFMD, NLPE, YPJA, BAMD, HTRE, LAMB, NMPC, YBGQ, BAMB, FADL, RCSF, FHUA, BTUB
SP_PIR_KEYWORDS	Porin	GFCE, NANC, LAMB, NMPC, BTUB
GOTERM_CC_FAT	GO:0046930~pore complex	GFCE, NANC, LAMB, NMPC, BTUB

Annotation Cluster 57	Enrichment Score: 1.4735709496887637	
Category	Term	Genes
KEGG_PATHWAY	ecj03070:Bacterial secretion system	SECB, SECA, GSPL, GSPM, TATA, YIDC, TATE
SP_PIR_KEYWORDS	protein transport	HOFB, SECB, SECA, HOFC, LOLA, TATA, TONB, TATE
GOTERM_MF_FAT	GO:0008565~protein transporter activity	HOFC, YBHG, GSPL, YIAV, LOLA, MDTE, TATA, TONB, MACA, ACRE, TATE
GOTERM_BP_FAT	GO:0008104~protein localization	HOFC, YBHG, GSPL, TATA, TONB, TATE, SECB, SECA, LOLA, YIAV, MDTE, MACA, YIDC, ACRE
GOTERM_BP_FAT	GO:0045184~establishment of protein localization	HOFC, YBHG, GSPL, TATA, TONB, TATE, SECB, SECA, LOLA, YIAV, MDTE, MACA, ACRE

Annotation Cluster 58	Enrichment Score: 1.4556707499032788	
Category	Term	Genes

KEGG_PATHWAY	ecd00770:Pantothenate and CoA biosynthesis	YAGF, ACPH, COAE, COAD, ILVC

Annotation Cluster 59	Enrichment Score: 1.4455478684054628	
Category	Term	Genes
SP_PIR_KEYWORDS	fmn	YDDH, HEMG, PYRD, SSUD, FLDA, NEMA, MIOC, WRBA
INTERPRO	IPR008254:Flavodoxin/nitric oxide synthase	HEMG, FLDA, MIOC, WRBA
UP_SEQ_FEATURE	domain:Flavodoxin-like	HEMG, FLDA, MIOC, WRBA

Annotation Cluster 60	Enrichment Score: 1.425424554630042	
Category	Term	Genes
GOTERM_BP_FAT	GO:0008615~pyridoxine biosynthetic process	PDXY, SELA, PDXK, DXS, EPD
GOTERM_BP_FAT	GO:0042819~vitamin B6 biosynthetic process	PDXY, SELA, PDXK, DXS, EPD

Annotation Cluster 61	Enrichment Score: 1.4162857302152931	
Category	Term	Genes
KEGG_PATHWAY	ecj00030:Pentose phosphate pathway	TALB, PRS, FBAA, EDD, GLPX, IDNK, PFKB
KEGG_PATHWAY	ecd00051:Fructose and mannose metabolism	MAK, MTLD, FBAA, GLPX, PFKB, YFAU
KEGG_PATHWAY	ecd00010:Glycolysis / Gluconeogenesis	FBAA, YTJC, GLPX, GLK, PFKB, PGK
GOTERM_BP_FAT	GO:0019318~hexose metabolic process	GLGB, YJJW, PDXK, TALB, FBAA, GARR, BCSA, GALR, YTJC, GLGP, RHAM, PDXY, YBIY, RHAR, SDAB, RHAS, SDAA, GALE, EPD, GLK, PGK, PFKB

Annotation Cluster 62	Enrichment Score: 1.411893391131368	
Category	Term	Genes
SP_PIR_KEYWORDS	riboflavin biosynthesis	RIBD, RIBE, RIBA

Annotation Cluster 63	Enrichment Score: 1.373730065057539	
Category	Term	Genes
SP_PIR_KEYWORDS	transcription initiation	RPOH, RPOS, RPON
GOTERM_BP_FAT	GO:0032774~RNA biosynthetic process	RPOH, DNAC, RPOS, NUSB, RPON, PRIA, PRIB, RPOZ, RHO
GOTERM_BP_FAT	GO:0006351~transcription, DNA-dependent	RPOH, RPOS, NUSB, RPON, RPOZ, RHO

SP_PIR_KEYWORDS	Sigma factor	RPOH, RPOS, RPON
